# The trajectory of gait development in mice

**DOI:** 10.1002/brb3.1636

**Published:** 2020-04-24

**Authors:** Shyam K. Akula, Katherine B. McCullough, Claire Weichselbaum, Joseph D. Dougherty, Susan E. Maloney

**Affiliations:** ^1^ Department of Psychiatry Washington University School of Medicine St. Louis MO USA; ^2^ Department of Genetics Washington University School of Medicine St. Louis MO USA; ^3^ Harvard‐MIT MD/PhD Program Harvard Medical School Boston MA USA; ^4^ Intellectual and Developmental Disabilities Research Center Washington University School of Medicine St. Louis MO USA

**Keywords:** development, DigiGait, gait, motor function, mouse, mouse strains

## Abstract

**Objective:**

Gait irregularities are prevalent in neurodevelopmental disorders (NDDs). However, there is a paucity of information on gait phenotypes in NDD experimental models. This is in part due to the lack of understanding of the normal developmental trajectory of gait maturation in the mouse.

**Materials and methods:**

Using the DigiGait system, we have developed a quantitative, standardized, and reproducible assay of developmental gait metrics in commonly used mouse strains that can be added to the battery of mouse model phenotyping. With this assay, we characterized the trajectory of gait in the developing C57BL/6J and FVB/AntJ mouse lines.

**Results:**

In both lines, a mature stride consisted of 40% swing and 60% stance in the forelimbs, which mirrors the mature human stride. In C57BL/6J mice, developmental trajectories were observed for stance width, paw overlap distance, braking and propulsion time, rate of stance loading, peak paw area, and metrics of intraindividual variability. In FVB/AntJ mice, developmental trajectories were observed for percent shared stance, paw overlap distance, rate of stance loading, and peak paw area, although in different directions than C57 mice. By accounting for the impact of body length on stride measurements, we demonstrate the importance of considering body length when interpreting gait metrics.

**Conclusion:**

Overall, our results show that aspects of mouse gait development parallel a timeline of normal human gait development, such as the percent of stride that is stance phase and swing phase. This study may be used as a standard reference for developmental gait phenotyping of murine models, such as models of neurodevelopmental disease.

## Graphical abstract

To fill the gap in our understanding of the normal developmental trajectory of gait in the mouse, we characterized gait in two mouse strains across development. Our results revealed metrics that best reflect gait maturation in both strains and underscore the importance of considering body length when interpreting gait metrics. Overall, our results show that aspects of mouse gait development parallel a timeline of normal human gait development.

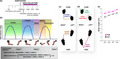

## 1 INTRODUCTION

Consistent gait is a marker of coordination and normal neurological function. Gait disturbances are the hallmark phenotype of diseases like cerebral palsy and Parkinsonism and can also be observed in acute states of neurological dysfunction, such as alcohol intoxication (Nieuwboer et al., 2001; Vonghia et al., 2008; Wren, Rethlefsen, & Kay, 2005). Subtle gait differences are also a feature of many neurodevelopmental disorders (NDDs), such as autism spectrum disorders and Williams–Beuren Syndrome (Hocking, Rinehart, McGinley, & Bradshaw, 2008; Kindregan, Gallagher, & Gormley, 2015). Gait disturbances in NDDs may be a consequence of underlying alterations in circuit function or in circuit maturation. Mice are often used to study the function of normal circuits, their development, and their disruption in disease states, as many such circuits are conserved between mouse and human. While gait has been studied in adult mouse models of disease, the trajectory of gait maturation has not been quantitatively characterized in mice. Understanding development of gait in the mouse may help with characterization of mouse models of NDDs, as disruption in gait in NDDs is likely a result of altered maturation of CNS circuitry that produces gait.

Gait is made up of strides that comprise a stance phase with the foot in contact with the ground and a swing phase with the foot off the ground. In humans, the composition of gait differs in early development compared to adulthood; markers of such gait maturation include a decrease in double support time (both feet in stance simultaneously), a decrease in the swing/stance ratio, a decrease in the number of strides per second (cadence) and increased stride length. These latter two metrics are driven by limb lengthening and greater limb stability (Sutherland, Olshen, Cooper, & Woo, 1980) and may not reflect a maturation of the neural circuits underlying gait production. In mice, although gait has been studied in mature mice frequently, a comprehensive quantitative description of changes in gait parameters from immature to mature, analogous to those measured in humans, is currently lacking.

Modern image and video analysis allow for computerized gait analysis systems that expand the quantifiable gait parameters to include temporal and postural metrics alongside the spatial metrics produced with traditional footprint analysis on ink and paper. One such system is the DigiGait (Mouse Specifics, Boston, MA), a treadmill system with a transparent belt that allows creation of digital “footprints” to analyze posture and kinematics through capturing images of the mouse underside and paws. Leveraging this system, we can comprehensively define spatiotemporal and postural aspects of gait, as well as the intraindividual variability within these metrics. Further, we can identify which metrics are influenced by changing body size and thus would be less significant from the point of view of studying circuits. Studying how gait develops will enable us to better understand how behavioral motor circuits are refined and matured, and, thus, guide future studies into abnormalities in circuit function and maturation in NDD.

To this end, we characterized normative gait in two inbred mouse strains, C57BL/6J and FVB/AntJ, across development using the DigiGait gait analysis system. Our assay begins at postnatal day (P)21, the youngest age at which the mice could reliably complete the treadmill assay, and an age that corresponds to approximately 2–3 years of age in humans in terms of brain development (Gegenhuber & Tollkuhn, 2019; Semple, Blomgren, Gimlin, Ferriero, & Noble‐Haeusslein, 2013). The assay continued through the juvenile stage, covering a window of time during which substantial maturation occurs in human gait (Pediatric Musculoskeletal Matters International, n.d.; Sutherland et al., 1980). We present below how spatiotemporal and postural gait metrics and their intraindividual variability change with and without the influence of body size, an important confounder in measurements of gait parameters. These data provide a detailed examination of gait maturation in the mouse. Further, they provide an index which can inform interpretations of future studies of altered gait development in mouse models of disease.

## 2 MATERIALS AND METHODS

### 2.1 Animals

All experimental protocols were approved by and performed in accordance with the relevant guidelines and regulations of the Institutional Animal Care and Use Committee of Washington University in St. Louis and were in compliance with US National Research Council's Guide for the Care and Use of Laboratory Animals, the US Public Health Service's Policy on Humane Care and Use of Laboratory Animals, and Guide for the Care and Use of Laboratory Animals. C57BL/6J (C57; https://www.jax.org/strain/000664, RRID:IMSR_JAX:000664) and FVB/AntJ (FVB; https://www.jax.org/strain/004828, RRID:IMSR_JAX:004828) inbred mouse (Mus musculus) strains were used in this study. This FVB substrain is homozygous for the wild type *Pde6b* allele and does not go blind. Twenty‐five (8M, 18F) C57 and 32 (15M, 17F) FVB mice were used. All mice used in this study were maintained and bred in the vivarium at Washington University in St. Louis. For all experiments, adequate measures were taken to minimize any pain or discomfort. The colony room lighting was 12:12 hr light/dark cycle; room temperature (~20–22°C) and relative humidity (50%) controlled automatically. Standard laboratory diet and water were freely available. Pregnant dams were individually housed in translucent plastic cages measuring 28.5 × 17.5 × 12 cm with corncob bedding. Upon weaning at postnatal day (P)21, mice for behavioral testing were group‐housed according to sex.

### 2.2 Gait analysis

#### 2.2.1 Apparatus

Gait data were collected using the DigiGait Imaging System (Mouse Specifics, Inc), an advanced gait analysis system with Ventral Plane Imaging Technology that generates digital paw prints from the animal as it runs on a motorized treadmill (Hampton, Stasko, Kale, Amende, & Costa, 2004). This system has been described in detail previously (Hampton et al., 2004) and is described in Data S1.

#### 2.2.2 Procedure

**FIGURE 1:**
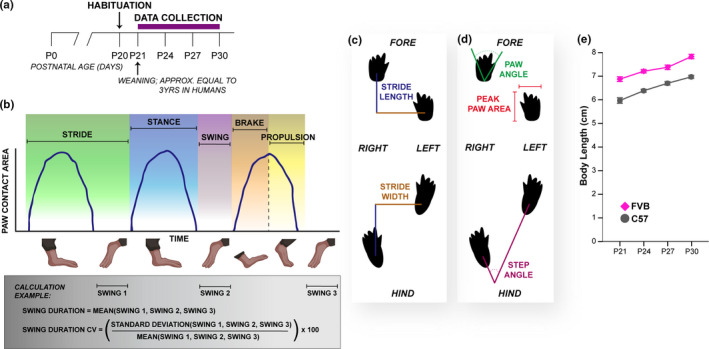
Gait analysis procedure and measurement schematics. (a) Schematic of developmental gait data collection procedure. Purple bar represents duration of data collection. (b) Schematic of paw contact area plots (blue lines) derived by DigiGait software to quantify spatiotemporal gait metrics (represented by different background colors). Below the graph is a cartoon representation of mouse feet during three strides. The gray box provides an example of variable calculations based on these plots. (c) Cartoon of digital mouse footprints with representations of measurements of the spatial metrics stride length (blue) and stride width (brown) measurements. (d) Cartoon of digital mouse footprints with representations of measurements of the postural metrics paw angle (green), step angle (eggplant), and peak paw area (red). (e) Body length measurements for C57 and FVB mice made along the long axis of the mouse from nose to base of tail (data are means ± *SEM*)

Detailed methods are provided in Data S1. Briefly, each mouse was habituated to the apparatus on P20. This consisted of placing the animal on the stationary belt and starting the belt moving at 5 cm/s and slowly increasing the speed until 20 cm/s is reached allowing for at least 30 s of run time. Testing occurred at P21, P24, P27, and P30 (Figure 1a). For these test days, each mouse was placed individually on the apparatus. The belt was started at 10 cm/s until the animal started walking forward. Once the animal reached the front of the alley, the speed was increased to 20 cm/s. Because speed is the greatest influencer of gait, the speed of the treadmill during data collection was kept constant across all ages at 20 cm/s to allow for appropriate comparisons of forced gait across age. Once a usable run was acquired, the belt was stopped and the animal removed to the homecage. Criteria for a usable run included a consistent forward movement with no sliding, jumping, or side drift. All testing occurred during the light phase of the circadian cycle. The belt was cleaned with 70% EtOH between litters or as needed between mice and daily upon completion of testing.

Each video was then processed through the DigiGait Analysis software, as described previously (Hampton et al., 2004) and in Data S1. As the postvideo acquisition processing within the DigiGait software requires some manual corrections/input, we also analyzed inter‐rater reliability for all variables, excluding those metrics with poor reliability. This is described in detail in Data S1. Gait was analyzed by quantifying components of the step cycle, or stride, broken into when a paw has contact with the ground, known as the stance phase, and when it is moving through the air, known as the swing phase (Figure 1b). The stance phase is further broken down into the paw braking phase (heel strike to full stance) and propulsion phase (full stance to toe push off). The DigiGait software extracts the temporal measures from the paw contact area plots derived from the digital footprints (Figure 1b, Table 1), while the spatial and postural measures are derived straight from the digital footprints (Figure 1c,d, Table 1). Each of these measures was calculated as an average across all strides of a trial (see Figure 1b for example, Table 1). The intraindividual variability within many of the measures was also calculated as the coefficient of variance (CV) by dividing the standard deviation of the strides in a trial by the mean of the strides in a trial (see Figure 1b for example). Selection criteria for gait metrics for analysis are found in Data S1 and Figures [Link], [Link], [Link], [Link], [Link], [Link].

**TABLE 1:** Description of gait metrics

Component subtype and metric	Definition	ICC with 95% CI	
Spatial subcomponent		Fore	Hind
Stride frequency	Number of completed strides per second (cadence)	0.975 [0.958, 0.985]	0.989 [0.981, 0.993]
Stride length	Distance covered during one full 'rotation' of a paw through both stance and swing phases	0.986 [0.976, 0.992]	0.991 [0.985, 0.995]
Stance width	Distance between fore or hind limbs during full stanc	0.972 [0.940, 0.987]	0.990 [0.978, 0.995]
Paw overlap distance	Average overlapping distance of ipsilateral paws across successive strides	n/a	0.994 [0.991, 0.997]
Paw placement positioning	The extent of overlap of ipsilateral paws at full stance (reflecting balance)	n/a	0.989 [0.981, 0.994]
Gait symmetry	The ratio of left to right step frequency	n/a	0.610 [0.336, 0.771]
Temporal subcomponent			
Swing duration	Time the paw is not in contact with the belt	0.972 [0.952, 0.983]	0.982 [0.970, 0.990]
Stance duration	Time the paw is in contact with the belt	0.985 [0.974, 0.991]	0.995 [0.991, 0.997]
Brake duration	Time of the braking portion of the stance phase where the paw is initiating contact with the belt though the heel (initial paw contact to full paw contact; immediately follows swing phase)	0.946 [0.908, 0.968]	0.932 [0.883, 0.960]
Propulsion duration	Time of the propelling portion of the stance phase where the paw is lifting off of the belt though the toes (full paw contact to final paw contact; immediately precedes swing phase)	0.954 [0.921, 0.973]	0.980 [0.966, 0.988]
Stance factor	The ratio of left to right stance durations (measure of gait symmetry)	0.949 [0.890, 0.976]	0.932 [0.810, 0.972]
Maximal rate of paw contact change	Maximal rate of paw area contact change during the braking portion of stance (how quickly the paw is loaded on to the belt)	0.838 [0.724, 0.905]	0.775 [0.617, 0.868]
% Stance	Percent of stride that comprises the stance phase	0.971 [0.950, 0.983]	0.981 [0.967, 0.989]
% Swing	Percent of stride that comprises the swing phase	0.971 [0.950, 0.983]	0.981 [0.967, 0.989]
% Hind limb shared Stance time	Percent of stance phase during which both hind limbs are in contact with the belt	n/a	0.982 [0.967, 0.990]
Postural subcomponent			
Absolute paw angle	The angle of the paw with the long axis of the direction of locomotion of the animal (degree of external rotation)	0.909 [0.845, 0.947]	0.948 [0.911, 0.969]
Step angle	The angle between the right and left hind paws due to stride length and stance width	0.898 [0.982, 0.953]	0.995 [0.987, 0.998]
Peak paw area	Area of the paw at full stance	0.751 [0.577, 0.853]	0.783 [0.631, 0.873]
Intraindividual variability parameters			
Coefficient of variance (CV)	A normalized measure of variability calculated as [(standard deviation/mean) × 100]	—	—
	Stride length CV	0.875 [0.787, 0.927]	0.912 [0.849, 0.949]
	Stance width CV	0.960 [0.914, 0.981]	0.935 [0.895, 0.970]
	Swing duration CV	0.905 [0.837, 0.944]	0.909 [0.844, 0.946]
	Paw angle CV	0.853 [0.749, 0.915]	0.830 [0.709, 0.900]
	Step angle CV	0.671 [0.283, 0.848]	0.927 [0.843, 0.966]
	Peak paw area CV	0.660 [0.419, 0.800]	0.980 [0.975, 0.988]

Note

Gait metrics organized by subtype with definitions and intraclass correlation coefficients (ICC) with their 95% confidence intervals used to determine inter‐rater reliability of gait video processing between the measurements produced by two independent experimenters.

### 2.3 Body length quantification

Animal body length was extracted from DigiGait videos using Ethovision v.14 (Noldus Information Technology, RRID:SCR_000441). Body length was measured in each frame and then averaged for analysis. Manual measurements were used for validation of this method. For detailed methods, see Data S1.

### 2.4 Statistical analysis

All statistical analyses were performed using IBM SPSS Statistics software (v.25, RRID:SCR_002865). Prior to analyses, all data were screened for missing values, fit of distributions with assumptions underlying univariate analyses. This included the Shapiro–Wilk test on *z*‐score‐transformed data and Q–Q plot investigations for normality, Levene's test for homogeneity of variance, and boxplot and *z*‐scores (±3.29) investigation for identification of influential outliers. However, no outliers were removed. To limit variability observed in gait studies conducted in a cross‐sectional design (Hillman, Stansfield, Richardson, & Robb, 2009), we performed a longitudinal analysis on the FVB data. The FVB sample was reduced to 19 (7M, 12F) due to a reduced number of quantifiable videos based on selection criteria from these mice at all time points. Longitudinal analysis of C57 data would have resulted in substantial data loss and a reduced sample size of nine; therefore, these data were analyzed in a cross‐sectional design. Means, standard errors, and standard deviations were computed for each measure. Linear mixed modeling (LMM) was used to analyze gait data across juvenile ages, with age as a repeated fixed factor grouped by subject ID and a data structure that follows stride→age→mouse. Statistical results were confirmed with the nonparametric Friedman test for any outcome measure with violations of normality. To examine the influence of body length on gait metrics across age, LMM was again used with body length as a covariate, The Benjamini–Hochberg correction for false discovery rate (FDR; at *q* = 0.1) was used to adjust the critical alpha level for multiple analyses within each strain. Test statistics and other details for each analysis are provided in Tables [Link], [Link], [Link].

## 3 RESULTS

### 3.1 Body length heavily impacted gait measurement

A major consideration during our study was the accompanying change in body length across the age range examined (Figure 1a,e) and adjusting our data to account for the influence of body length. Thus, we analyzed our data both without and with accounting for body length to identify metrics that are heavily influenced by this variable. This also served to identify metrics that are independent of body length and thus best represent gait maturation across this developmental window.

**FIGURE 2:**
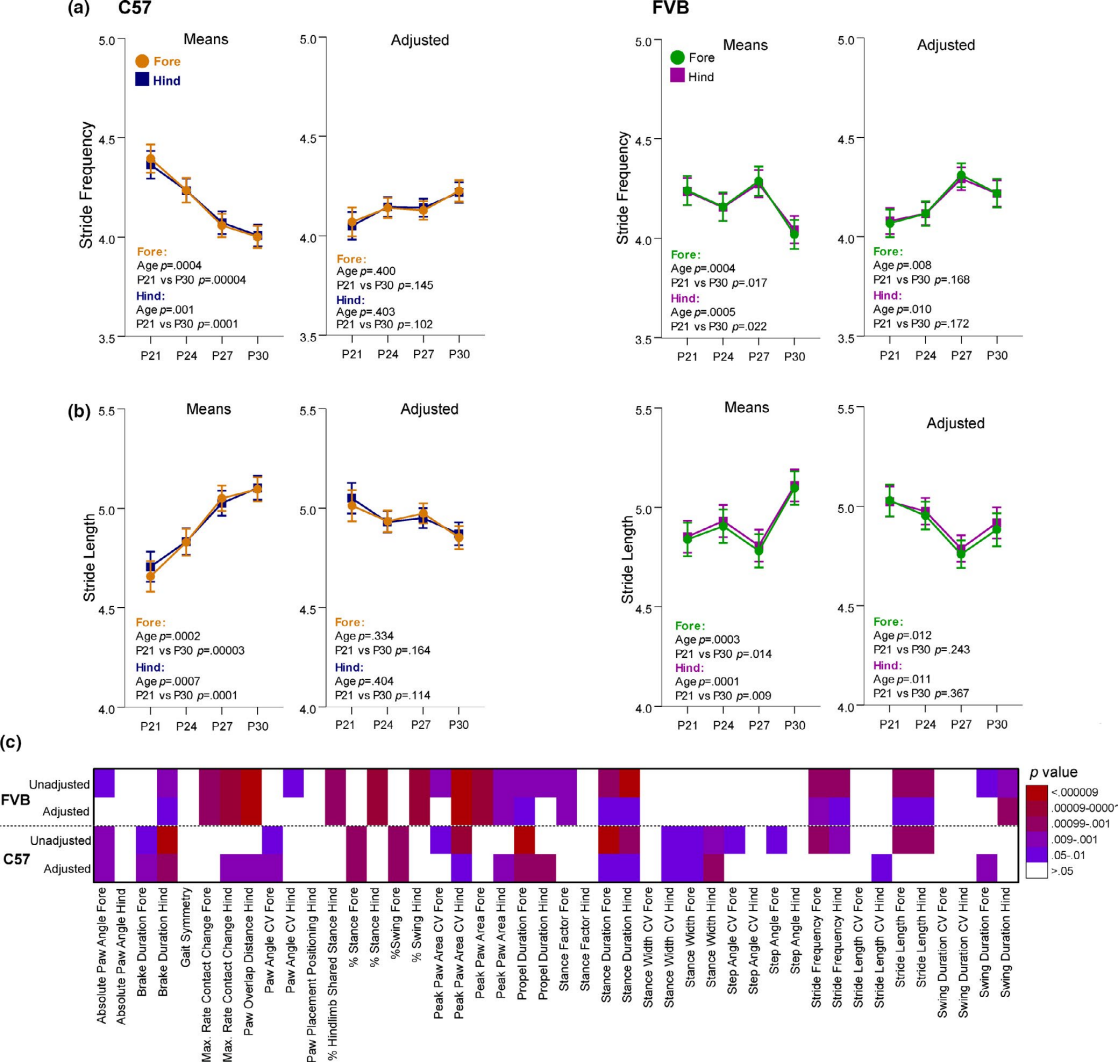
The trajectories of stride frequency and length from P21 to P30 reflected only changes in body length during this time. (a and b) Stride frequency (a) and length (b) raw means and covariate‐adjusted means are presented for both C57 and FVB mice. Both measures for forelimbs and hind limbs appeared to significantly increase with age. However, after adjusting for differences in body length from P21 to P30, age was no longer significantly changing from P21 to P30. Data are means ± *SEM* and covariate‐adjusted means ± *SEM*. (c) Heat map of the significance level (*p* value) of age for each gait metric from both the LMM unadjusted for body length and the LMM adjusted for body length for FVB and C57 mice. LMM, linear mixed modeling

We found several gait metrics appeared to significantly change with juvenile age, but further analysis revealed this change across age was solely due to changes in body length from P21 to P30. Examples of these metrics include stride frequency and stride length in C57 and FVB mice (Figure 2a,b). The remaining metrics that reflect *only* a change in body size but not gait maturation can be found in Figures S7 and S8. These metrics highlight the importance of accounting for changes to body size in gait analysis and that failing to do so may result in erroneous interpretation of gait changes in a model compared to controls. A complete list of gait metrics that were significantly influenced by body length is found in Table 2. However, many of these metrics still followed developmental trajectories after controlling for the effect of body length (Figure 2c) and are discussed below.

**TABLE 2:** Gait metrics significantly influenced by body length

C57	FVB
%—Hind limb Shared Stance—Hind	% Stance—Fore
% Stance—Hind	% Swing—Fore
% Swing—Hind	Maximal Rate of Paw Contact Change— Hind
Absolute Paw Angle—Hind	Paw Overlap Distance
Brake Duration—Fore	Peak Paw Area CV—Fore
Maximal Rate of Paw Contact Change—Fore and Hind	Propulsion Duration—Fore and Hind
Paw Angle CV—Fore	Stance Duration—Fore and Hind
Paw Overlap Distance—Hind	Stride Frequency—Fore and Hind
Paw Placement Positioning—Hind	Stride Length—Fore and Hind
Peak Paw Area—Fore and Hind	Swing Duration—Fore and Hind
Propulsion Duration—Hind	
Stance Duration—Fore and Hind	
Stance Width—Hind	
Stride Frequency—Fore and Hind	
Stride Length—Fore and Hind	
Stride Length CV—Hind	
Swing Duration—Fore	

### 3.2 Juvenile C57 mice exhibited developmental trajectories of stride phase proportion, distinct spatial, and variability metrics, and stance subcomponents

**FIGURE 3:**
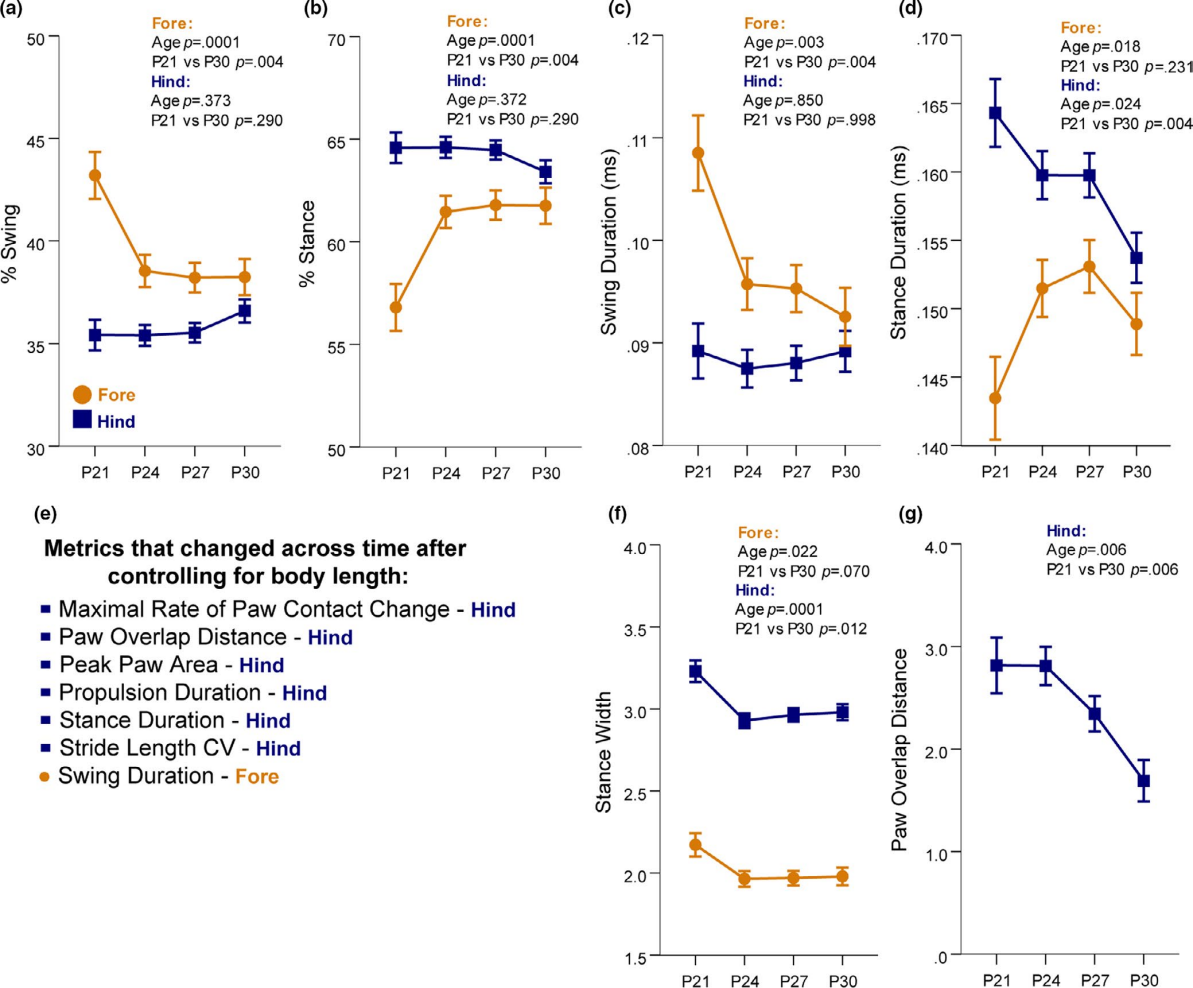
Percent and duration of swing and stance stride phases, width of stance, and distance of ipsilateral paw overlap changed in C57 mice from P21 to P30. (a and b) At P24 in C57 mice, the % of stride that is the swing phase decreased to 40% at P24, while the percent of stride that is stance increased to 60% in forelimbs. The hind limbs were stable in these measures. (c) Forelimb swing duration significantly decreased and the hind limb swing duration remained constant. (d) Forelimb stance duration increased at P24, while the hind limb stance duration decreased. (e) List of gait metrics which appeared stable from P21 to P30 in the raw data, but actually changed across development after adjusting for the influence of body length. (f) The width of stance significantly narrowed for both forelimbs and hind limbs at P24. (g) The overlapping distance between ipsilateral paws across successive strides decreased at P27 and P30. Data are covariate‐adjusted means ± *SEM*

The developmental trajectory of gait in P21–P30 mice was observed in the swing and stance phases of stride, specific spatial and intraindividual variability metrics, and stance subcomponents. Percent of stride that is made up of the swing and stance phases of stride were examined because of the changes to these measurements that characterize gait maturation in humans (Sutherland et al., 1980). In forelimbs, % swing decreased (Figure 3a) and % stance increased (Figure 3b) to achieve relative proportions of stride of 40% and 60%, respectively, by P24. The changes in percent of stride phases were reflected in changes to absolute duration of these stride phases. Forelimb swing duration decreased (Figure 3c) while the forelimb stance duration increased (Figure 3d) at P24. Hind limb stance duration decreased across this developmental window. Stance duration is particularly interesting because without controlling for changes in body length, hind limb stance duration appeared to increase from P21 to P30 (Figure S9). However, after controlling for body length in our model, the true trajectory was revealed to decrease, once again highlighting the importance of accounting for body length differences. Other gait measurements in C57 mice that were revealed to change with age *only after* controlling for body length differences are listed in Figure 3e. In addition, all metrics that remained stable from P21 to P30 are listed in Table 3.

**TABLE 3:** Stable gait metrics from P21 to P30

C57	FVB
% Hind limb Shared Stance—Hind	% Stance—Fore
% Stance—Hind	% Swing—Fore
% Swing—Hind	Absolute Paw Angle—Fore and Hind
Absolute Paw Angle—Hind	Brake Duration—Fore and Hind
Gait Symmetry	Gait Symmetry
Maximal Rate of Paw Contact Change—Fore	Paw Angle CV—Fore and Hind
Paw Angle CV—Hind	Paw Placement Positioning
Paw Placement Positioning—Hind	Peak Paw Area CV—Fore
Peak Paw Area—Fore	Propulsion Duration—Hind
Peak Paw Area CV—Fore	Stance Duration—Hind
Stance Factor—Fore and Hind	Stance Factor—Hind
Stance Width CV—Fore	Stance Width—Fore and Hind
Step Angle—Fore & Hind	Stance Width CV—Fore and Hind
Step Angle CV—Fore and Hind	Step Angle—Fore and Hind
Stride Frequency—Fore and Hind	Step Angle CV—Fore and Hind
Stride Length—Fore and Hind	Stride Length CV—Fore and Hind
Stride Length CV—Fore	Swing Duration—Fore
Swing Duration—Hind	Swing Duration CV—Fore and Hind
Swing Duration CV—Fore and Hind	

The spatial components of C57 gait that were revealed to have a significant developmental trajectory were stance width and paw overlap distance. The stance width of both the forelimbs and hind limbs decreased at P24 and then remained stable through P30 (Figure 3f). The distance that is overlapping between ipsilateral paws across successive strides decreased at P27 and P30 (Figure 3g), which was only observed after controlling for any effect of body size differences between ages. Thus, a mature gait in a C57 mouse was reflected spatially by a narrower stance and less overlap of ipsilateral limbs.

**FIGURE 4:**
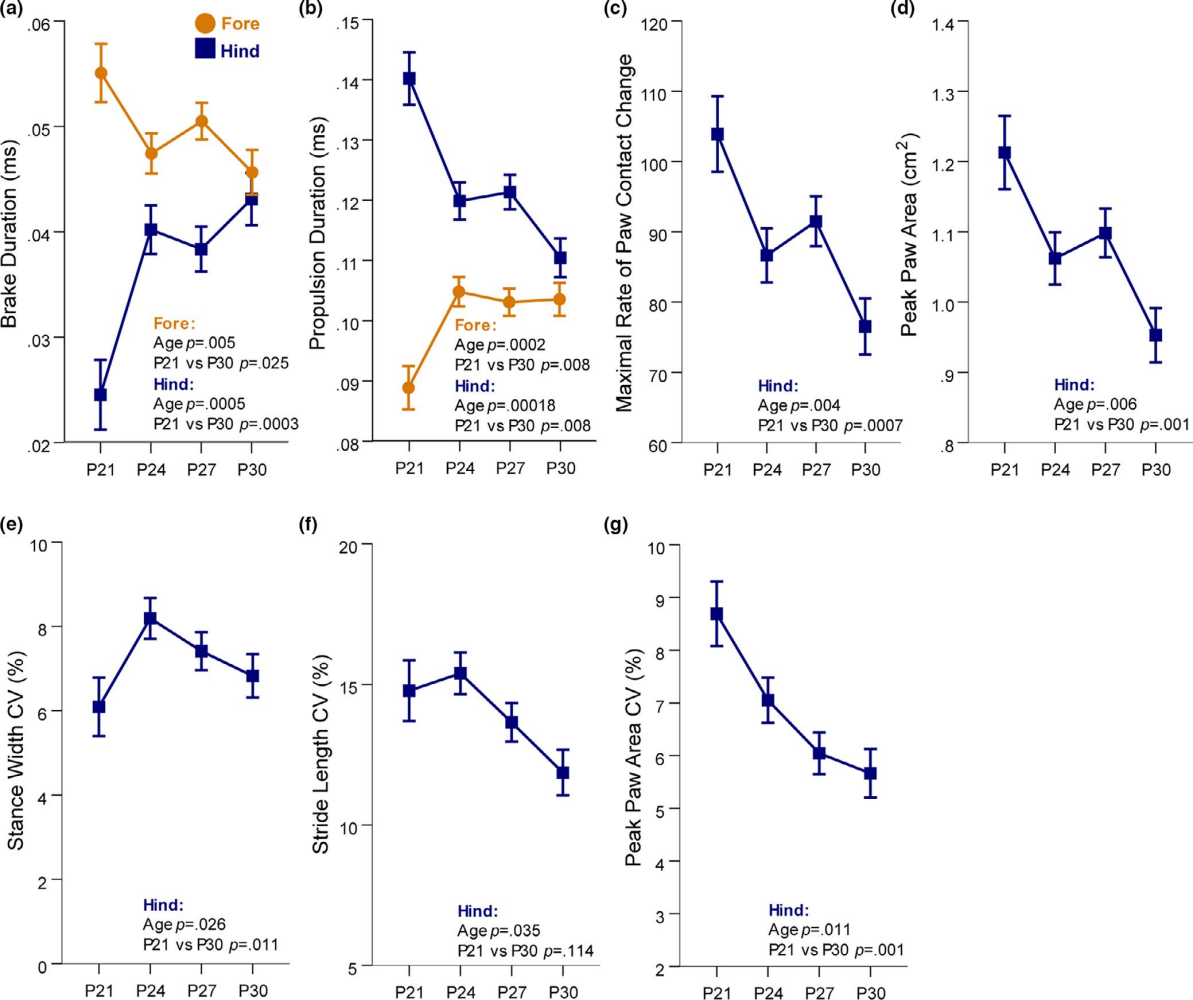
Stance subcomponents that represent how the paw is loaded and unloaded during the stance phase as well as intraindividual variability metrics exhibited change across the juvenile developmental window C57 mice. (a) Braking duration decreased for forelimbs and increased for hind limbs reach a comparable value at P30. (b) Propulsion duration increased for forelimbs and decreased for hind limbs until they reach a comparable level at P30. (c) The maximal rate of paw contact change or how quickly the paw is loaded into the stance phases significantly decreased from P21 to P30. (d) The peak paw area of the hind limbs measured at full stance significantly decreased from P21 to P30. (e) The variability of stance width increased from P21 to P24. (f and g) The variability in stride length (f) and peak paw area (g) significantly decreased from P21 to P30. Data are covariate‐adjusted means ± *SEM*

Stance subcomponents that represent how the paw is loaded and unloaded during the stance phase exhibited change across the juvenile developmental window. For the fore‐ and hind limbs, the brake duration and propulsion duration showed opposite developmental patterns. The duration of brake performed by the forelimbs decreased while the duration of propulsion performed by the forelimbs increased (Figure 4a,b). The opposite was displayed by the hind limbs: Brake duration increased while propulsion duration decreased. By P30, both sets of limbs were nearing an equal contribution to braking and propulsion. The maximal rate of paw contact change, or how quickly the paw was loaded onto the belt, also decreased for the hind limbs across this developmental window (Figure 4c). The slowing speed at which the hindpaw is loaded onto the belt is likely a major driver of the increased duration of the braking phase. Also observed in the hind limbs was a significant decrease in peak paw area (Figure 4d), which is measured at full stance. The decreases across age to hind limb maximal rate of paw contact change and hind limb peak paw area may reflect a maturing of paw placement on the belt.

These two metrics, maximal rate of paw change and peak paw area, are likely related to the paw size. Thus, it is possible that the decreases in both metrics reflect relatively larger paw size at younger ages compared to body length. That is, the ratio of paw size to body length may decrease with age. To determine whether this is the case, we measured paw lengths in a subset of our sample. We found that hind paw lengths increased with age (Figure S10a) and that paw size is very strongly positively correlated with body length (Figure S10b). Therefore, we believe the change in maximal rate of paw change and peak paw area is not simply reflecting a change in paw to body length ratio, but rather is related to how the mice are loading their paws onto the belt, possibly reflecting a change toward heel‐to‐toe stepping from flat‐footed stepping (Kraan, Tan, & Cornish, 2017).

The developmental trajectory of C57 gait was also observed in aspects of hind limb intraindividual variability. Variability in stance width increased from P21 to P24 (Figure 4e). Variability in stride length decreased at P27 and P30 (Figure 4f), and variability in peak paw area decreased at P24 and P27 (Figure 4g). The postural metric absolute paw angle and its variability, and paw angle CV, did show significant changes across the developmental window measured for the forelimbs only, but the patterns are hard to interpret (Figure S11). These metrics will need to be explored further to better elucidate their patterns across this age period.

### 3.3 Juvenile FVB mice exhibited developmental trajectories of stride phase proportions, spatial paw overlap, and stance subcomponents

**FIGURE 5:**
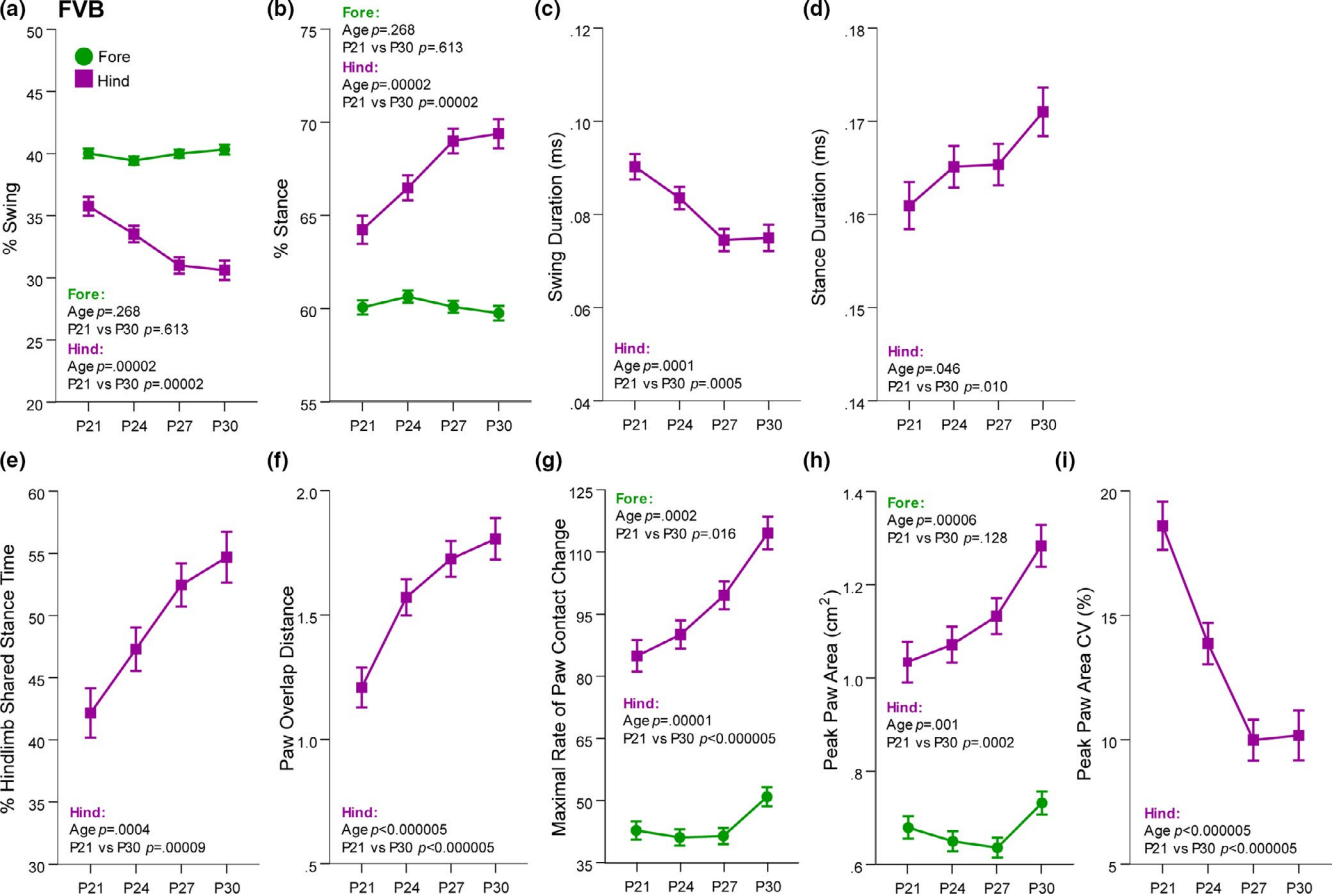
The trajectory of gait in FVB mice from P21 to P30 was reflected in hind limb swing and stance phases, distance of ipsilateral paw overlap, and how the paw is loaded during the stance phase. (a and b) In FVB mice, the % of stride that is the swing phase decreased (a) and the percent of stride that is stance increased (b) in the hind limbs, while the forelimbs remained stable for these measures. (c and d) Absolute swing duration decreased (c) and absolute stance duration increased, yet did not survive FDR correction, (d) in the hind limbs. (e) The percent of time shared in stance by both hind limbs increased from P21 to P30. (f) The overlapping distance between ipsilateral paws across successive strides increased from P21 to P30. (g) The maximal rate of paw contact change, or how quickly the paw is loaded into the stance phases significantly increased from P21 to P30 in both limbs. (h) The peak paw area of the hind limbs increased from P21 to P30. (i) The variability in the peak paw area decreased until P27. Data are covariate‐adjusted means ± *SEM*

The trajectory of gait development of the FVB mice was observed in many of the same metrics as for gait development in C57 mice, and in some different metrics. While C57 mice showed developmental change in % of swing and % stance of stride in their forelimbs, the FVB mice were stable at 40% swing and 60% stance for forelimbs (Figure 5a,b). However, % swing decreased and % stance increased for the hind limbs until P27, although stance duration did not survive FDR correction. Again like the C57 metrics, these changes in percent of stride measures were reflected in changes to the absolute duration of these stride phases. Hind limb swing duration decreased and stance duration increased until P27 and P30, respectively (Figure 5c,d). Unique to FVB mice, the percent of time the hind limbs are both in stance (shared stance) increases until P27 (Figure 5e). These metrics suggest proportions of the different phases of a stride for the hind limbs are mature around P27 in the FVB mouse, while those for the forelimbs are mature before P21.

The spatial component of FVB gait that exhibited a significant developmental trajectory was paw overlap distance. Until P27, the distance overlapped by ipsilateral paws across successive strides increased (Figure 5f). Thus, a mature gait in the FVB mice was reflected by greater overlap of ipsilateral limbs.

Multiple stance subcomponents representing how the paw is loaded during stance phase displayed developmental changes in FVB mice. The maximal rate of paw contact change, or how quickly the paw was loaded onto the belt, increased for both the forelimbs and hind limbs (Figure 5g), as did the peak paw area measured at full stance in the hind limbs (Figure 5h). The variability in peak paw area of the hind limbs decreased until P27 (Figure 5i). We again examined the ratio of paw size to body length over time because both maximal rate of paw change and peak paw area are likely related to the paw size. Like C57 mice, paw length in FVB mice increased with age (Figure S10c) and was strongly positively correlated with body length (Figure S10d). Therefore, we are confident the change in maximal rate of paw change and peak paw area is not simply reflecting a change in paw to body length ratio, but that these data indicate the FVB mice loaded their paws onto the belt more quickly with age, and suggest the FVB mice were gaining greater control over hind paw placement during stance with age.

## 4 DISCUSSION

Gait disruptions can represent a pathological state across many neurological diseases and disorders. These abnormalities can reflect a breakdown of motor control circuits as in neurodegenerative diseases like Parkinson's Disease and Huntington's Disease (Amende et al., 2005; Hausdorff, Cudkowicz, Firtion, Wei, & Goldberger, 1998; Karachi et al., 2010; Laforet et al., 2001; Rao, Muratori, Louis, Moskowitz, & Marder, 2008), or a faulty maturation of motor control circuits as in neurodevelopmental disorders (Jeste, 2011; Mosconi, Wang, Schmitt, Tsai, & Sweeney, 2015). Here, we have quantitatively characterized typical development of multiple gait parameters in C57 and FVB mice in a controlled setting, accounting for major confounding factors in gait analysis.

Examination of gait in these two oft‐used mouse strains revealed a set of gait metrics that change over the P21–P30 developmental period and thus likely reflect aspects of gait maturation. In C57 mice, we observed a change in the percent of swing and stance phase of stride to 40% and 60%, respectively, a stride proportion that mirrors what is observed in the mature human stride (Sutherland et al., 1980). Maturation of the C57 gait also consisted of a narrower stance and less overlap of ipsilateral limbs, and an equalization between limbs of time spent braking and propelling. A mature C57 stride was also achieved through a decrease in the rate at which the hind limbs load into the stance phase, which is likely related to the observed increased duration of hind limb braking, and a decrease in the peak area of the paw at full stance, which may reflect a change from a flat‐footed stance to heel‐to‐toe stance. Peak paw area may be a valuable parameter for future investigations into the role of paw and toes in gait, such as toe walking, which is common in NDDs. Finally, C57 gait matured through altered variation in distinct measures. Changes to intraindividual variability in gait across time are particularly interesting, as increased variability, such as that observed in stance width, may reflect a decrease in rigidity of stance, while decreased variability, as seen in stride length and peak paw area, may reflect a fine tuning of those gait features.

In FVB mice, aspects of gait maturity were reflected in similar metrics, albeit in different directions than those seen in C57 mice. In FVB mice, the proportions of the different swing and stance phases of stride for the forelimbs are mature before P21 at 40% and 60%, respectively, while these metrics for the hind limbs mature by P27. In addition, the percent both hind limbs are in stance matured by P27. Maturation of the FVB gait was reflected by greater overlap of ipsilateral limbs, an increase in the rate of deceleration or loading of paws into stance, and an increase in peak paw area over time. It is difficult to separate peak paw area change from increased paw size, and thus, it remains uncertain how this finding relates to gait maturation. What is perhaps more clearly interpretable is the decrease in variability exhibited by FVB mice in their peak paw area over time—this may reflect more precise placement of the paw during the stance phase as the mice age.

Several important methodological considerations distinguish this study from prior work in the literature. Most studies of gait in mouse models of disease, including NDDs, are conducted in adult animals (Amende et al., 2005; Gadalla, Ross, Riddell, Bailey, & Cobb, 2014; Galante et al., 2009; Kloth et al., 2015; Schneider et al., 2012). While this is a useful endeavor to understand gait abnormalities in the mature animal, these studies provide no information about the development of such abnormalities of how they might vary over time. The few studies that have examined mouse gait at earlier time points provide valuable information at specific ages (Wozniak, Valnegri, Dearborn, Fowler, & Bonni, 2019), but represent only a snapshot of gait performance in time. In contrast, here we have characterized gait across multiple time points in a longitudinal manner and at a consistent speed, enabling the sensitivity of a repeated measures design and allowing accurate comparisons of gait across age. We further presented the data both before and after controlling for the impact of body size on each gait parameter to highlight the possibility of erroneous interpretations when body size is not considered and to help define those features that could reflect true differences in CNS circuits rather than simply changes in limb length. This baseline characterization of healthy gait development in the mouse will inform future studies of NDD models, providing insight into how and when gait irregularities arise across development and whether these represent delays in normal development, or completely distinct trajectories.

Understanding the development of mouse gait is most useful if it can inform the consequences of homologous genetic lesions seen in humans. The gait analysis system used here provides a comprehensive set of gait metrics, most of which have not been examined across development in humans and thus we cannot comment on their translational impact at this time. However, we did find key parallels in our results to that which has been observed in human gait development. Specifically, the stride of our mice at P30 was composed of 40% swing phase and 60% stance phase, mirroring the mature human stride composition (Hillman et al., 2009; Lythgo, Wilson, & Galea, 2011; Sutherland et al., 1980). These findings suggest the translational potential of this approach to interrogate the impact of mutations on gait circuitry in mouse models of human disease.

Our study had several limitations, some of which were trade‐offs intended to maximize consistency between ages tested. For example, the mice were limited to a forced speed across all four time points. As body size increases with age, the intrinsic speed or qualitative gait type at a given speed (e.g., trot versus run) may change as well. This could influence the change, or lack thereof, of some of the variables we measured. However, as speed is the greatest modulator of gait, appropriate comparisons required us to enforce a constant speed for all mice across all ages. Regardless of the variation in gait that might be revealed at different speeds, this study provides a benchmark of gait at 20 cm/s across the developmental window from P21‐P30, defining an assay that will be valuable as we begin to study how genetic and environmental disruptions of neurodevelopment impact gait development.

Ultimately, the results of this study are a normative standard against which murine models of NDDs may be compared. Mouse models of NDDs are inherently limited due to the primary cognitive impairments in these disorders often being of processes specific to humans and only paralleled in mice. However, gait abnormalities and changes in gait development are some of the few features of NDDs that may track from murine models to human disease phenotypes, as neural control of gait has many shared neural mechanisms between mouse and human (Dominici et al., 2011; Takakusaki, Tomita, & Yano, 2008). Thus, this study provides the foundation for future phenotyping of gait in mouse models that will serve as a vital window into understanding the disruption of motor circuits in human disease.

## CONFLICT OF INTEREST

The authors declare that the research was conducted in the absence of any commercial or financial relationships that could be construed as a potential conflict of interest.

## AUTHOR CONTRIBUTIONS

Data collection was conducted by SKA, KBM, and CW. Data analysis was conducted by SEM. Manuscript writing was done by SKA, KBM, CW, and SEM. SKA, JDD, and SEM edited the manuscript and designed the study.

## Supporting information

Fig S1

Fig S2

Fig S3

Fig S4

Fig S5

Fig S6

Fig S7

Fig S8

Fig S9

Fig S10

Fig S11

Table S1

Table S2

Table S3

Data S1

## Data Availability

The datasets generated and analyzed during the current study are available from the corresponding author upon reasonable request.

## References

[brb31636-bib-0001] Amende, I. , Kale, A. , McCue, S. , Glazier, S. , Morgan, J. P. , & Hampton, T. G. (2005). Gait dynamics in mouse models of Parkinson's disease and Huntington's disease. Journal of Neuroengineering and Rehabilitation, 2(1), 20 10.1186/1743-0003-2-20 16042805 PMC1201165

[brb31636-bib-0002] Dominici, N. , Ivanenko, Y. P. , Cappellini, G. , d’Avella, A. , Mondì, V. , Cicchese, M. , … Lacquaniti, F. (2011). Locomotor primitives in newborn babies and their development. Science, 334(6058), 997–999. 10.1126/science.1210617 22096202

[brb31636-bib-0003] Gadalla, K. K. E. , Ross, P. D. , Riddell, J. S. , Bailey, M. E. S. , & Cobb, S. R. (2014). Gait analysis in a Mecp2 knockout mouse model of Rett syndrome reveals early‐onset and progressive motor deficits. PLoS One, 9(11), e112889 10.1371/journal.pone.0112889 25392929 PMC4231076

[brb31636-bib-0004] Galante, M. , Jani, H. , Vanes, L. , Daniel, H. , Fisher, E. M. C. , Tybulewicz, V. L. J. , … Morice, E. (2009). Impairments in motor coordination without major changes in cerebellar plasticity in the Tc1 mouse model of Down syndrome. Human Molecular Genetics, 18(8), 1449–1463. 10.1093/hmg/ddp055 19181682 PMC2664148

[brb31636-bib-0005] Gegenhuber, B. , & Tollkuhn, J. (2019). Signatures of sex: Sex differences in gene expression in the vertebrate brain. Wiley Interdisciplinary Reviews. Developmental Biology, 9, e348 10.1002/wdev.348 31106965 PMC6864223

[brb31636-bib-0006] Hampton, T. G. , Stasko, M. R. , Kale, A. , Amende, I. , & Costa, A. C. S. (2004). Gait dynamics in trisomic mice: Quantitative neurological traits of Down syndrome. Physiology & Behavior, 82(2–3), 381–389. 10.1016/j.physbeh.2004.04.006 15276802

[brb31636-bib-0007] Hausdorff, J. M. , Cudkowicz, M. E. , Firtion, R. , Wei, J. Y. , & Goldberger, A. L. (1998). Gait variability and basal ganglia disorders: Stride‐to‐stride variations of gait cycle timing in Parkinson’s disease and Huntington’s disease. Movement Disorders, 13(3), 428–437. 10.1002/mds.870130310 9613733

[brb31636-bib-0008] Hillman, S. J. , Stansfield, B. W. , Richardson, A. M. , & Robb, J. E. (2009). Development of temporal and distance parameters of gait in normal children. Gait & Posture, 29(1), 81–85. 10.1016/j.gaitpost.2008.06.012 18701291

[brb31636-bib-0009] Hocking, D. R. , Rinehart, N. J. , McGinley, J. L. , & Bradshaw, J. L. (2008). Gait function in adults with Williams syndrome. Experimental Brain Research, 192(4), 695 10.1007/s00221-008-1586-3 18841354

[brb31636-bib-0010] Jeste, S. S. (2011). The neurology of autism spectrum disorders. Current Opinion in Neurology, 24(2), 132–139. 10.1097/WCO.0b013e3283446450 21293268 PMC3160764

[brb31636-bib-0011] Karachi, C. , Grabli, D. , Bernard, F. A. , Tandé, D. , Wattiez, N. , Belaid, H. , … François, C. (2010). Cholinergic mesencephalic neurons are involved in gait and postural disorders in Parkinson disease. The Journal of Clinical Investigation, 120(8), 2745–2754. 10.1172/JCI42642 20628197 PMC2912198

[brb31636-bib-0012] Kindregan, D. , Gallagher, L. , & Gormley, J. (2015). Gait deviations in children with autism spectrum disorders: A review. Autism Research and Treatment, 2015, e741480 10.1155/2015/741480 PMC4398922 25922766

[brb31636-bib-0013] Kloth, A. D. , Badura, A. , Li, A. , Cherskov, A. , Connolly, S. G. , Giovannucci, A. , … Wang, S.‐S.‐H. (2015). Cerebellar associative sensory learning defects in five mouse autism models. elife, 4, e06085 10.7554/eLife.06085 26158416 PMC4512177

[brb31636-bib-0014] Kraan, C. M. , Tan, A. H. J. , & Cornish, K. M. (2017). The developmental dynamics of gait maturation with a focus on spatiotemporal measures. Gait & Posture, 51, 208–217. 10.1016/j.gaitpost.2016.10.021 27816899

[brb31636-bib-0015] Laforet, G. A. , Sapp, E. , Chase, K. , McIntyre, C. , Boyce, F. M. , Campbell, M. , … Aronin, N. (2001). Changes in cortical and striatal neurons predict behavioral and electrophysiological abnormalities in a transgenic murine model of Huntington’s disease. Journal of Neuroscience, 21(23), 9112–9123. 10.1523/JNEUROSCI.21-23-09112.2001 11717344 PMC6763893

[brb31636-bib-0016] Lythgo, N. , Wilson, C. , & Galea, M. (2011). Basic gait and symmetry measures for primary school‐aged children and young adults. II: Walking at slow, free and fast speed. Gait & Posture, 33(1), 29–35. 10.1016/j.gaitpost.2010.09.017 20971013

[brb31636-bib-0017] Mosconi, M. W. , Wang, Z. , Schmitt, L. M. , Tsai, P. , & Sweeney, J. A. (2015). The role of cerebellar circuitry alterations in the pathophysiology of autism spectrum disorders. Frontiers in Neuroscience, 9, 10.3389/fnins.2015.00296 PMC4555040 26388713

[brb31636-bib-0018] Nieuwboer, A. , Dom, R. , Weerdt, W. D. , Desloovere, K. , Fieuws, S. , & Broens‐Kaucsik, E. (2001). Abnormalities of the spatiotemporal characteristics of gait at the onset of freezing in Parkinson’s disease. Movement Disorders, 16(6), 1066–1075. 10.1002/mds.1206 11748737

[brb31636-bib-0019] Pediatric Musculoskeletal Matters International . (n.d.). Gait and Motor Milestones. Retrieved from http://www.pmmonline.org/page.aspx?id=753

[brb31636-bib-0020] Rao, A. K. , Muratori, L. , Louis, E. D. , Moskowitz, C. B. , & Marder, K. S. (2008). Spectrum of gait impairments in presymptomatic and symptomatic Huntington’s disease. Movement Disorders, 23(8), 1100–1107. 10.1002/mds.21987 18412252

[brb31636-bib-0021] Schneider, T. , Skitt, Z. , Liu, Y. , Deacon, R. M. J. , Flint, J. , Karmiloff‐Smith, A. , … Tassabehji, M. (2012). Anxious, hypoactive phenotype combined with motor deficits in Gtf2ird1 null mouse model relevant to Williams syndrome. Behavioural Brain Research, 233(2), 458–473. 10.1016/j.bbr.2012.05.014 22652393

[brb31636-bib-0022] Semple, B. D. , Blomgren, K. , Gimlin, K. , Ferriero, D. M. , & Noble‐Haeusslein, L. J. (2013). Brain development in rodents and humans: Identifying benchmarks of maturation and vulnerability to injury across species. Progress in Neurobiology, 106‐107, 1–16. 10.1016/j.pneurobio.2013.04.001 PMC3737272 23583307

[brb31636-bib-0023] Sutherland, D. H. , Olshen, R. , Cooper, L. , & Woo, S. L. (1980). The development of mature gait. The Journal of Bone and Joint Surgery, 62(3), 336–353. 7364807

[brb31636-bib-0024] Takakusaki, K. , Tomita, N. , & Yano, M. (2008). Substrates for normal gait and pathophysiology of gait disturbances with respect to the basal ganglia dysfunction. Journal of Neurology, 255(Suppl 4), 19–29. 10.1007/s00415-008-4004-7 18821082

[brb31636-bib-0025] Vonghia, L. , Leggio, L. , Ferrulli, A. , Bertini, M. , Gasbarrini, G. , & Addolorato, G. (2008). Acute alcohol intoxication. European Journal of Internal Medicine, 19(8), 561–567. 10.1016/j.ejim.2007.06.033 19046719

[brb31636-bib-0026] Wozniak, D. F. , Valnegri, P. , Dearborn, J. T. , Fowler, S. C. , & Bonni, A. (2019). Conditional knockout of UBC13 produces disturbances in gait and spontaneous locomotion and exploration in mice. Scientific Reports, 9(1), 1–14. 10.1038/s41598-019-40714-3 30867488 PMC6416404

[brb31636-bib-0027] Wren, T. A. L. , Rethlefsen, S. , & Kay, R. M. (2005). Prevalence of specific gait abnormalities in children with cerebral palsy: Influence of cerebral palsy subtype, age, and previous surgery. Journal of Pediatric Orthopaedics, 25(1), 79. 15614065 10.1097/00004694-200501000-00018

