# Supplementary figures and images for: The trajectory of gait development in mice
Source: Brain Behav. 2020 Apr 24;10(6):e01636. doi: 10.1002/brb3.1636 (PMC7303394; doi:10.1002/brb3.1636)

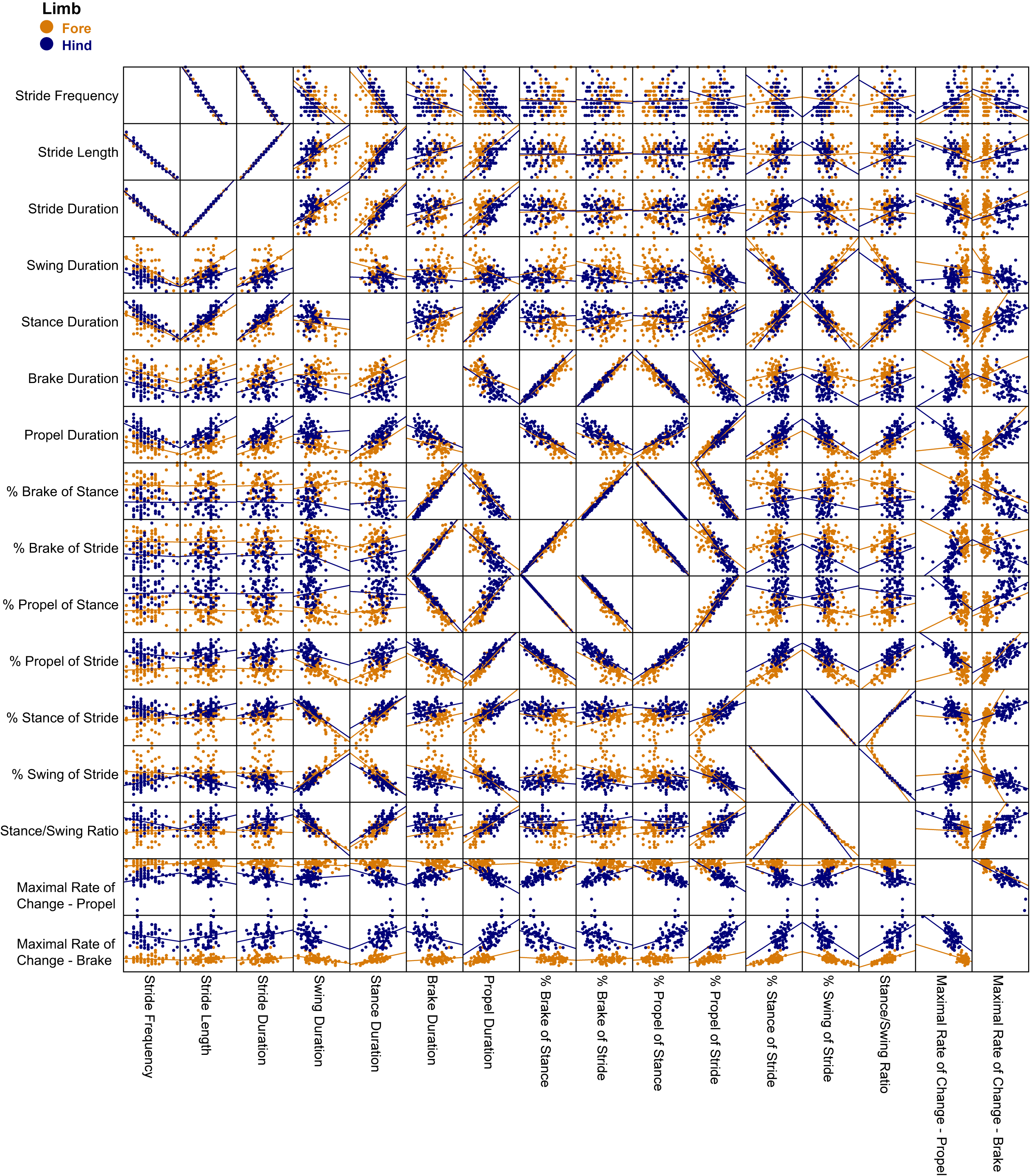

Supplement: Supplementary file 1 — Fig S1 [file BRB3-10-e01636-s001.tif]

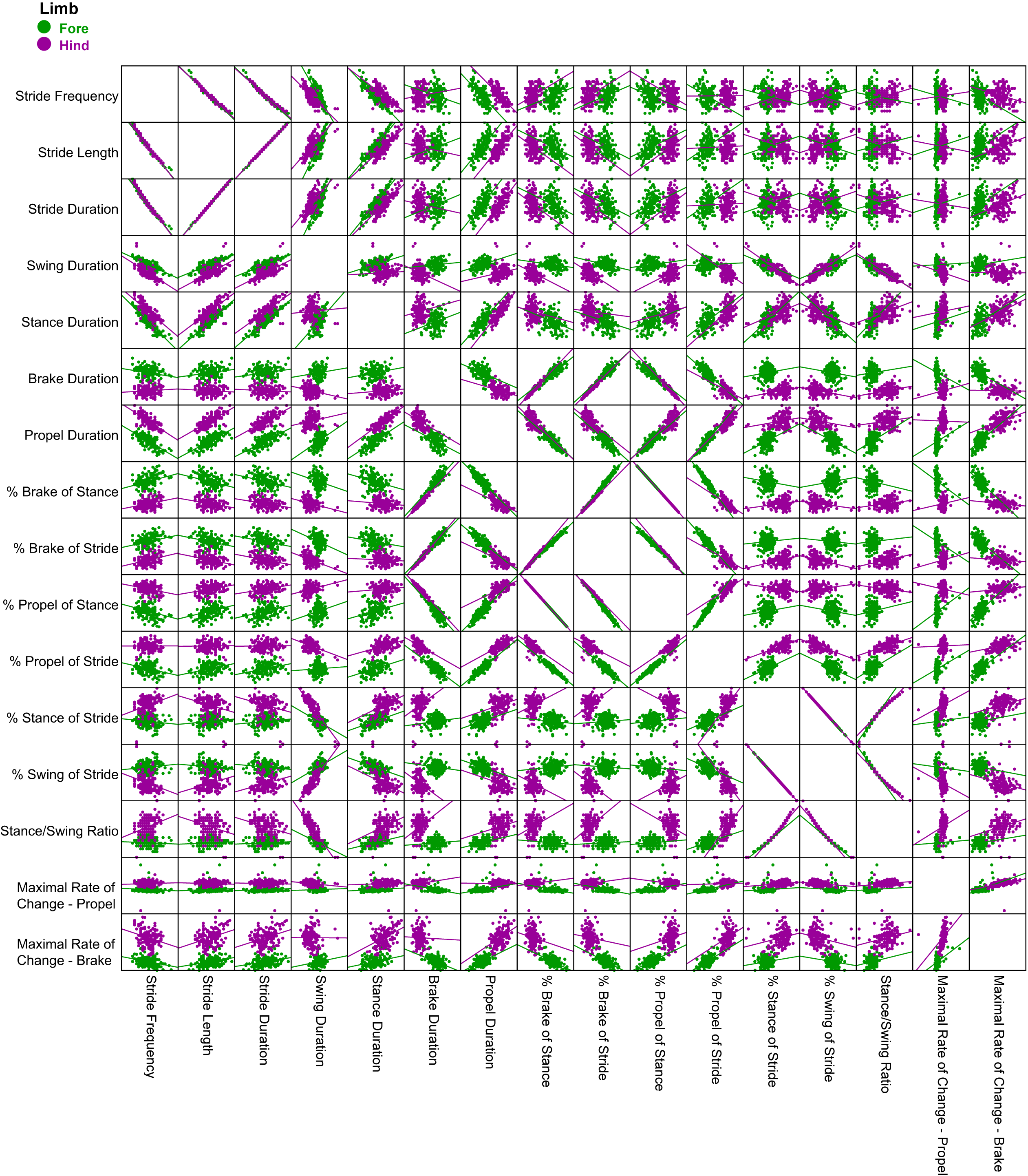

Supplement: Supplementary file 2 — Fig S2 [file BRB3-10-e01636-s002.tif]

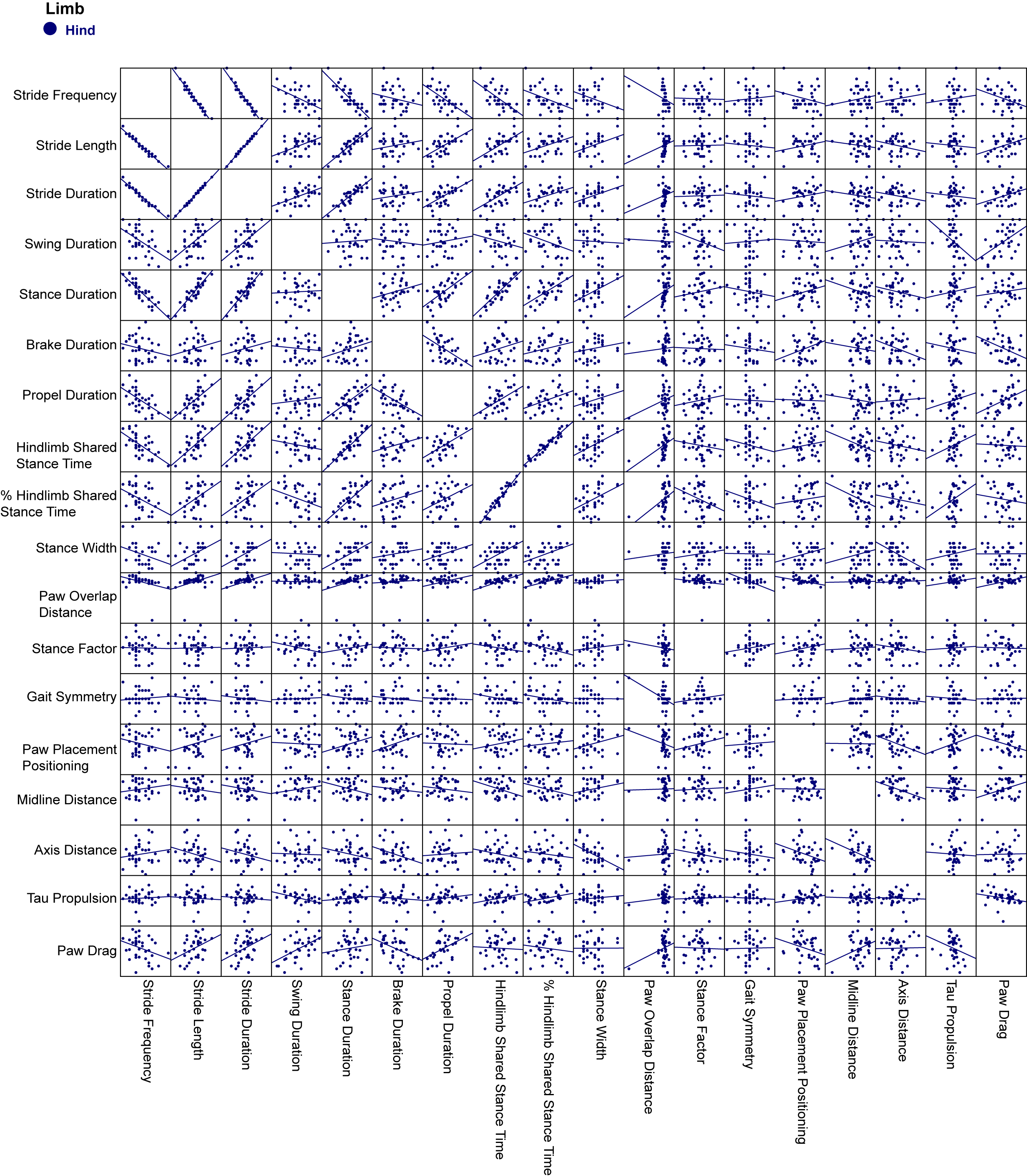

Supplement: Supplementary file 3 — Fig S3 [file BRB3-10-e01636-s003.tif]

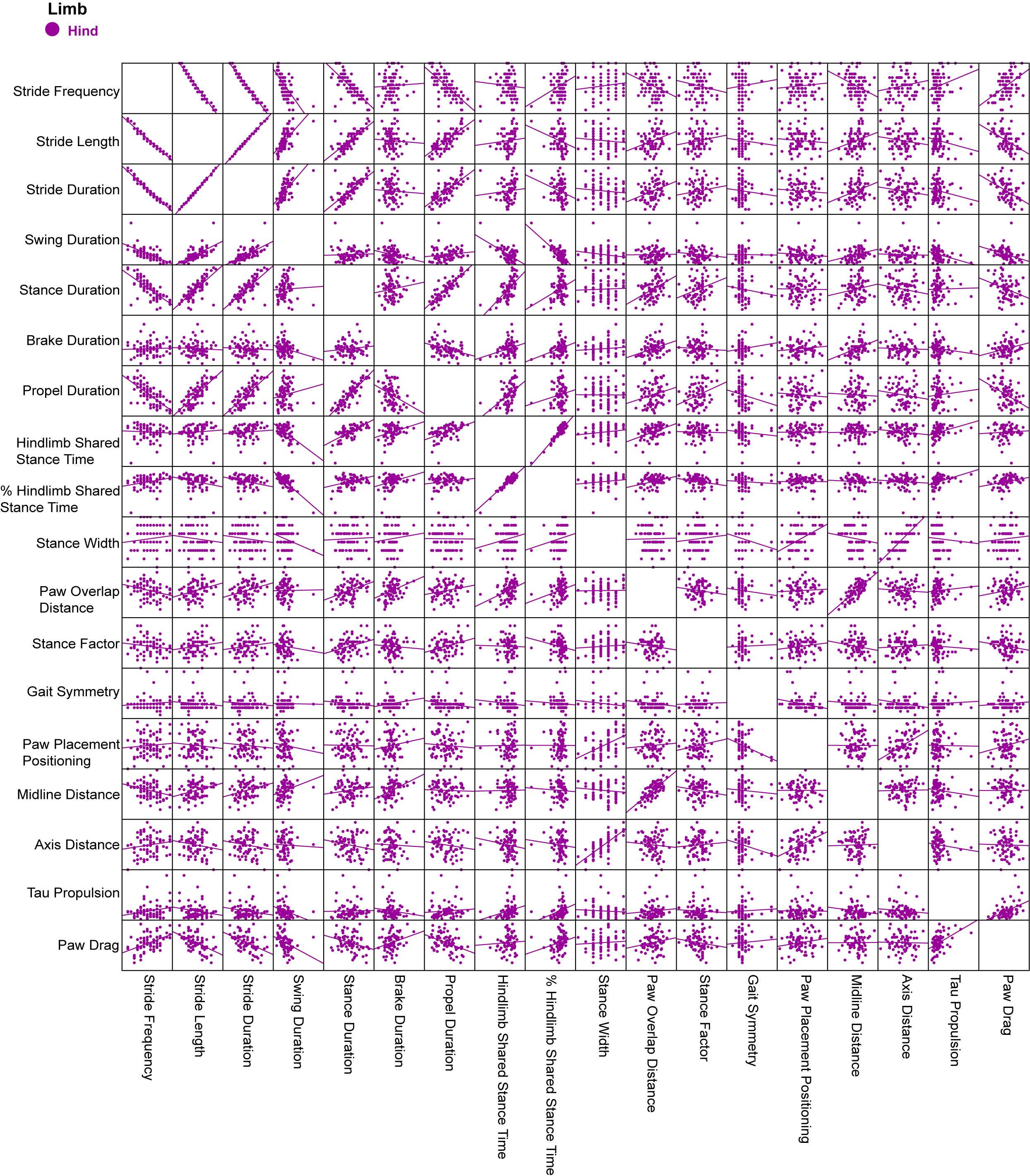

Supplement: Supplementary file 4 — Fig S4 [file BRB3-10-e01636-s004.tif]

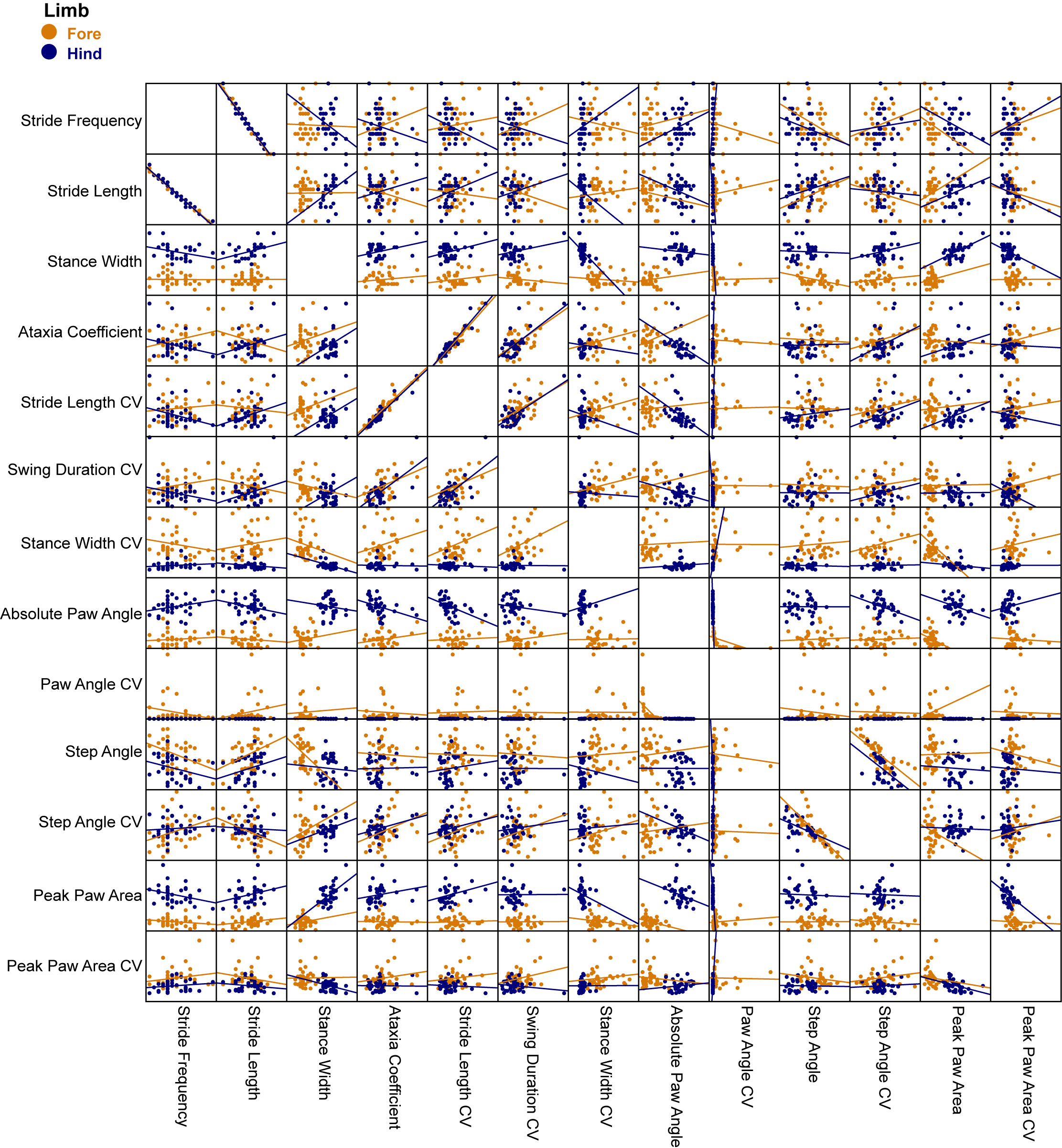

Supplement: Supplementary file 5 — Fig S5 [file BRB3-10-e01636-s005.tif]

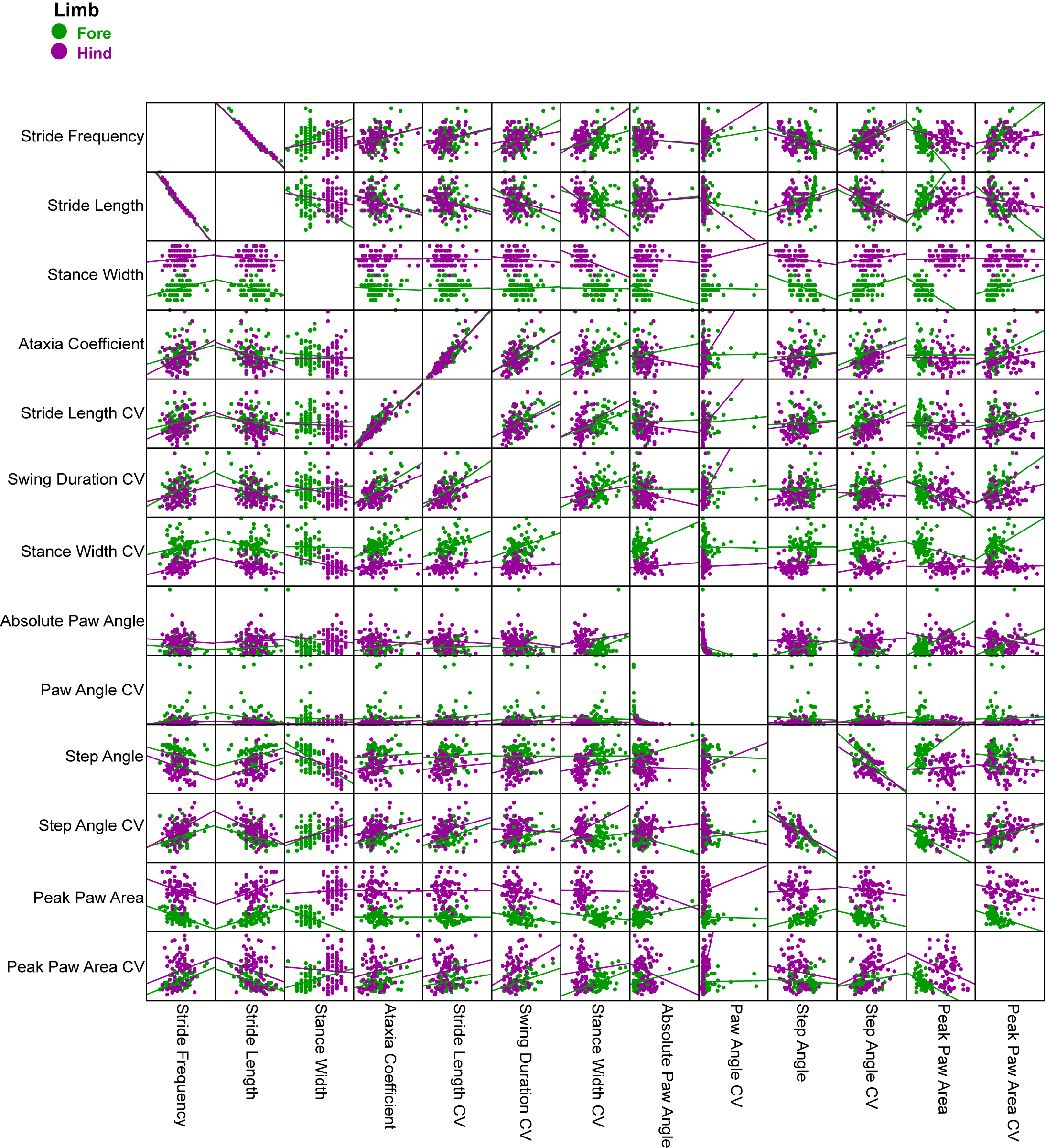

Supplement: Supplementary file 6 — Fig S6 [file BRB3-10-e01636-s006.tif]

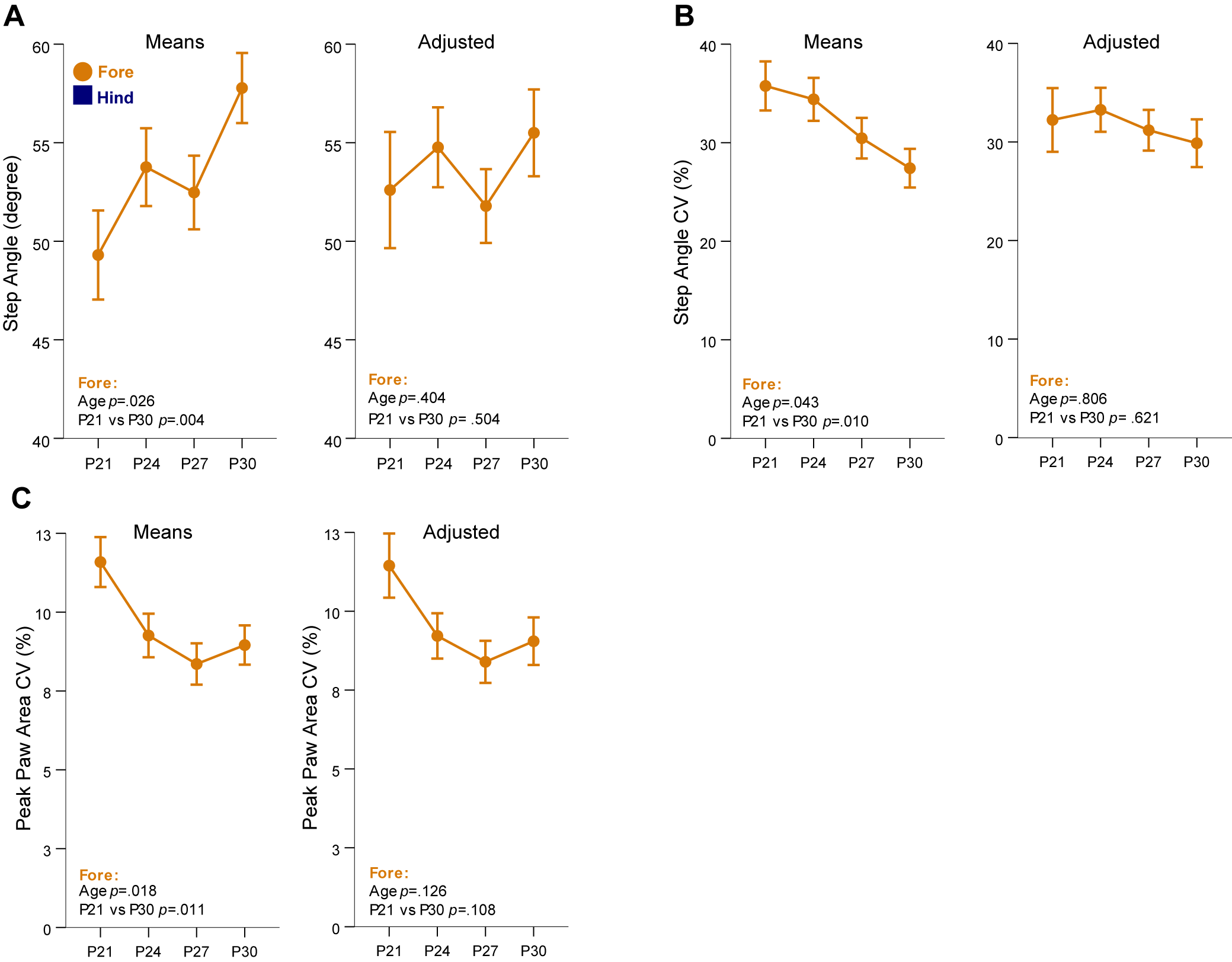

Supplement: Supplementary file 7 — Fig S7 [file BRB3-10-e01636-s007.tif]

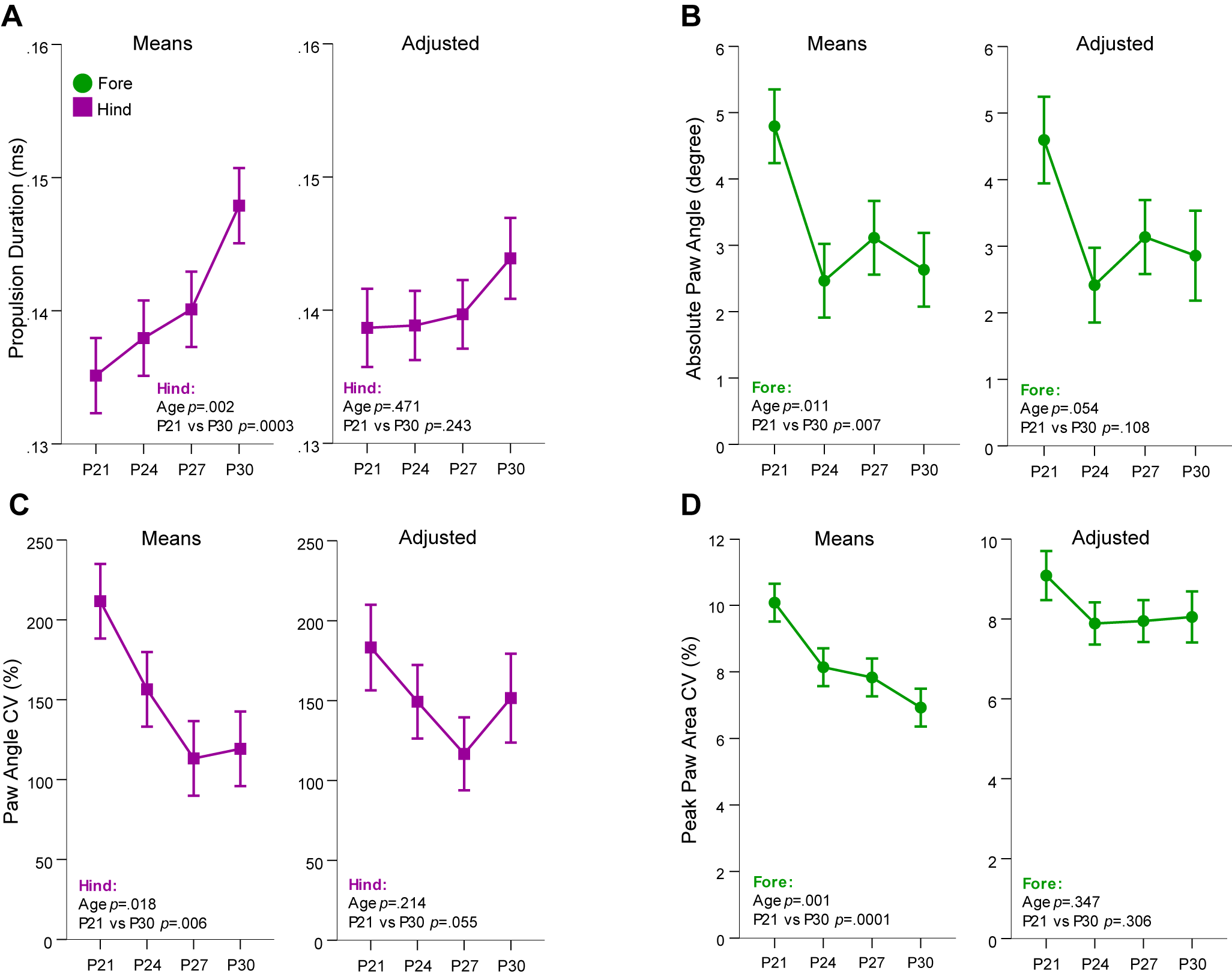

Supplement: Supplementary file 8 — Fig S8 [file BRB3-10-e01636-s008.tif]

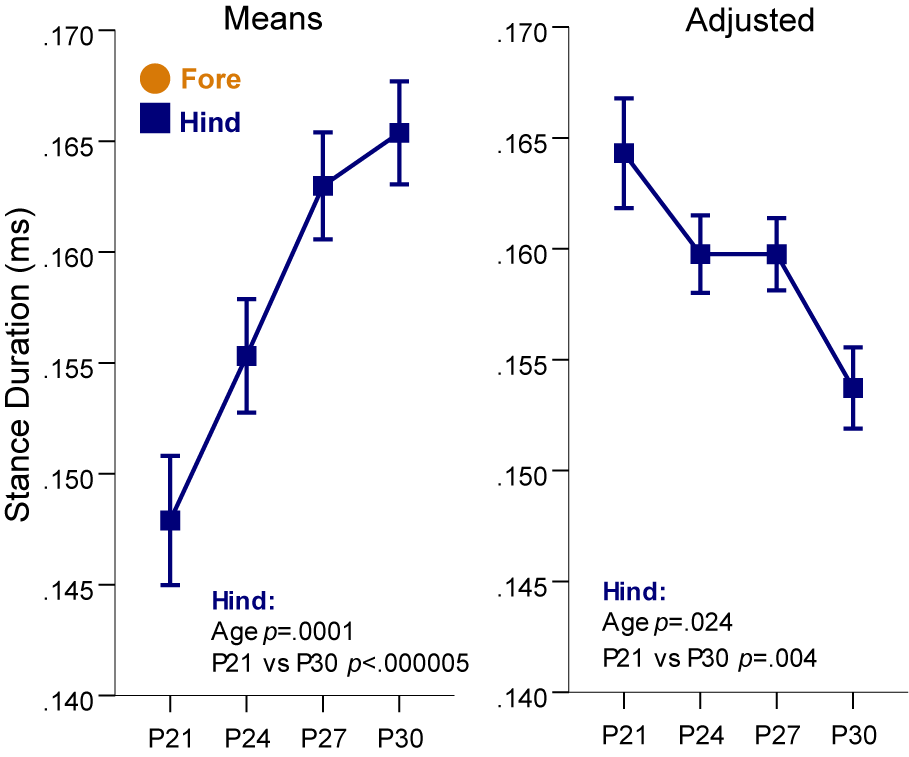

Supplement: Supplementary file 9 — Fig S9 [file BRB3-10-e01636-s009.tif]

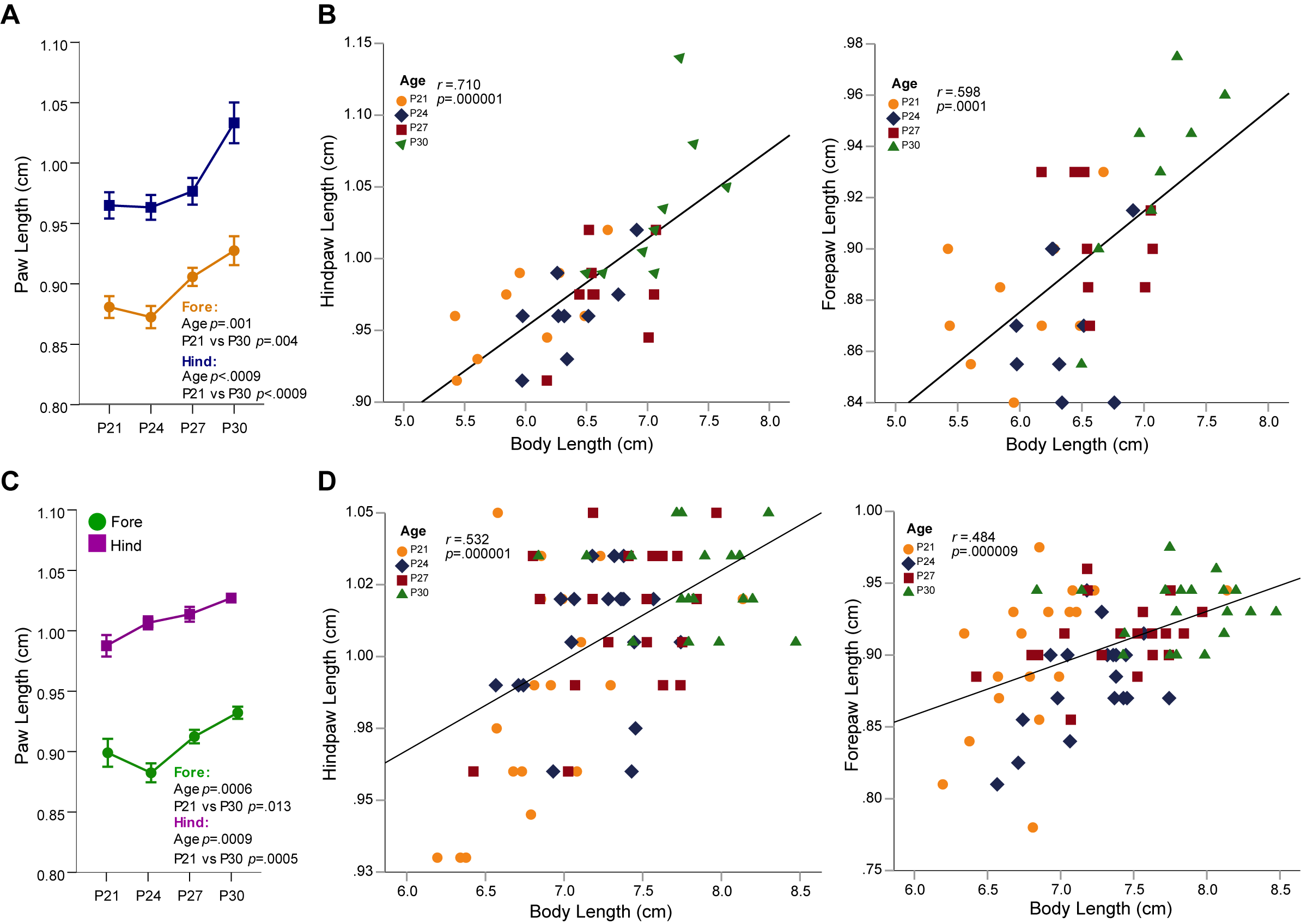

Supplement: Supplementary file 10 — Fig S10 [file BRB3-10-e01636-s010.tif]

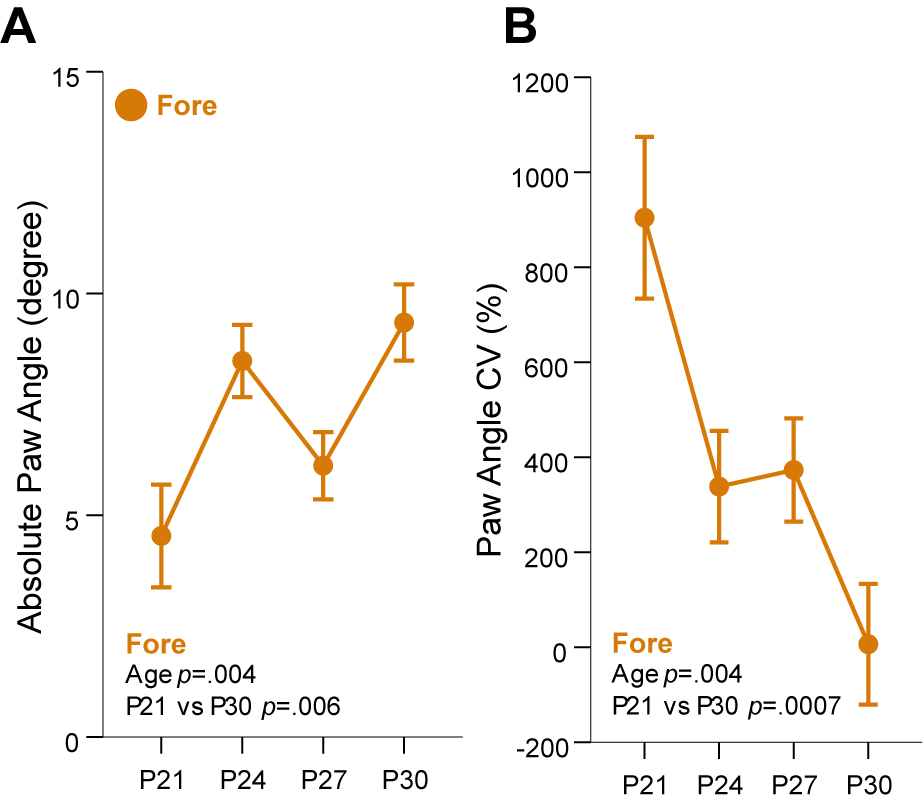

Supplement: Supplementary file 11 — Fig S11 [file BRB3-10-e01636-s011.tif]
