# Supplementary material for: The trajectory of gait development in mice
Source: Brain Behav. 2020 Apr 24;10(6):e01636. doi: 10.1002/brb3.1636 (PMC7303394; doi:10.1002/brb3.1636)
Supplement: Supplementary file 12 — Table S1 [file BRB3-10-e01636-s012.docx]

1. **Supplementary Tables**

**Supplementary Table 1. Statistical output for C57 gait data.**

| **OUTCOME** | **NORMAL** | **LMM** | **parameters** | ***t*** | **sig.** | **LMM (adjusted for body length)** | **parameters** | ***t*** | **sig.** |
| --- | --- | --- | --- | --- | --- | --- | --- | --- | --- |
| %Hindlimb Shared Stance | normal | Age F(3,44.19)=1.92, p=0.140 | P21 vs. P30 | -2.140 | 0.036 | Age F(3,38.60)=1.27, p=0.300 | P21 vs. P30 | 1.118 | 0.268 |
|  |  |  | P24 vs. P30 | -0.406 | 0.686 | Body Length F(1,49.65)=12.30, p=0.0010 | P24 vs. P30 | 1.904 | 0.061 |
|  |  |  | P27 vs. P30 | -0.405 | 0.687 |  | P27 vs. P30 | 1.252 | 0.216 |
| % Stance Fore | P30 nonnormal | Age F(3,44.79)=8.15, p=0.0002 | P21 vs. P30 | -3.099 | 0.003 | Age F(3,43.93)=8.48, p=0.0001 | P21 vs. P30 | -2.989 | 0.004 |
|  |  |  | P24 vs. P30 | 0.906 | 0.368 | Body Length F(1,68.81)=1.89, p=0.174 | P24 vs. P30 | -0.254 | 0.800 |
|  |  |  | P27 vs. P30 | 0.853 | 0.398 |  | P27 vs. P30 | 0.035 | 0.972 |
| % Stance Hind | normal | Age F(3,44.28)=2.43, p=0.077 | P21 vs. P30 | -2.438 | 0.017 | Age F(3,37.90)=1.07, p=0.373 | P21 vs. P30 | 1.068 | 0.290 |
|  |  |  | P24 vs. P30 | -1.048 | 0.299 | Body Length F(1,51.43)=12.52, p=0.0009 | P24 vs. P30 | 1.470 | 0.146 |
|  |  |  | P27 vs. P30 | 0.001 | 0.999 |  | P27 vs. P30 | 1.707 | 0.093 |
| % Swing Fore | P30 nonnormal | Age F(3,44.79)=8.15, p=0.0002 | P21 vs. P30 | 3.099 | 0.003 | Age F(3,43.93)=8.48, p=0.0001 | P21 vs. P30 | 2.989 | 0.004 |
|  |  |  | P24 vs. P30 | -0.906 | 0.368 | Body Length F(1,68.81)=1.89, p=0.174 | P24 vs. P30 | 0.254 | 0.800 |
|  |  |  | P27 vs. P30 | -0.853 | 0.398 |  | P27 vs. P30 | -0.035 | 0.972 |
| % Swing Hind | normal | Age F(3,44.28)=2.43, p=0.077 | P21 vs. P30 | 2.438 | 0.017 | Age F(3,37.90)=1.072, p=0.37 | P21 vs. P30 | -1.068 | 0.290 |
|  |  |  | P24 vs. P30 | 1.048 | 0.299 | Body Length F(1,51.43)=12.52, p=0.0009 | P24 vs. P30 | -1.470 | 0.146 |
|  |  |  | P27 vs. P30 | -0.001 | 0.999 |  | P27 vs. P30 | -1.707 | 0.093 |
| Absolute Paw Angle Fore | normal | Age F(3,51.45)=5.02, p=0.004 | P21 vs. P30 | -2.404 | 0.019 | Age F(3,43.11)=5.21, p=0.004 | P21 vs. P30 | -2.876 | 0.006 |
|  |  |  | P24 vs. P30 | 0.467 | 0.642 | Body Length F(1,42.18)=2.83, p=0.10 | P24 vs. P30 | -0.680 | 0.499 |
|  |  |  | P27 vs. P30 | -2.551 | 0.014 |  | P27 vs. P30 | -2.950 | 0.005 |
| Absolute Paw Angle Hind | normal | Age F(3,50.47)=2.45, p=0.07 | P21 vs. P30 | 1.957 | 0.054 | Age F(3,46)=1.12, p=0.35 | P21 vs. P30 | -0.365 | 0.716 |
|  |  |  | P24 vs. P30 | 0.540 | 0.591 | Body Length F(1,49.4)=5.59, p=0.022 | P24 vs. P30 | -0.974 | 0.333 |
|  |  |  | P27 vs. P30 | -0.899 | 0.373 |  | P27 vs. P30 | -1.689 | 0.097 |
| Brake Duration Fore | normal | Age F(3,48.73)=2.96, p=0.04 | P21 vs. P30 | -0.003 | 0.998 | Age F(3,47.34)=4.77, p=0.005 | P21 vs. P30 | 2.293 | 0.025 |
|  |  |  | P24 vs. P30 | -1.624 | 0.109 | Body Length F(1,65.08)=8.55, p=0.005 | P24 vs. P30 | 0.606 | 0.546 |
|  |  |  | P27 vs. P30 | 0.963 | 0.340 |  | P27 vs. P30 | 2.277 | 0.026 |
| Brake Duration Hind | normal | Age F(3,50.84)=15.62, p=2.5E-07 | P21 vs. P30 | -6.631 | 0.000 | Age F(3,42.82)=7.13, p=0.0005 | P21 vs. P30 | -3.817 | 0.0003 |
|  |  |  | P24 vs. P30 | -1.527 | 0.131 | Body Length F(1,49.21)=0.56, p=0.460 | P24 vs. P30 | -0.795 | 0.429 |
|  |  |  | P27 vs. P30 | -1.989 | 0.053 |  | P27 vs. P30 | -1.622 | 0.110 |
| Gait Symmetry | normal | Age F(3,60.82)=0.78, p=0.510 | P21 vs. P30 | 1.093 | 0.278 | Age F(3,50.57)=0.40, p=0.76 | P21 vs. P30 | 0.244 | 0.808 |
|  |  |  | P24 vs. P30 | -0.167 | 0.868 | Body Length F(1,45.56)=0.52, p=0.476 | P24 vs. P30 | -0.541 | 0.590 |
|  |  |  | P27 vs. P30 | -0.421 | 0.676 |  | P27 vs. P30 | -0.606 | 0.547 |
| Max. Rate Contact Change Fore | normal | Age F(3,49.46)=2.45, p=0.075 | P21 vs. P30 | -1.417 | 0.161 | Age F(3,46.24)=2.03, p=0.12 | P21 vs. P30 | 1.068 | 0.290 |
|  |  |  | P24 vs. P30 | -0.310 | 0.757 | Body Length F(1,56.51)=7.02, p=0.010 | P24 vs. P30 | 1.355 | 0.180 |
|  |  |  | P27 vs. P30 | 1.491 | 0.142 |  | P27 vs. P30 | 2.451 | 0.017 |
| Max. Rate Contact Change Hind | normal | Age F(3,49.07)=1.82, p=0.156 | P21 vs. P30 | 0.826 | 0.412 | Age F(3,49.14)=4.94, p=0.004 | P21 vs. P30 | 3.549 | 0.001 |
|  |  |  | P24 vs. P30 | -0.498 | 0.620 | Body Length F(1,43.91)=16.22, p=0.0002 | P24 vs. P30 | 1.716 | 0.091 |
|  |  |  | P27 vs. P30 | 1.623 | 0.111 |  | P27 vs. P30 | 2.843 | 0.006 |
| Paw Overlap Distance | P30 nonnormal | Age F(3,41.29)=1.69, p=0.183 | P21 vs. P30 | -0.233 | 0.816 | Age F(3,36.41)=4.92, p=0.006 | P21 vs. P30 | 2.850 | 0.006 |
|  |  |  | P24 vs. P30 | 1.546 | 0.127 | Body Length F(1,42.38)=16.53, p=0.0002 | P24 vs. P30 | 3.771 | 0.0004 |
|  |  |  | P27 vs. P30 | 1.509 | 0.139 |  | P27 vs. P30 | 2.716 | 0.009 |
| Paw Angle CV Fore | P21, P24, P27, P30 nonnormal | Age F(3,45.88)=3.87, p=0.015 | P21 vs. P30 | 2.952 | 0.004 | Age F(3,41.67)=5.14, p=0.004 | P21 vs. P30 | 3.595 | 0.001 |
|  |  |  | P24 vs. P30 | 0.519 | 0.606 | Body Length F(1,49.66)=4.66, p=0.036 | P24 vs. P30 | 1.778 | 0.080 |
|  |  |  | P27 vs. P30 | 1.784 | 0.081 |  | P27 vs. P30 | 2.476 | 0.016 |
| Paw Angle CV Hind | normal | Age F(3,60.39)=1.93, p=0.134 | P21 vs. P30 | 1.775 | 0.080 | Age F(3,53.27)=1.25, p=0.302 | P21 vs. P30 | 1.487 | 0.142 |
|  |  |  | P24 vs. P30 | 1.931 | 0.058 | Body Length F(1,45.36)=0.11, p=0.739 | P24 vs. P30 | 1.806 | 0.076 |
|  |  |  | P27 vs. P30 | 0.604 | 0.549 |  | P27 vs. P30 | 0.671 | 0.505 |
| Paw Placement Positioning | normal | Age F(3,47.39)=0.74, p=0.54 | P21 vs. P30 | -1.418 | 0.160 | Age F(3,46.53)=0.363, p=0.780 | P21 vs. P30 | 0.865 | 0.390 |
|  |  |  | P24 vs. P30 | -0.797 | 0.429 | Body Length F(1,63.03)=5.20, p=0.026 | P24 vs. P30 | 0.857 | 0.394 |
|  |  |  | P27 vs. P30 | -0.924 | 0.360 |  | P27 vs. P30 | 0.237 | 0.813 |
| Peak Paw Area CV Fore | normal | Age F(3,58.55)=3.63, p=0.018 | P21 vs. P30 | 2.617 | 0.011 | Age F(3,43.12)=2.01, p=0.126 | P21 vs. P30 | 1.635 | 0.108 |
|  |  |  | P24 vs. P30 | 0.327 | 0.745 | Body Length F(1,40.57)=0.05, p=0.832 | P24 vs. P30 | 0.152 | 0.880 |
|  |  |  | P27 vs. P30 | -0.646 | 0.522 |  | P27 vs. P30 | -0.670 | 0.506 |
| Peak Paw Area CV Hind | normal | Age F(3,48.55)=9.13, p=6.7E-05 | P21 vs. P30 | 4.959 | 0.000005 | Age F(3,40.34)=4.26, p=0.011 | P21 vs. P30 | 3.362 | 0.001 |
|  |  |  | P24 vs. P30 | 2.454 | 0.017 | Body Length F(1,46.70)=0.02, p=0.901 | P24 vs. P30 | 2.052 | 0.044 |
|  |  |  | P27 vs. P30 | 0.698 | 0.489 |  | P27 vs. P30 | 0.699 | 0.487 |
| Peak Paw Area Fore | normal | Age F(3,47.67)=1.27, p=0.30 | P21 vs. P30 | -0.710 | 0.480 | Age F(3,43.62)=2.26, p=0.095 | P21 vs. P30 | 2.045 | 0.045 |
|  |  |  | P24 vs. P30 | -0.452 | 0.653 | Body Length F(1,50.62)=11.38, p=0.001 | P24 vs. P30 | 1.573 | 0.121 |
|  |  |  | P27 vs. P30 | 1.186 | 0.241 |  | P27 vs. P30 | 2.398 | 0.020 |
| Peak Paw Area Hind | normal | Age F(3,45.74)=1.51, p=0.22 | P21 vs. P30 | -0.066 | 0.947 | Age F(3,45.79)=4.68, p=0.006 | P21 vs. P30 | 3.457 | 0.001 |
|  |  |  | P24 vs. P30 | -0.769 | 0.445 | Body Length F(1,41.81)=23.05, p=2.04E-05 | P24 vs. P30 | 1.901 | 0.062 |
|  |  |  | P27 vs. P30 | 1.284 | 0.206 |  | P27 vs. P30 | 2.868 | 0.006 |
| Propulsion Duration Fore | normal | Age F(3,49.42)=17.50, p=7.4E-08 | P21 vs. P30 | -6.312 | 2.2E-08 | Age F(3,47.26)=7.94, p=0.0002 | P21 vs. P30 | -2.757 | 0.008 |
|  |  |  | P24 vs. P30 | -0.910 | 0.366 | Body Length F(1,63.77)=2.56, p=0.114 | P24 vs. P30 | 0.328 | 0.744 |
|  |  |  | P27 vs. P30 | -1.005 | 0.319 |  | P27 vs. P30 | -0.174 | 0.863 |
| Propulsion Duration Hind | normal | Age F(3,47.93)=2.26, p=0.09 | P21 vs. P30 | 0.809 | 0.421 | Age F(3,47.30)=8.14, p=0.0002 | P21 vs. P30 | 4.740 | 0.00001 |
|  |  |  | P24 vs. P30 | -1.244 | 0.218 | Body Length F(1,45.13)=30.25, p=1.7E-06 | P24 vs. P30 | 1.970 | 0.053 |
|  |  |  | P27 vs. P30 | 0.859 | 0.395 |  | P27 vs. P30 | 2.622 | 0.011 |
|  |  |  |  |  |  |  |  |  |  |
| Stance Duration Fore | normal | Age F(3,46.76)=16.38, p=2.1E-07 | P21 vs. P30 | -6.450 | 1.2E-08 | Age F(3,41.75)=3.73, p=0.018 | P21 vs. P30 | -1.211 | 0.231 |
|  |  |  | P24 vs. P30 | -2.263 | 0.027 | Body Length F(1,53.19)=21.31, p=2.5E-05 | P24 vs. P30 | 0.787 | 0.434 |
|  |  |  | P27 vs. P30 | -0.177 | 0.860 |  | P27 vs. P30 | 1.647 | 0.105 |
| Stance Duration Hind | normal | Age F(3,46.45)=8.60, p=0.0001 | P21 vs. P30 | -4.818 | 8.1E-06 | Age F(3,47.55)=3.44, p=0.024 | P21 vs. P30 | 2.969 | 0.004 |
|  |  |  | P24 vs. P30 | -3.139 | 0.003 | Body Length F(1,45.45)=107.42, p=1.5E-13 | P24 vs. P30 | 2.220 | 0.030 |
|  |  |  | P27 vs. P30 | -0.879 | 0.384 |  | P27 vs. P30 | 2.554 | 0.014 |
| Stance Factor Fore | normal | Age F(3,50.18)=0.33, p=0.801 | P21 vs. P30 | -0.010 | 0.992 | Age F(3,45.10)=0.43, p=0.734 | P21 vs. P30 | -0.700 | 0.487 |
|  |  |  | P24 vs. P30 | 0.158 | 0.875 | Body Length F(1,52.82)=0.89, p=0.350 | P24 vs. P30 | -0.413 | 0.681 |
|  |  |  | P27 vs. P30 | -0.763 | 0.449 |  | P27 vs. P30 | -1.030 | 0.307 |
| Stance Factor Hind | normal | Age F(3,55.90)=2.53, p=0.07 | P21 vs. P30 | 1.379 | 0.172 | Age F(3,48.01)=2.58, p=0.065 | P21 vs. P30 | 1.159 | 0.251 |
|  |  |  | P24 vs. P30 | -0.773 | 0.442 | Body Length F(1,52.26)=0.120, p=0.742 | P24 vs. P30 | -0.438 | 0.663 |
|  |  |  | P27 vs. P30 | 1.607 | 0.114 |  | P27 vs. P30 | 1.631 | 0.108 |
| Stance Width CV Fore | normal | Age F(3,51.98)=0.83, p=0.48 | P21 vs. P30 | 0.801 | 0.426 | Age F(3,47.64)=0.87, p=0.462 | P21 vs. P30 | 0.961 | 0.340 |
|  |  |  | P24 vs. P30 | 1.167 | 0.247 | Body Length F(1,55.14)=0.330, p=0.567 | P24 vs. P30 | 1.281 | 0.205 |
|  |  |  | P27 vs. P30 | -0.231 | 0.819 |  | P27 vs. P30 | -0.003 | 0.997 |
| Stance Width CV Hind | P27 nonnormal | Age F(3,59.17)=3.69, p=0.017 | P21 vs. P30 | 0.572 | 0.569 | Age F(3,52.89)=3.35, p=0.026 | P21 vs. P30 | -0.728 | 0.469 |
|  |  |  | P24 vs. P30 | 3.192 | 0.002 | Body Length F(1,54.95)=2.19, p=0.145 | P24 vs. P30 | 1.795 | 0.077 |
|  |  |  | P27 vs. P30 | 1.490 | 0.142 |  | P27 vs. P30 | 0.931 | 0.355 |
| Stance Width Fore | normal | Age F(3,44.84)=3.06, p=0.038 | P21 vs. P30 | 1.519 | 0.133 | Age F(3,41.53)=3.57, p=0.022 | P21 vs. P30 | 1.841 | 0.070 |
|  |  |  | P24 vs. P30 | -1.209 | 0.231 | Body Length F(1,61.57)=1.27, p=0.263 | P24 vs. P30 | -0.201 | 0.842 |
|  |  |  | P27 vs. P30 | -0.784 | 0.437 |  | P27 vs. P30 | -0.180 | 0.858 |
| Stance Width Hind | normal | Age F(3,46.90)=6.04, p=0.001 | P21 vs. P30 | -0.310 | 0.758 | Age F(3,44.49)=8.58, p=0.0001 | P21 vs. P30 | 2.598 | 0.012 |
|  |  |  | P24 vs. P30 | -3.635 | 0.001 | Body Length F(1,61.09)=13.01, p=0.0006 | P24 vs. P30 | -0.732 | 0.467 |
|  |  |  | P27 vs. P30 | -1.992 | 0.052 |  | P27 vs. P30 | -0.328 | 0.744 |
| Step Angle CV Fore | normal | Age F(3,52.06)=2.91, p=0.043 | P21 vs. P30 | 2.641 | 0.010 | Age F(3,48.07)=0.327, p=0.806 | P21 vs. P30 | 0.497 | 0.621 |
|  |  |  | P24 vs. P30 | 2.424 | 0.018 | Body Length F(1,55.37)=2.74, p=0.104 | P24 vs. P30 | 0.954 | 0.343 |
|  |  |  | P27 vs. P30 | 1.168 | 0.248 |  | P27 vs. P30 | 0.470 | 0.640 |
| Step Angle CV Hind | normal | Age F(3,54.33)=0.15, p=0.93 | P21 vs. P30 | 0.401 | 0.690 | Age F(3,41.97)=0.105, p=0.956 | P21 vs. P30 | 0.319 | 0.751 |
|  |  |  | P24 vs. P30 | 0.452 | 0.653 | Body Length F(1,44.06)=0.004, p=0.949 | P24 vs. P30 | 0.407 | 0.686 |
|  |  |  | P27 vs. P30 | -0.100 | 0.920 |  | P27 vs. P30 | -0.076 | 0.939 |
| Step Angle Fore | normal | Age F(3,52.34)=3.34, p=0.026 | P21 vs. P30 | -2.952 | 0.004 | Age F(3,48.04)=0.993, p=0.404 | P21 vs. P30 | -0.672 | 0.504 |
|  |  |  | P24 vs. P30 | -1.526 | 0.132 | Body Length F(1,55.61)=2.89, p=0.095 | P24 vs. P30 | -0.228 | 0.820 |
|  |  |  | P27 vs. P30 | -2.197 | 0.033 |  | P27 vs. P30 | -1.464 | 0.148 |
| Step Angle Hind | normal | Age F(3,56.09)=1.14, p=0.34 | P21 vs. P30 | -1.602 | 0.114 | Age F(3,47.17)=0.87, p=0.464 | P21 vs. P30 | -1.148 | 0.255 |
|  |  |  | P24 vs. P30 | 0.110 | 0.912 | Body Length F(1,50.23)=0.011, p=0.916 | P24 vs. P30 | 0.030 | 0.976 |
|  |  |  | P27 vs. P30 | -0.590 | 0.558 |  | P27 vs. P30 | -0.593 | 0.556 |
| Stride Frequency Fore | normal | Age F(3,49.42)=7.26, p=0.0004 | P21 vs. P30 | 4.360 | 4.4E-05 | Age F(3,45.34)=1.004, p=0.400 | P21 vs. P30 | -1.476 | 0.145 |
|  |  |  | P24 vs. P30 | 2.847 | 0.006 | Body Length F(1,50.29)=46.51, p=1.1E-08 | P24 vs. P30 | -1.075 | 0.286 |
|  |  |  | P27 vs. P30 | 0.771 | 0.444 |  | P27 vs. P30 | -1.517 | 0.135 |
| Stride Frequency Hind | normal | Age F(3,50.22)=6.32, p=0.001 | P21 vs. P30 | 4.033 | 0.0001 | Age F(3,45.78)=0.997, p=0.403 | P21 vs. P30 | -1.661 | 0.102 |
|  |  |  | P24 vs. P30 | 2.785 | 0.007 | Body Length F(1,45.90)=47.43, p=1.4E-08 | P24 vs. P30 | -0.956 | 0.343 |
|  |  |  | P27 vs. P30 | 0.862 | 0.393 |  | P27 vs. P30 | -1.179 | 0.244 |
| Stride Length CV Fore | normal | Age F(3,57.24)=1.74, p=0.169 | P21 vs. P30 | 1.650 | 0.103 | Age F(3,48.07)=1.40, p=0.254 | P21 vs. P30 | 1.534 | 0.130 |
|  |  |  | P24 vs. P30 | 2.013 | 0.048 | Body Length F(1,49.80)=0.33, p=0.570 | P24 vs. P30 | 1.981 | 0.052 |
|  |  |  | P27 vs. P30 | 0.637 | 0.527 |  | P27 vs. P30 | 0.789 | 0.434 |
| Stride Length CV Hind | normal | Age F(3,45.95)=1.64, p=0.194 | P21 vs. P30 | -0.774 | 0.441 | Age F(3,42.26)=3.13, p=0.035 | P21 vs. P30 | 1.830 | 0.072 |
|  |  |  | P24 vs. P30 | 1.337 | 0.186 | Body Length F(1,54.39)=9.34, p=0.003 | P24 vs. P30 | 3.004 | 0.004 |
|  |  |  | P27 vs. P30 | 0.816 | 0.419 |  | P27 vs. P30 | 1.985 | 0.052 |
| Stride Length Fore | normal | Age F(3,49.58)=8.02, p=0.0002 | P21 vs. P30 | -4.458 | 0.00003 | Age F(3,44.89)=1.16, p=0.334 | P21 vs. P30 | 1.408 | 0.164 |
|  |  |  | P24 vs. P30 | -2.963 | 0.004 | Body Length F(1,48.47)=48.38, p=8.2E-09 | P24 vs. P30 | 0.950 | 0.346 |
|  |  |  | P27 vs. P30 | -0.558 | 0.580 |  | P27 vs. P30 | 1.717 | 0.092 |
| Stride Length Hind | normal | Age F(3,50.57)=6.66, p=0.0007 | P21 vs. P30 | -4.090 | 0.0001 | Age F(3,46.64)=0.994, p=0.404 | P21 vs. P30 | 1.604 | 0.114 |
|  |  |  | P24 vs. P30 | -3.040 | 0.003 | Body Length F(1,46.46)=47.17, p=1.46E-08 | P24 vs. P30 | 0.690 | 0.493 |
|  |  |  | P27 vs. P30 | -0.948 | 0.348 |  | P27 vs. P30 | 1.085 | 0.283 |
| Swing Duration CV Fore | normal | Age F(3,61.32)=1.28, p=0.29 | P21 vs. P30 | 0.527 | 0.600 | Age F(3,49.61)=1.09, p=0.361 | P21 vs. P30 | 0.027 | 0.978 |
|  |  |  | P24 vs. P30 | 1.837 | 0.070 | Body Length F(1,46.37)=0.212, p=0.648 | P24 vs. P30 | 1.271 | 0.208 |
|  |  |  | P27 vs. P30 | 1.316 | 0.195 |  | P27 vs. P30 | 1.133 | 0.262 |
| Swing Duration CV Hind | P30 nonnormal | Age F(3,45.40)=0.17, p=0.91 | P21 vs. P30 | -0.497 | 0.621 | Age F(3,43.70)=0.48, p=0.700 | P21 vs. P30 | 1.024 | 0.310 |
|  |  |  | P24 vs. P30 | -0.469 | 0.640 | Body Length F(1,56.28)=3.05, p=0.086 | P24 vs. P30 | 0.704 | 0.484 |
|  |  |  | P27 vs. P30 | -0.680 | 0.500 |  | P27 vs. P30 | 0.062 | 0.951 |
| Swing Duration Fore | normal | Age F(3,44.57)=2.24, p=0.10 | P21 vs. P30 | -0.377 | 0.707 | Age F(3,42.33)=5.46, p=0.003 | P21 vs. P30 | 2.967 | 0.004 |
|  |  |  | P24 vs. P30 | -2.271 | 0.027 | Body Length F(1,66.75)=17.00, p=0.0001 | P24 vs. P30 | 0.832 | 0.408 |
|  |  |  | P27 vs. P30 | -1.001 | 0.322 |  | P27 vs. P30 | 1.018 | 0.313 |
| Swing Duration Hind | normal | Age F(3,47.86)=0.80, p=0.50 | P21 vs. P30 | -1.095 | 0.277 | Age F(3,38.42)=0.265, p=0.850 | P21 vs. P30 | 0.003 | 0.998 |
|  |  |  | P24 vs. P30 | -1.469 | 0.146 | Body Length F(1,48.81)=0.92, p=0.342 | P24 vs. P30 | -0.581 | 0.563 |
|  |  |  | P27 vs. P30 | -0.952 | 0.347 |  | P27 vs. P30 | -0.506 | 0.615 |
