# Supplementary material for: The trajectory of gait development in mice
Source: Brain Behav. 2020 Apr 24;10(6):e01636. doi: 10.1002/brb3.1636 (PMC7303394; doi:10.1002/brb3.1636)
Supplement: Supplementary file 13 — Table S2 [file BRB3-10-e01636-s013.docx]

**Supplementary Table 2. Statistical output for FVB gait data.**

| **OUTCOME** | **NORMAL** | **LMM** | **estimates** | ***t*** | **sig.** | **LMM (adjusted for body length)** | **estimates** | ***t*** | **sig.** |
| --- | --- | --- | --- | --- | --- | --- | --- | --- | --- |
| %Hindlimb Shared Stance | normal | Age F(3,48.51)=7.58, p=0.0003 | P21 vs. P30 | -4.421 | 0.00003 | Age F(3,48.981)=7.219 p=.0004 | P21 vs. P30 | -4.152 | 0.00009 |
|  |  |  | P24 vs. P30 | -2.772 | 0.007 | Body Length F(1,75.261)=1.732, p=.192 | P24 vs. P30 | -3.070 | 0.003 |
|  |  |  | P27 vs. P30 | -0.573 | 0.569 |  | P27 vs. P30 | -1.191 | 0.238 |
| % Stance Fore | normal | Age F(3,52.07)=2.03, p=0.121 | P21 vs. P30 | -1.728 | 0.088 | Age F(3,51.440)=1.352 p=.267 | P21 vs. P30 | 0.508 | 0.613 |
|  |  |  | P24 vs. P30 | 0.335 | 0.739 | Body Length F(1,64.023)=7.224, p=.009 | P24 vs. P30 | 1.708 | 0.092 |
|  |  |  | P27 vs. P30 | -0.473 | 0.638 |  | P27 vs. P30 | 0.751 | 0.456 |
| % Stance Hind | normal | Age F(3,50.01)=9.93, p=3.04E-05 | P21 vs. P30 | -4.571 | 0.00002 | Age F(3,50.437)=10.498 p=.00002 | P21 vs. P30 | -4.520 | 0.00002 |
|  |  |  | P24 vs. P30 | -2.698 | 0.009 | Body Length F(1,74.647)=2.737, p=.102 | P24 vs. P30 | -3.212 | 0.002 |
|  |  |  | P27 vs. P30 | 0.357 | 0.722 |  | P27 vs. P30 | -0.565 | 0.574 |
| % Swing Fore | normal | Age F(3,52.07)=2.03, p=0.121 | P21 vs. P30 | 1.728 | 0.088 | Age F(3,51.440)=1.352 p=.267 | P21 vs. P30 | -0.508 | 0.613 |
|  |  |  | P24 vs. P30 | -0.335 | 0.739 | Body Length F(1,64.023)=7.224, p=.009 | P24 vs. P30 | -1.708 | 0.092 |
|  |  |  | P27 vs. P30 | 0.473 | 0.638 |  | P27 vs. P30 | -0.751 | 0.456 |
| % Swing Hind | normal | Age F(3,50.0)=9.93, p=3.0E-05 | P21 vs. P30 | 4.571 | 0.00002 | Age F(3,50.437)=10.498 p=.00002 | P21 vs. P30 | 4.520 | 0.00002 |
|  |  |  | P24 vs. P30 | 2.698 | 0.009 | Body Length F(1,74.647)=2.737, p=.102 | P24 vs. P30 | 3.212 | 0.002 |
|  |  |  | P27 vs. P30 | -0.357 | 0.722 |  | P27 vs. P30 | 0.565 | 0.574 |
| Absolute Paw Angle Fore | P21 nonnormal | Age F(3,42.23)=4.18, p=0.011 | P21 vs. P30 | 2.760 | 0.007 | Age F(3,42.074)=2.751 p=.054 | P21 vs. P30 | 1.627 | 0.108 |
|  |  | Friedman's Test: | P24 vs. P30 | -0.214 | 0.831 | Body Length F(1,56.325)=0.343, p=.560 | P24 vs. P30 | -0.489 | 0.626 |
|  |  | χ2(3,N=19)=5.286, p=.152 | P27 vs. P30 | 0.672 | 0.506 |  | P27 vs. P30 | 0.351 | 0.727 |
| Absolute Paw Angle Hind | normal | Age F(3,52.72)=0.67, p=0.58 | P21 vs. P30 | 1.288 | 0.202 | Age F(3,53.529)=1.557 p=.211 | P21 vs. P30 | 2.070 | 0.042 |
|  |  |  | P24 vs. P30 | 0.941 | 0.350 | Body Length F(167.102)=2.662, p=.107 | P24 vs. P30 | 1.677 | 0.098 |
|  |  |  | P27 vs. P30 | 1.116 | 0.270 |  | P27 vs. P30 | 1.757 | 0.084 |
| Brake Duration Fore | normal | Age F(3,52.29)=0.89, p=0.45 | P21 vs. P30 | -1.266 | 0.209 | Age F(3,52.673)=0.496 p=.687 | P21 vs. P30 | -0.016 | 0.987 |
|  |  | Friedman's Test: | P24 vs. P30 | -0.472 | 0.638 | Body Length F(1,74.065)=1.745, p=.191 | P24 vs. P30 | 0.321 | 0.749 |
|  |  | χ2(3,N=19)=6.206, p=.102 | P27 vs. P30 | 0.321 | 0.749 |  | P27 vs. P30 | 0.934 | 0.354 |
| Brake Duration Hind | normal | Age F(3,52.26)=4.56, p=0.007 | P21 vs. P30 | -3.386 | 0.001 | Age F(3,52.177)=2.801 p=.049 | P21 vs. P30 | -2.404 | 0.019 |
|  |  |  | P24 vs. P30 | -0.831 | 0.408 | Body Length F(1,65.993)=0.009, p=.924 | P24 vs. P30 | -0.656 | 0.514 |
|  |  |  | P27 vs. P30 | -1.177 | 0.245 |  | P27 vs. P30 | -1.010 | 0.316 |
| Gait Symmetry | P21, P24, P27, nonnormal | Age F(3,58.11)=0.577, p=0.632 | P21 vs. P30 | 0.862 | 0.391 | Age F(3,58.383)=0.381 p=.767 | P21 vs. P30 | -0.275 | 0.784 |
|  |  | Friedman's Test: | P24 vs. P30 | 0.862 | 0.391 | Body Length F(1,59.600)=1.976, p=.165 | P24 vs. P30 | 0.058 | 0.954 |
|  |  | χ2(3,N=19)=2.692, p=.442 | P27 vs. P30 | 1.277 | 0.208 |  | P27 vs. P30 | 0.657 | 0.513 |
| Max. Rate Contact Change Fore | normal | Age F(3,48.74)=8.45, p=0.0001 | P21 vs. P30 | -2.513 | 0.014 | Age F(3,48.024)=8.273 p=.0002 | P21 vs. P30 | -2.462 | 0.016 |
|  |  |  | P24 vs. P30 | -3.877 | 0.0002 | Body Length F(1,73.296)=0.823, p=.367 | P24 vs. P30 | -3.760 | 0.0003 |
|  |  |  | P27 vs. P30 | -4.896 | 0.00001 |  | P27 vs. P30 | -4.723 | 0.00002 |
| Max. Rate Contact Change Hind | normal | Age F(3,47.67)=10.09, p=3.0E-05 | P21 vs. P30 | -5.121 | 2.25E-06 | Age F(3,48.107)=10.984 p=.00001 | P21 vs. P30 | -5.344 | 9.47E-07 |
|  |  |  | P24 vs. P30 | -5.002 | 4.03E-06 | Body Length F(1,72.083)=4.890, p=.030 | P24 vs. P30 | -5.591 | 4.21E-07 |
|  |  |  | P27 vs. P30 | -3.606 | 0.0007 |  | P27 vs. P30 | -4.489 | 0.00004 |
| Paw Angle CV Fore | P21, P24, P30 nonnormal | Age F(3,41.66)=2.56, p=0.07 | P21 vs. P30 | -2.317 | 0.023 | Age F(3,42.031)=2.046 p=.122 | P21 vs. P30 | -1.953 | 0.055 |
|  |  | Friedman's Test: | P24 vs. P30 | -0.976 | 0.332 | Body Length F(1,57.606)=0.142, p=.707 | P24 vs. P30 | -1.030 | 0.306 |
|  |  | χ2(3,N=19)=7.926, p=.048 | P27 vs. P30 | -2.116 | 0.041 |  | P27 vs. P30 | -2.068 | 0.044 |
| Paw Angle CV Hind | P24, P30 nonnormal | Age F(3,51.84)=3.67, p=0.018 | P21 vs. P30 | 2.833 | 0.006 | Age F(3,52.626)=1.543 p=.214 | P21 vs. P30 | 0.724 | 0.472 |
|  |  | Friedman's Test: | P24 vs. P30 | 1.179 | 0.242 | Body Length F(1,69.880)=4.084, p=.047 | P24 vs. P30 | -0.063 | 0.950 |
|  |  | χ2(3,N=19)=5.716, p=.126 | P27 vs. P30 | -0.217 | 0.829 |  | P27 vs. P30 | -1.142 | 0.258 |
| Paw Overlap Distance | normal | Age F(3,55.98)=33.05, p=2.0E-12 | P21 vs. P30 | -9.557 | 1.16E-14 | Age F(3,54.359)=12.790 p=1.9E-06 | P21 vs. P30 | -5.079 | 2.68E-06 |
|  |  |  | P24 vs. P30 | -5.105 | 2.69E-06 | Body Length F(1,73.109)=13.448, p=.0005 | P24 vs. P30 | -2.534 | 0.013 |
|  |  |  | P27 vs. P30 | -3.383 | 0.001 |  | P27 vs. P30 | -1.141 | 0.258 |
| Paw Placement Positioning | normal | Age F(3,52.59)=0.87, p=0.463 | P21 vs. P30 | -1.062 | 0.291 | Age F(3,52.296)=0.416 p=.742 | P21 vs. P30 | 0.018 | 0.986 |
|  |  |  | P24 vs. P30 | -1.219 | 0.227 | Body Length F(1,59.145)=1.449, p=.233 | P24 vs. P30 | -0.440 | 0.661 |
|  |  |  | P27 vs. P30 | 0.036 | 0.971 |  | P27 vs. P30 | 0.529 | 0.599 |
| Peak Paw Area CV Fore | normal | Age F(3,52.12)=6.06, p=0.001 | P21 vs. P30 | 4.040 | 0.0001 | Age F(3,49.542)=1.128 p=.347 | P21 vs. P30 | 1.030 | 0.306 |
|  |  |  | P24 vs. P30 | 1.635 | 0.106 | Body Length F(1,68.060)=9.369, p=.003 | P24 vs. P30 | -0.194 | 0.847 |
|  |  |  | P27 vs. P30 | 1.436 | 0.157 |  | P27 vs. P30 | -0.143 | 0.887 |
| Peak Paw Area CV Hind | normal | Age F(3,55.29)=31.80, p=4.4E-12 | P21 vs. P30 | 9.044 | 1.33E-13 | Age F(3,54.239)=15.958 p=1.5E-07 | P21 vs. P30 | 5.344 | 1.01E-06 |
|  |  |  | P24 vs. P30 | 4.547 | 0.00002 | Body Length F(1,69.664)=4.445, p=.039 | P24 vs. P30 | 2.806 | 0.006 |
|  |  |  | P27 vs. P30 | 0.909 | 0.367 |  | P27 vs. P30 | -0.168 | 0.867 |
| Peak Paw Area Fore | normal | Age F(3,48.81)=8.80, p=0.00009 | P21 vs. P30 | -1.251 | 0.215 | Age F(3,47.398)=9.342 p=.00006 | P21 vs. P30 | -1.540 | 0.128 |
|  |  |  | P24 vs. P30 | -3.034 | 0.003 | Body Length F(1,69.947)=0.824, p=.367 | P24 vs. P30 | -3.056 | 0.003 |
|  |  |  | P27 vs. P30 | -4.880 | 0.00001 |  | P27 vs. P30 | -4.698 | 0.00002 |
| Peak Paw Area Hind | normal | Age F(3,48.69)=5.51, p=0.002 | P21 vs. P30 | -3.558 | 0.001 | Age F(3,48.118)=6.291 p=.001 | P21 vs. P30 | -3.862 | 0.0002 |
|  |  |  | P24 vs. P30 | -3.668 | 0.0005 | Body Length F(1,73.124)=3.208, p=.077 | P24 vs. P30 | -4.149 | 0.00009 |
|  |  |  | P27 vs. P30 | -3.220 | 0.002 |  | P27 vs. P30 | -3.846 | 0.0003 |
| Propulsion Duration Fore | normal | Age F(3,51.83)=5.31, p=0.003 | P21 vs. P30 | -1.631 | 0.107 | Age F(3,46.904)=3.710 p=.017 | P21 vs. P30 | 1.299 | 0.198 |
|  |  |  | P24 vs. P30 | -1.146 | 0.256 | Body Length F(1,73.391)=14.104, p=.0003 | P24 vs. P30 | 1.042 | 0.301 |
|  |  |  | P27 vs. P30 | -3.622 | 0.001 |  | P27 vs. P30 | -1.315 | 0.194 |
| Propulsion Duration Hind | normal | Age F(3,54.22)=5.86, p=0.002 | P21 vs. P30 | -3.821 | 0.0003 | Age F(3,50.093)=0.853 p=.471 | P21 vs. P30 | -1.176 | 0.243 |
|  |  |  | P24 vs. P30 | -3.356 | 0.001 | Body Length F(1,74.708)=6.166, p=.015 | P24 vs. P30 | -1.428 | 0.158 |
|  |  |  | P27 vs. P30 | -3.398 | 0.001 |  | P27 vs. P30 | -1.538 | 0.129 |
| Stance Duration Fore | normal | Age F(3,54.86)=5.25, p=0.003 | P21 vs. P30 | -2.925 | 0.005 | Age F(3,50.573)=4.218 p=.010 | P21 vs. P30 | 1.233 | 0.221 |
|  |  |  | P24 vs. P30 | -1.807 | 0.075 | Body Length F(1,74.488)=29.772, p=6.1E-07 | P24 vs. P30 | 1.330 | 0.188 |
|  |  |  | P27 vs. P30 | -4.243 | 0.00009 |  | P27 vs. P30 | -1.173 | 0.246 |
| Stance Duration Hind | normal | Age F(3,53.20)=0.96, p=0.417 | P21 vs. P30 | -6.315 | 1.69E-08 | Age F(3,49.505)=2.862 p=.046 | P21 vs. P30 | -2.644 | 0.010 |
|  |  |  | P24 vs. P30 | -4.397 | 0.00004 | Body Length F(1,74.508)=10.501, p=.002 | P24 vs. P30 | -1.947 | 0.055 |
|  |  |  | P27 vs. P30 | -4.857 | 0.00001 |  | P27 vs. P30 | -2.420 | 0.019 |
| Stance Factor Fore | normal | Age F(3,53.72)=8.14, p=0.0001 | P21 vs. P30 | 1.796 | 0.077 | Age F(3,54.845)=4.700 p=.005 | P21 vs. P30 | 0.868 | 0.389 |
|  |  |  | P24 vs. P30 | -1.315 | 0.192 | Body Length F(1,65.043)=0.438, p=.511 | P24 vs. P30 | -1.471 | 0.146 |
|  |  |  | P27 vs. P30 | -1.792 | 0.079 |  | P27 vs. P30 | -1.908 | 0.061 |
| Stance Factor Hind | normal | Age F(3,54.13)=15.43, p=2.3E-07 | P21 vs. P30 | 1.472 | 0.145 | Age F(3,53.181)=1.486 p=.229 | P21 vs. P30 | 1.925 | 0.058 |
|  |  |  | P24 vs. P30 | 0.304 | 0.762 | Body Length F(1,70.251)=1.516, p=.222 | P24 vs. P30 | 0.920 | 0.360 |
|  |  |  | P27 vs. P30 | 0.148 | 0.883 |  | P27 vs. P30 | 0.710 | 0.481 |
| Stance Width CV Fore | normal | Age F(3,54.47)=0.21, p=0.888 | P21 vs. P30 | -0.608 | 0.545 | Age F(3,56.373)=0.875 p=.460 | P21 vs. P30 | -1.463 | 0.148 |
|  |  |  | P24 vs. P30 | -0.708 | 0.481 | Body Length F(1,51.300)=2.402, p=.127 | P24 vs. P30 | -1.376 | 0.173 |
|  |  |  | P27 vs. P30 | -0.314 | 0.755 |  | P27 vs. P30 | -0.829 | 0.411 |
| Stance Width CV Hind | normal | Age F(3,52.76)=1.34, p=0.271 | P21 vs. P30 | 1.632 | 0.107 | Age F(3,52.867)=0.354 p=.786 | P21 vs. P30 | 0.012 | 0.990 |
|  |  |  | P24 vs. P30 | 0.879 | 0.382 | Body Length F(1,67.868)=3.085, p=.084 | P24 vs. P30 | -0.177 | 0.860 |
|  |  |  | P27 vs. P30 | 1.624 | 0.111 |  | P27 vs. P30 | 0.641 | 0.524 |
| Stance Width Fore | normal | Age F(3,51.90)=0.231, p=0.874 | P21 vs. P30 | 0.730 | 0.468 | Age F(3,47.735)=.186 p=.906 | P21 vs. P30 | -0.453 | 0.652 |
|  |  |  | P24 vs. P30 | 0.401 | 0.689 | Body Length F(1,75.985)=2.070, p=.154 | P24 vs. P30 | -0.465 | 0.643 |
|  |  |  | P27 vs. P30 | 0.000 | 1.000 |  | P27 vs. P30 | -0.739 | 0.463 |
| Stance Width Hind | normal | Age F(3,54.81)=0.990, p=0.405 | P21 vs. P30 | -0.637 | 0.526 | Age F(3,55.454)=1.147 p=.338 | P21 vs. P30 | -0.940 | 0.350 |
|  |  |  | P24 vs. P30 | -1.498 | 0.138 | Body Length F(1,75.475)=.480, p=.491 | P24 vs. P30 | -1.635 | 0.106 |
|  |  |  | P27 vs. P30 | -0.329 | 0.744 |  | P27 vs. P30 | -0.632 | 0.529 |
| Step Angle CV Fore | normal | Age F(3,51.71)=0.21, p=0.890 | P21 vs. P30 | -0.717 | 0.476 | Age F(3,52.059)=1.150 p=.338 | P21 vs. P30 | -1.826 | 0.072 |
|  |  |  | P24 vs. P30 | -0.448 | 0.656 | Body Length F(1,63.293)=3.590, p=.063 | P24 vs. P30 | -1.380 | 0.172 |
|  |  |  | P27 vs. P30 | -0.082 | 0.935 |  | P27 vs. P30 | -0.901 | 0.371 |
| Step Angle CV Hind | normal | Age F(3,52.19)=2.60, p=0.06 | P21 vs. P30 | 1.221 | 0.226 | Age F(3,51.063)=2.550 p=.066 | P21 vs. P30 | -0.360 | 0.720 |
|  |  |  | P24 vs. P30 | 1.442 | 0.154 | Body Length F(1,75.767)=3.385, p=.070 | P24 vs. P30 | 0.202 | 0.841 |
|  |  |  | P27 vs. P30 | -0.954 | 0.345 |  | P27 vs. P30 | -1.757 | 0.084 |
| Step Angle Fore | normal | Age F(3,51.25)=0.18, p=0.912 | P21 vs. P30 | -0.667 | 0.507 | Age F(3,52.446)=0.159 p=.923 | P21 vs. P30 | 0.511 | 0.611 |
|  |  |  | P24 vs. P30 | -0.114 | 0.910 | Body Length F(1,56.512)=2.310, p=.134 | P24 vs. P30 | 0.666 | 0.507 |
|  |  |  | P27 vs. P30 | -0.323 | 0.748 |  | P27 vs. P30 | 0.305 | 0.761 |
| Step Angle Hind | normal | Age F(3,53.08)=0.15, p=0.929 | P21 vs. P30 | -0.263 | 0.793 | Age F(3,58.741)=0.316 p=.814 | P21 vs. P30 | 0.767 | 0.446 |
|  |  |  | P24 vs. P30 | -0.556 | 0.580 | Body Length F(1,51.207)=2.250, p=.140 | P24 vs. P30 | 0.234 | 0.815 |
|  |  |  | P27 vs. P30 | -0.578 | 0.566 |  | P27 vs. P30 | 0.005 | 0.996 |
| Stride Frequency Fore | normal | Age F(3,54.42)=7.07, p=0.0004 | P21 vs. P30 | 2.439 | 0.017 | Age F(3,52.105)=4.336 p=.008 | P21 vs. P30 | -1.392 | 0.168 |
|  |  |  | P24 vs. P30 | 1.707 | 0.092 | Body Length F(1,75.998)=24.818, p=3.6E-06 | P24 vs. P30 | -1.174 | 0.244 |
|  |  |  | P27 vs. P30 | 4.168 | 0.0001 |  | P27 vs. P30 | 1.324 | 0.190 |
| Stride Frequency Hind | normal | Age F(3,54.09)=7.02, p=0.0005 | P21 vs. P30 | 2.330 | 0.022 | Age F(3,50.760)=4.136 p=.010 | P21 vs. P30 | -1.379 | 0.172 |
|  |  |  | P24 vs. P30 | 1.541 | 0.128 | Body Length F(1,75.608)=23.107, p=.000008 | P24 vs. P30 | -1.268 | 0.209 |
|  |  |  | P27 vs. P30 | 4.061 | 0.0002 |  | P27 vs. P30 | 1.196 | 0.236 |
| Stride Length CV Fore | normal | Age F(3,57.11)=1.73, p=0.170 | P21 vs. P30 | -1.677 | 0.098 | Age F(3,56.683)=1.671 p=.183 | P21 vs. P30 | -1.681 | 0.097 |
|  |  |  | P24 vs. P30 | -1.605 | 0.113 | Body Length F(1,61.257)=0.419, p=.520 | P24 vs. P30 | -1.716 | 0.090 |
|  |  |  | P27 vs. P30 | -0.030 | 0.976 |  | P27 vs. P30 | -0.289 | 0.773 |
| Stride Length CV Hind | normal | Age F(3,54.92)=0.478, p=0.700 | P21 vs. P30 | -0.434 | 0.665 | Age F(3,54.683)=0.788 p=.506 | P21 vs. P30 | -1.097 | 0.276 |
|  |  |  | P24 vs. P30 | 0.640 | 0.524 | Body Length F(1,61.420)=1.317, p=.256 | P24 vs. P30 | -0.033 | 0.973 |
|  |  |  | P27 vs. P30 | -0.218 | 0.828 |  | P27 vs. P30 | -0.677 | 0.501 |
| Stride Length Fore | normal | Age F(3,53.60)=7.32, p=0.0003 | P21 vs. P30 | -2.521 | 0.014 | Age F(3,50.170)=4.024 p=.012 | P21 vs. P30 | 1.176 | 0.243 |
|  |  |  | P24 vs. P30 | -2.098 | 0.040 | Body Length F(1,75.959)=22.304, p=.00001 | P24 vs. P30 | 0.708 | 0.481 |
|  |  |  | P27 vs. P30 | -4.411 | 0.00005 |  | P27 vs. P30 | -1.570 | 0.122 |
| Stride Length Hind | normal | Age F(3,53.58)=8.19, p=0.0001 | P21 vs. P30 | -2.696 | 0.009 | Age F(3,49.715)=4.110 p=.011 | P21 vs. P30 | 0.908 | 0.367 |
|  |  |  | P24 vs. P30 | -2.102 | 0.039 | Body Length F(1,75.693)=20.333, p=.00002 | P24 vs. P30 | 0.618 | 0.538 |
|  |  |  | P27 vs. P30 | -4.582 | 0.00003 |  | P27 vs. P30 | -1.752 | 0.085 |
| Swing Duration CV Fore | normal | Age F(3,51.78)=1.36, p=0.266 | P21 vs. P30 | -1.708 | 0.092 | Age F(3,51.110)=1.902 p=.141 | P21 vs. P30 | -2.117 | 0.038 |
|  |  |  | P24 vs. P30 | -0.654 | 0.515 | Body Length F(1,58.880)=1.552, p=.218 | P24 vs. P30 | -1.205 | 0.232 |
|  |  |  | P27 vs. P30 | 0.137 | 0.891 |  | P27 vs. P30 | -0.395 | 0.695 |
| Swing Duration CV Hind | normal | Age F(3,50.61)=2.77, p=0.051 | P21 vs. P30 | 2.590 | 0.012 | Age F(3,50.603)=1.306 p=.283 | P21 vs. P30 | 1.496 | 0.139 |
|  |  |  | P24 vs. P30 | 1.166 | 0.247 | Body Length F(1,58.395)=0.389, p=.535 | P24 vs. P30 | 0.683 | 0.497 |
|  |  |  | P27 vs. P30 | 0.140 | 0.889 |  | P27 vs. P30 | -0.132 | 0.895 |
| Swing Duration Fore | normal | Age F(3,52.81)=3.36, p=0.026 | P21 vs. P30 | -1.332 | 0.187 | Age F(3,51.581)=2.728 p=.053 | P21 vs. P30 | 1.247 | 0.216 |
|  |  |  | P24 vs. P30 | -1.779 | 0.079 | Body Length F(1,69.908)=11.086, p=.001 | P24 vs. P30 | 0.213 | 0.832 |
|  |  |  | P27 vs. P30 | -3.169 | 0.003 |  | P27 vs. P30 | -1.248 | 0.217 |
| Swing Duration Hind | normal | Age F(3,50.31)=5.15, p=0.003 | P21 vs. P30 | 1.740 | 0.086 | Age F(3,48.861)=8.474 p=.0001 | P21 vs. P30 | 3.616 | 0.001 |
|  |  |  | P24 vs. P30 | 0.786 | 0.435 | Body Length F(1,75.690)=10.861, p=.002 | P24 vs. P30 | 2.532 | 0.014 |
|  |  |  | P27 vs. P30 | -2.118 | 0.039 |  | P27 vs. P30 | -0.183 | 0.856 |
