# Supplementary material for: The trajectory of gait development in mice
Source: Brain Behav. 2020 Apr 24;10(6):e01636. doi: 10.1002/brb3.1636 (PMC7303394; doi:10.1002/brb3.1636)
Supplement: Supplementary file 14 — Table S3 [file BRB3-10-e01636-s014.docx]

**Supplementary Table 3. Descriptive statistics for C57 and FVB gait data.**

| **Outcome** | **Age** | **C57 Unadjusted** | | **C57 Adjusted** | | **FVB Unadjusted** | | **FVB Adjusted** | |
| --- | --- | --- | --- | --- | --- | --- | --- | --- | --- |
|  |  | **Mean** | ***SD*** | **Mean** | ***SD*** | **Mean** | ***SD*** | **Mean** | ***SD*** |
| % Hindlimb Shared Stance | P21 | 39.599 | 7.930 | 43.680 | 9.643 | 43.434 | 8.596 | 42.158 | 9.890 |
|  | P24 | 43.111 | 6.970 | 44.521 | 6.620 | 47.618 | 8.596 | 47.295 | 8.707 |
|  | P27 | 43.222 | 6.564 | 42.559 | 6.113 | 52.308 | 8.596 | 52.460 | 8.639 |
|  | P30 | 43.814 | 6.335 | 40.508 | 7.224 | 53.242 | 8.596 | 54.690 | 10.226 |
| % Stance Fore | P21 | 57.831 | 4.257 | 56.801 | 5.695 | 59.492 | 1.686 | 60.022 | 1.880 |
|  | P24 | 61.785 | 3.766 | 61.461 | 3.937 | 60.466 | 1.686 | 60.600 | 1.619 |
|  | P27 | 61.600 | 3.548 | 61.787 | 3.599 | 60.113 | 1.686 | 60.050 | 1.604 |
|  | P30 | 60.992 | 3.452 | 61.759 | 4.433 | 60.311 | 1.686 | 59.709 | 1.952 |
| % Stance Hind | P21 | 62.893 | 3.078 | 64.582 | 3.734 | 64.842 | 3.320 | 64.237 | 3.782 |
|  | P24 | 64.041 | 2.714 | 64.601 | 2.556 | 66.632 | 3.320 | 66.478 | 3.343 |
|  | P27 | 64.724 | 2.556 | 64.467 | 2.356 | 68.921 | 3.320 | 68.993 | 3.318 |
|  | P30 | 64.723 | 2.478 | 63.406 | 2.807 | 68.703 | 3.320 | 69.389 | 3.907 |
| % Swing Fore | P21 | 42.169 | 4.257 | 43.199 | 5.695 | 40.508 | 1.686 | 39.978 | 1.880 |
|  | P24 | 38.215 | 3.766 | 38.539 | 3.937 | 39.534 | 1.686 | 39.400 | 1.619 |
|  | P27 | 38.400 | 3.548 | 38.213 | 3.599 | 39.887 | 1.686 | 39.950 | 1.604 |
|  | P30 | 39.008 | 3.452 | 38.241 | 4.433 | 39.689 | 1.686 | 40.291 | 1.952 |
| % Swing Hind | P21 | 37.107 | 3.078 | 35.418 | 3.734 | 35.158 | 3.320 | 35.763 | 3.782 |
|  | P24 | 35.959 | 2.714 | 35.399 | 2.556 | 33.368 | 3.320 | 33.522 | 3.343 |
|  | P27 | 35.276 | 2.556 | 35.533 | 2.356 | 31.079 | 3.320 | 31.007 | 3.318 |
|  | P30 | 35.277 | 2.478 | 36.594 | 2.807 | 31.297 | 3.320 | 30.611 | 3.907 |
| Absolute Paw Angle Fore | P21 | 5.685 | 4.549 | 4.536 | 5.791 | 4.795 | 2.778 | 4.595 | 3.253 |
|  | P24 | 8.966 | 3.978 | 8.481 | 4.078 | 2.466 | 2.778 | 2.415 | 2.804 |
|  | P27 | 5.897 | 3.762 | 6.119 | 3.790 | 3.113 | 2.778 | 3.137 | 2.778 |
|  | P30 | 8.468 | 3.580 | 9.347 | 4.292 | 2.632 | 2.778 | 2.858 | 3.378 |
| Absolute Paw Angle Hind | P21 | 24.467 | 4.069 | 22.994 | 5.135 | 4.526 | 1.851 | 4.895 | 2.146 |
|  | P24 | 22.955 | 3.560 | 22.439 | 3.573 | 4.337 | 1.851 | 4.430 | 1.847 |
|  | P27 | 21.659 | 3.364 | 21.969 | 3.315 | 4.374 | 1.851 | 4.330 | 1.830 |
|  | P30 | 22.442 | 3.207 | 23.540 | 3.816 | 3.855 | 1.851 | 3.437 | 2.229 |
| Brake Duration Fore | P21 | 0.049 | 0.011 | 0.055 | 0.014 | 0.055 | 0.010 | 0.056 | 0.011 |
|  | P24 | 0.046 | 0.010 | 0.047 | 0.010 | 0.057 | 0.010 | 0.057 | 0.010 |
|  | P27 | 0.051 | 0.009 | 0.050 | 0.009 | 0.059 | 0.010 | 0.058 | 0.010 |
|  | P30 | 0.049 | 0.009 | 0.046 | 0.011 | 0.058 | 0.010 | 0.056 | 0.012 |
| Brake Duration Hind | P21 | 0.023 | 0.013 | 0.025 | 0.017 | 0.022 | 0.006 | 0.022 | 0.007 |
|  | P24 | 0.040 | 0.011 | 0.040 | 0.012 | 0.026 | 0.006 | 0.026 | 0.006 |
|  | P27 | 0.039 | 0.010 | 0.038 | 0.011 | 0.026 | 0.006 | 0.026 | 0.006 |
|  | P30 | 0.044 | 0.010 | 0.043 | 0.012 | 0.028 | 0.006 | 0.027 | 0.007 |
| Gait Symmetry | P21 | 1.009 | 0.029 | 1.006 | 0.036 | 1.002 | 0.022 | 0.999 | 0.025 |
|  | P24 | 1.000 | 0.025 | 0.999 | 0.026 | 1.002 | 0.022 | 1.001 | 0.022 |
|  | P27 | 0.998 | 0.024 | 0.999 | 0.024 | 1.005 | 0.022 | 1.005 | 0.021 |
|  | P30 | 1.001 | 0.022 | 1.003 | 0.027 | 0.997 | 0.022 | 1.001 | 0.026 |
| Maximal Rate of Paw Contact Change Fore | P21 | 31.023 | 9.421 | 35.140 | 11.930 | 43.887 | 9.579 | 42.929 | 11.112 |
|  | P24 | 33.726 | 8.260 | 34.911 | 8.176 | 41.475 | 9.579 | 41.232 | 9.870 |
|  | P27 | 37.128 | 7.786 | 36.300 | 7.542 | 41.463 | 9.579 | 41.577 | 9.799 |
|  | P30 | 34.384 | 7.476 | 31.385 | 8.953 | 49.956 | 9.579 | 51.043 | 11.466 |
| Maximal Rate of Paw Contact Change Hind | P21 | 90.209 | 22.890 | 103.906 | 26.867 | 88.823 | 16.074 | 84.895 | 19.036 |
|  | P24 | 82.738 | 20.023 | 86.646 | 19.128 | 91.074 | 16.074 | 90.078 | 16.988 |
|  | P27 | 93.411 | 18.926 | 91.488 | 17.792 | 99.080 | 16.074 | 99.548 | 16.871 |
|  | P30 | 85.400 | 18.033 | 76.536 | 19.898 | 110.125 | 16.074 | 114.581 | 19.622 |
| Paw Overlap Distance Hind | P21 | 2.115 | 1.146 | 2.816 | 1.351 | 1.071 | 0.384 | 1.209 | 0.405 |
|  | P24 | 2.587 | 1.004 | 2.811 | 0.937 | 1.536 | 0.384 | 1.571 | 0.361 |
|  | P27 | 2.529 | 0.947 | 2.344 | 0.868 | 1.742 | 0.384 | 1.726 | 0.359 |
|  | P30 | 2.183 | 0.907 | 1.691 | 1.006 | 1.963 | 0.384 | 1.807 | 0.417 |
| Paw Angle CV Fore | P21 | 673.906 | 665.245 | 904.115 | 851.238 | 136.512 | 295.803 | 122.773 | 347.138 |
|  | P24 | 255.219 | 582.385 | 338.322 | 586.882 | 249.486 | 295.803 | 246.002 | 299.119 |
|  | P27 | 420.303 | 549.753 | 373.145 | 542.982 | 169.963 | 295.803 | 171.600 | 296.327 |
|  | P30 | 175.676 | 525.441 | 6.461 | 635.558 | 329.741 | 295.803 | 345.327 | 360.590 |
| Paw Angle CV Hind | P21 | 26.287 | 10.288 | 26.816 | 13.028 | 211.679 | 116.804 | 183.218 | 133.856 |
|  | P24 | 26.247 | 9.002 | 26.417 | 9.339 | 156.490 | 116.804 | 149.272 | 115.227 |
|  | P27 | 23.143 | 8.504 | 23.022 | 8.689 | 113.253 | 116.804 | 116.643 | 114.143 |
|  | P30 | 21.627 | 8.112 | 21.274 | 9.650 | 119.275 | 116.804 | 151.564 | 139.072 |
| Paw Placement Positioning Hind | P21 | 0.468 | 0.144 | 0.525 | 0.186 | 0.300 | 0.082 | 0.311 | 0.094 |
|  | P24 | 0.494 | 0.126 | 0.512 | 0.127 | 0.296 | 0.082 | 0.299 | 0.081 |
|  | P27 | 0.495 | 0.119 | 0.485 | 0.116 | 0.325 | 0.082 | 0.324 | 0.081 |
|  | P30 | 0.519 | 0.115 | 0.478 | 0.142 | 0.324 | 0.082 | 0.311 | 0.098 |
| Peak Paw Area CV Fore | P21 | 11.587 | 3.953 | 11.446 | 5.089 | 10.077 | 2.839 | 9.086 | 3.075 |
|  | P24 | 9.257 | 3.457 | 9.219 | 3.598 | 8.138 | 2.839 | 7.887 | 2.646 |
|  | P27 | 8.355 | 3.269 | 8.396 | 3.345 | 7.831 | 2.839 | 7.949 | 2.621 |
|  | P30 | 8.954 | 3.111 | 9.049 | 3.770 | 6.926 | 2.839 | 8.050 | 3.194 |
| Peak Paw Area CV Hind | P21 | 8.639 | 2.331 | 8.691 | 3.079 | 19.677 | 4.264 | 18.607 | 4.822 |
|  | P24 | 7.038 | 2.039 | 7.052 | 2.131 | 14.145 | 4.264 | 13.873 | 4.150 |
|  | P27 | 6.054 | 1.928 | 6.045 | 1.974 | 9.860 | 4.264 | 9.988 | 4.111 |
|  | P30 | 5.697 | 1.835 | 5.664 | 2.294 | 8.960 | 4.264 | 10.174 | 5.010 |
| Peak Paw Area Fore | P21 | 0.458 | 0.135 | 0.530 | 0.165 | 0.688 | 0.104 | 0.678 | 0.120 |
|  | P24 | 0.468 | 0.118 | 0.488 | 0.114 | 0.651 | 0.104 | 0.649 | 0.108 |
|  | P27 | 0.514 | 0.111 | 0.500 | 0.106 | 0.634 | 0.104 | 0.635 | 0.107 |
|  | P30 | 0.482 | 0.107 | 0.431 | 0.123 | 0.720 | 0.104 | 0.731 | 0.124 |
| Peak Paw Area Hind | P21 | 1.053 | 0.230 | 1.213 | 0.261 | 1.071 | 0.185 | 1.034 | 0.218 |
|  | P24 | 1.016 | 0.201 | 1.062 | 0.186 | 1.081 | 0.185 | 1.071 | 0.194 |
|  | P27 | 1.118 | 0.190 | 1.098 | 0.173 | 1.128 | 0.185 | 1.133 | 0.193 |
|  | P30 | 1.057 | 0.182 | 0.953 | 0.194 | 1.241 | 0.185 | 1.283 | 0.225 |
|  |  |  |  |  |  |  |  |  |  |
|  |  |  |  |  |  |  |  |  |  |
|  |  |  |  |  |  |  |  |  |  |
| Propulsion Duration Fore | P21 | 0.085 | 0.014 | 0.089 | 0.018 | 0.089 | 0.015 | 0.095 | 0.015 |
|  | P24 | 0.104 | 0.012 | 0.105 | 0.012 | 0.092 | 0.015 | 0.093 | 0.013 |
|  | P27 | 0.104 | 0.011 | 0.103 | 0.011 | 0.085 | 0.015 | 0.085 | 0.013 |
|  | P30 | 0.106 | 0.011 | 0.103 | 0.014 | 0.096 | 0.015 | 0.089 | 0.016 |
| Propulsion Duration Hind | P21 | 0.125 | 0.020 | 0.140 | 0.022 | 0.135 | 0.014 | 0.139 | 0.015 |
|  | P24 | 0.115 | 0.017 | 0.120 | 0.015 | 0.138 | 0.014 | 0.139 | 0.013 |
|  | P27 | 0.124 | 0.016 | 0.121 | 0.014 | 0.140 | 0.014 | 0.140 | 0.013 |
|  | P30 | 0.121 | 0.016 | 0.110 | 0.016 | 0.148 | 0.014 | 0.144 | 0.015 |
| Stance Factor Fore | P21 | 1.000 | 0.086 | 0.987 | 0.113 | 1.018 | 0.060 | 1.014 | 0.071 |
|  | P24 | 1.004 | 0.075 | 1.000 | 0.078 | 0.966 | 0.060 | 0.965 | 0.061 |
|  | P27 | 0.987 | 0.071 | 0.990 | 0.072 | 0.960 | 0.060 | 0.961 | 0.060 |
|  | P30 | 1.001 | 0.068 | 1.010 | 0.085 | 0.988 | 0.060 | 0.993 | 0.073 |
| Stance Factor Hind | P21 | 1.033 | 0.062 | 1.037 | 0.082 | 1.021 | 0.045 | 1.027 | 0.052 |
|  | P24 | 1.000 | 0.055 | 1.001 | 0.057 | 1.006 | 0.045 | 1.008 | 0.045 |
|  | P27 | 1.034 | 0.052 | 1.033 | 0.053 | 1.004 | 0.045 | 1.003 | 0.045 |
|  | P30 | 1.011 | 0.049 | 1.009 | 0.061 | 1.002 | 0.045 | 0.994 | 0.054 |
| Stance Duration Fore | P21 | 0.134 | 0.013 | 0.144 | 0.015 | 0.144 | 0.014 | 0.151 | 0.013 |
|  | P24 | 0.149 | 0.011 | 0.152 | 0.010 | 0.148 | 0.014 | 0.150 | 0.011 |
|  | P27 | 0.155 | 0.011 | 0.153 | 0.010 | 0.144 | 0.014 | 0.143 | 0.011 |
|  | P30 | 0.156 | 0.010 | 0.149 | 0.011 | 0.154 | 0.014 | 0.146 | 0.013 |
| Stance Duration Hind | P21 | 0.148 | 0.015 | 0.164 | 0.012 | 0.157 | 0.013 | 0.161 | 0.013 |
|  | P24 | 0.155 | 0.013 | 0.160 | 0.009 | 0.164 | 0.013 | 0.165 | 0.011 |
|  | P27 | 0.163 | 0.012 | 0.160 | 0.008 | 0.166 | 0.013 | 0.165 | 0.011 |
|  | P30 | 0.165 | 0.012 | 0.154 | 0.009 | 0.175 | 0.013 | 0.171 | 0.013 |
| Stance Width CV Fore | P21 | 19.171 | 9.484 | 20.129 | 12.580 | 14.014 | 3.791 | 13.371 | 4.287 |
|  | P24 | 19.819 | 8.298 | 20.111 | 8.661 | 13.911 | 3.791 | 13.748 | 3.789 |
|  | P27 | 16.772 | 7.841 | 16.573 | 8.008 | 14.313 | 3.791 | 14.389 | 3.761 |
|  | P30 | 17.239 | 7.475 | 16.580 | 9.402 | 14.666 | 3.791 | 15.395 | 4.428 |
| Stance Width CV Hind | P21 | 6.767 | 2.686 | 6.092 | 3.460 | 7.534 | 2.186 | 7.072 | 2.498 |
|  | P24 | 8.392 | 2.349 | 8.192 | 2.413 | 7.058 | 2.186 | 6.941 | 2.150 |
|  | P27 | 7.274 | 2.221 | 7.413 | 2.239 | 7.382 | 2.186 | 7.437 | 2.130 |
|  | P30 | 6.377 | 2.113 | 6.825 | 2.570 | 6.538 | 2.186 | 7.062 | 2.596 |
| Stance Width Fore | P21 | 2.120 | 0.263 | 2.174 | 0.353 | 1.458 | 0.170 | 1.431 | 0.187 |
|  | P24 | 1.950 | 0.231 | 1.966 | 0.241 | 1.442 | 0.170 | 1.435 | 0.163 |
|  | P27 | 1.981 | 0.217 | 1.972 | 0.221 | 1.426 | 0.170 | 1.430 | 0.162 |
|  | P30 | 2.020 | 0.209 | 1.981 | 0.269 | 1.426 | 0.170 | 1.457 | 0.194 |
| Stance Width Hind | P21 | 3.073 | 0.263 | 3.230 | 0.325 | 2.037 | 0.154 | 2.024 | 0.178 |
|  | P24 | 2.881 | 0.231 | 2.929 | 0.222 | 2.005 | 0.154 | 2.002 | 0.155 |
|  | P27 | 2.994 | 0.217 | 2.964 | 0.203 | 2.053 | 0.154 | 2.054 | 0.153 |
|  | P30 | 3.093 | 0.209 | 2.981 | 0.247 | 2.063 | 0.154 | 2.078 | 0.185 |
| Step Angle CV Fore | P21 | 35.756 | 12.442 | 32.232 | 16.187 | 17.879 | 6.945 | 16.293 | 7.992 |
|  | P24 | 34.398 | 10.889 | 33.257 | 11.147 | 18.415 | 6.945 | 18.012 | 6.890 |
|  | P27 | 30.452 | 10.283 | 31.186 | 10.308 | 19.137 | 6.945 | 19.326 | 6.826 |
|  | P30 | 27.408 | 9.817 | 29.872 | 12.096 | 19.285 | 6.945 | 21.085 | 8.301 |
|  |  |  |  |  |  |  |  |  |  |
| Step Angle CV Hind | P21 | 34.271 | 11.444 | 34.406 | 14.936 | 25.094 | 7.546 | 23.511 | 8.428 |
|  | P24 | 34.320 | 10.007 | 34.358 | 10.437 | 25.308 | 7.546 | 24.907 | 7.329 |
|  | P27 | 32.841 | 9.466 | 32.811 | 9.690 | 21.260 | 7.546 | 21.449 | 7.265 |
|  | P30 | 33.102 | 9.004 | 33.021 | 11.088 | 22.675 | 7.546 | 24.471 | 8.738 |
| Step Angle Fore | P21 | 49.302 | 11.285 | 52.597 | 14.717 | 61.505 | 9.316 | 63.163 | 10.672 |
|  | P24 | 53.759 | 9.873 | 54.765 | 10.128 | 62.963 | 9.316 | 63.384 | 9.277 |
|  | P27 | 52.474 | 9.329 | 51.787 | 9.363 | 62.432 | 9.316 | 62.234 | 9.196 |
|  | P30 | 57.773 | 8.894 | 55.500 | 11.002 | 63.263 | 9.316 | 61.382 | 11.065 |
| Step Angle Hind | P21 | 42.038 | 11.684 | 41.826 | 15.312 | 53.026 | 10.122 | 54.715 | 11.500 |
|  | P24 | 47.103 | 10.218 | 47.043 | 10.663 | 52.189 | 10.122 | 52.618 | 10.129 |
|  | P27 | 45.249 | 9.664 | 45.295 | 9.894 | 52.079 | 10.122 | 51.878 | 10.050 |
|  | P30 | 46.800 | 9.194 | 46.943 | 11.379 | 53.779 | 10.122 | 51.863 | 11.889 |
| Stride Frequency Fore | P21 | 4.394 | 0.355 | 4.069 | 0.362 | 4.242 | 0.364 | 4.068 | 0.348 |
|  | P24 | 4.235 | 0.311 | 4.140 | 0.251 | 4.161 | 0.364 | 4.116 | 0.304 |
|  | P27 | 4.058 | 0.293 | 4.128 | 0.233 | 4.289 | 0.364 | 4.310 | 0.301 |
|  | P30 | 4.001 | 0.280 | 4.226 | 0.269 | 4.021 | 0.364 | 4.219 | 0.360 |
| Stride Frequency Hind | P21 | 4.363 | 0.346 | 4.050 | 0.349 | 4.234 | 0.345 | 4.080 | 0.326 |
|  | P24 | 4.233 | 0.303 | 4.144 | 0.245 | 4.155 | 0.345 | 4.116 | 0.286 |
|  | P27 | 4.072 | 0.286 | 4.141 | 0.228 | 4.274 | 0.345 | 4.292 | 0.284 |
|  | P30 | 4.008 | 0.273 | 4.218 | 0.259 | 4.042 | 0.345 | 4.218 | 0.337 |
| Stride Length CV Fore | P21 | 18.025 | 5.681 | 18.566 | 7.396 | 13.991 | 3.606 | 13.711 | 4.195 |
|  | P24 | 18.330 | 4.968 | 18.497 | 5.169 | 14.065 | 3.606 | 13.994 | 3.640 |
|  | P27 | 16.462 | 4.699 | 16.346 | 4.799 | 15.671 | 3.606 | 15.705 | 3.608 |
|  | P30 | 15.639 | 4.470 | 15.273 | 5.490 | 15.701 | 3.606 | 16.018 | 4.351 |
| Stride Length CV Hind | P21 | 12.543 | 4.280 | 14.773 | 5.400 | 12.593 | 3.523 | 12.107 | 4.085 |
|  | P24 | 14.708 | 3.747 | 15.392 | 3.697 | 13.661 | 3.523 | 13.538 | 3.535 |
|  | P27 | 14.110 | 3.538 | 13.647 | 3.408 | 12.817 | 3.523 | 12.875 | 3.504 |
|  | P30 | 13.385 | 3.379 | 11.858 | 4.057 | 13.025 | 3.523 | 13.576 | 4.240 |
| Stride Length Fore | P21 | 4.658 | 0.387 | 5.012 | 0.390 | 4.839 | 0.420 | 5.029 | 0.400 |
|  | P24 | 4.830 | 0.339 | 4.933 | 0.272 | 4.905 | 0.420 | 4.953 | 0.350 |
|  | P27 | 5.050 | 0.320 | 4.973 | 0.252 | 4.782 | 0.420 | 4.759 | 0.347 |
|  | P30 | 5.096 | 0.305 | 4.852 | 0.290 | 5.097 | 0.420 | 4.882 | 0.414 |
| Stride Length Hind | P21 | 4.706 | 0.381 | 5.050 | 0.385 | 4.853 | 0.401 | 5.024 | 0.382 |
|  | P24 | 4.833 | 0.334 | 4.929 | 0.271 | 4.932 | 0.401 | 4.975 | 0.336 |
|  | P27 | 5.026 | 0.315 | 4.949 | 0.252 | 4.808 | 0.401 | 4.788 | 0.333 |
|  | P30 | 5.103 | 0.301 | 4.871 | 0.286 | 5.111 | 0.401 | 4.917 | 0.396 |
| Swing Duration CV Fore | P21 | 24.963 | 8.376 | 24.328 | 10.744 | 18.839 | 4.734 | 18.133 | 5.463 |
|  | P24 | 27.449 | 7.326 | 27.244 | 7.614 | 20.257 | 4.734 | 20.078 | 4.726 |
|  | P27 | 26.440 | 6.927 | 26.593 | 7.080 | 21.294 | 4.734 | 21.378 | 4.684 |
|  | P30 | 23.839 | 6.593 | 24.245 | 7.958 | 21.123 | 4.734 | 21.924 | 5.670 |
|  |  |  |  |  |  |  |  |  |  |
|  |  |  |  |  |  |  |  |  |  |
|  |  |  |  |  |  |  |  |  |  |
| Swing Duration CV Hind | P21 | 19.867 | 6.699 | 21.937 | 8.809 | 21.423 | 5.306 | 21.023 | 6.184 |
|  | P24 | 19.988 | 5.866 | 20.623 | 6.025 | 19.278 | 5.306 | 19.177 | 5.351 |
|  | P27 | 19.781 | 5.536 | 19.365 | 5.550 | 17.737 | 5.306 | 17.785 | 5.302 |
|  | P30 | 20.710 | 5.295 | 19.275 | 6.628 | 17.538 | 5.306 | 17.992 | 6.418 |
| Swing Duration Fore | P21 | 0.098 | 0.015 | 0.109 | 0.018 | 0.098 | 0.008 | 0.101 | 0.009 |
|  | P24 | 0.093 | 0.013 | 0.096 | 0.013 | 0.097 | 0.008 | 0.098 | 0.008 |
|  | P27 | 0.097 | 0.012 | 0.095 | 0.012 | 0.095 | 0.008 | 0.095 | 0.008 |
|  | P30 | 0.100 | 0.012 | 0.093 | 0.014 | 0.101 | 0.008 | 0.097 | 0.009 |
| Swing Duration Hind | P21 | 0.088 | 0.010 | 0.089 | 0.013 | 0.086 | 0.013 | 0.090 | 0.014 |
|  | P24 | 0.087 | 0.009 | 0.087 | 0.009 | 0.082 | 0.013 | 0.084 | 0.012 |
|  | P27 | 0.088 | 0.008 | 0.088 | 0.008 | 0.075 | 0.013 | 0.074 | 0.012 |
|  | P30 | 0.090 | 0.008 | 0.089 | 0.010 | 0.080 | 0.013 | 0.075 | 0.014 |
