# Supplementary material for: The trajectory of gait development in mice
Source: Brain Behav. 2020 Apr 24;10(6):e01636. doi: 10.1002/brb3.1636 (PMC7303394; doi:10.1002/brb3.1636)
Supplement: Supplementary file 15 — Data S1 [file BRB3-10-e01636-s015.pdf]

*Supplementary Material*

# The trajectory of gait development in mice

**Shyam K. Akula, Katherine B. McCullough, Claire Weichselbaum, Joseph D. Dougherty, Susan E. Maloney**

## 1. Supplementary Methods – Detailed Methods Description

### *Gait Analysis*

*Apparatus.* Gait data was collected using the DigiGait Imaging System (Mouse Specifics, Inc), an advanced gait analysis system with Ventral Plane Imaging Technology that generates digital paw prints from the animal as it runs on a motorized treadmill (Hampton et al., 2004). This system has been described in detail previouslyhttps://www.zotero.org/google-docs/?unSJpg (Hampton et al., 2004). Due to smaller body size of juvenile mice, 0.6 cm thick bumpers made from expanded PVC were placed on the sides of the apparatus using magnets to narrow the width of the run alley and thus increase the potential for straight, usable runs. The floor of the apparatus was made of tempered glass around which looped a transparent belt (PCV and HDPE blend) measuring 6 cm wide. The animal was sequestered to the 40.5 cm run alley by a transparent polycarbonate enclosure and illuminated to 1381 lux from above and below with LED panel lighting. A digital video camera (Basler Ace/Scout Camera with 12.5/16mm lens) was housed beneath the run alley to capture the ventral plane image of the animal, which was sent to the DigiGait software on a connected computer.

*Procedure.* Both male and female experimenters collected C57 gait data and the female experimenter collected FVB gait data. Pilot testing revealed FVB mice can adequately run on the DigiGait by P17. However, C57 mice could not run adequately until P21. Therefore, we chose to examine gait across the post-weaning, juvenile period (Figure 1A). All testing occurred during the light phase of the circadian cycle. For all habituation and testing sessions, the mice were placed in a holding room adjacent to the testing environment for a 30 min acclimation period prior to testing, and weighed. Prior to testing the paws of the FVB mice were dyed with red food coloring (McCormick) diluted into water to increase contrast with belly fur. The testing room overhead lights were off during testing for optimal video capture, however the holding room lights remained on to decrease disruptions of circadian rhythms. On P20, each mouse was habituated to the apparatus. This consisted of placing the animal on the stationary belt and starting the belt moving at 5 cm/s and slowly increasing the speed until 20 cm/s is reached allowing for at least 30 sec of run time. Because speed is the greatest influencer of gait, the speed of the treadmill during data collection was kept constant across all ages at 20 cm/s to allow for appropriate comparisons of forced gait across age. Testing occurred P21, P24, P27 and P30. For these test days, each mouse was placed individually on the apparatus. The belt was started at 10 cm/s until the animal started walking forward. Once the animal reached the front of the alley, the speed was increased to 20 cm/s. Because speed is the greatest influencer of gait, the speed of the treadmill during data collection was kept constant across all ages at 20 cm/s to allow for appropriate comparisons of forced gait across age. Once a usable run was acquired, the belt was stopped and the animal removed to the homecage. Criteria for a usable run included a consistent forward movement with no sliding, jumping, or side drift. The belt was cleaned with 70% EtOH between litters or as needed between mice and daily upon completion of testing.

*Video Processing.* To obtain optimal contrast between the mouse paws and coat color for recognition of paws for digital footprint generation and processing of videos through the DigiGait software pipeline, we post-processed the videos using ImageJ (Wayne Rasband, NIH, USA, RRID:SCR_003070). To determine optimal levels of brightness and contrast we used two videos from each time point and processed each multiple times using differing levels of brightness and contrast. The settings that resulted in the smoothest paw area contact plots were used (brightness adjustment of 19 and contrast adjustment of 135). These adjustments were then applied to all videos. The adjusted videos were exported as uncompressed .avi files to ensure compatibility with the DigiGait software.

Each video was then processed through the DigiGait Analysis software, as described previously (Hampton et al., 2004). This entailed applying filters to exclude the snout and adjust contrast for optimal digital footprint generation. Paw contact area plots were generated and validated against the video of the mouse. Any tracking errors were manually corrected. The digital footprints and paw contact area plots were then used to extract the

gait parameters used for analysis (Figure 1B). Gait was analyzed by quantifying components of the step cycle, or stride, broken into when a paw has contact with the ground, known as the stance phase, and when it is moving through the air, known as the swing phase (Figure 1B). The stance phase is further broken down into the paw braking phase (heel strike to full stance) and propulsion phase (full stance to toe push off). All our trials comprised at least 12 strides based on previous work suggesting 9 strides or more are required for DigiGait data processing (Hampton et al., 2004) (C57, *M*=19.0, range: 12.0-24.5; FVB, *M*=18.9, range: 13.0-25.5).

*Selection of gait metrics for analysis.* The DigiGait software analysis system outputs a comprehensive list of gait metrics. A concern with such a large list of variables is inflation of familywise error rate. To identify possible redundant variables, we generated scatterplot matrices. Pairs of variables that were visibly perfectly aligned were considered redundant and one variable within the pair was excluded from further analysis. The variables we excluded were stride duration, % brake of stride, % brake of stance, % propulsion of stride, % propulsion of stance, swing/stance ratio (Figures S1, S2), hindlimb shared stance time (Figures S3, S4), and ataxia coefficient (Figures S5, S6)

In addition to excluding variables based on redundancy, we also determined if variables required exclusion based on poor reliability. The post-video acquisition processing within the DigiGait software requires adjustment of filters to remove the snout and decrease noise from the digital paw prints, as well as manual corrections to errors within the paw contact area plots, introducing the possibility of inconsistencies across experimenters. We examined the inter-rater reliability of this processing between the measurements produced by two independent experimenters by calculating intraclass correlation coefficients (ICC) with their 95% confidence intervals using IBM SPSS Statistic software (v.25) based on absolute-agreement and two-way mixed-effects model. The data used for this were derived from an independent cohort of 10 FVB mice tested on P24, P27 and P30. For fore and hind limbs, respectively, 20/25 and 27/32 metrics showed excellent reliability (ICC ≥ .75) and another 2/25 and 2/32 showed good reliability (ICC = .60 - .74). The remaining metrics showed poor reliability (ICC ≤ .39; 3/25 and 3/32). ICCs are reported in Table 1. We excluded from further analysis any metrics with poor reliability: midline distance, axis distance, paw drag, and maximal rate of change of paw area contact during the propulsion phase. We also excluded tau propulsion despite good reliability because we felt this measure has not yet been defined or validated adequately in the literature and thus its usefulness is not clear.

***Body Length Quantification***

Animal body length was measured by importing videos from the Digigait software into Ethovision (Noldus Information Technology, The Netherlands, RRID:SCR_000441). The animal's body was automatically detected using the contrast of the darker body against a lighter background. A custom script provided by Noldus was used to calculate its length based on the coordinates of the nose, center point and tail base. A length measurement was calculated from those three coordinates for every frame of the video and then averaged into one score per animal for analysis. Thus, this accounted for any differences that occurred due to extensions or contractions of the body during a stride. To validate this method of body length measurement, the body lengths of a subset of FVB and C57 mice were also measured manually from the same videos at three different time points and then averaged. Intraclass correlation coefficients (ICC) with their 95% confidence intervals were calculated between the manual and automated measurements using IBM SPSS Statistics software (v.25, RRID:SCR_002865) based on absolute-agreement and two-way mixed-effects model. The ICCs indicated excellent reliability between the manual and automated measurements (FVB, ICC = .972, 95% CI [.904, .992]; C57, ICC = .977, 95% CI [.955, .988]), providing confidence in and validation of our automated process for body length measurement.

## 2. Supplementary Figures

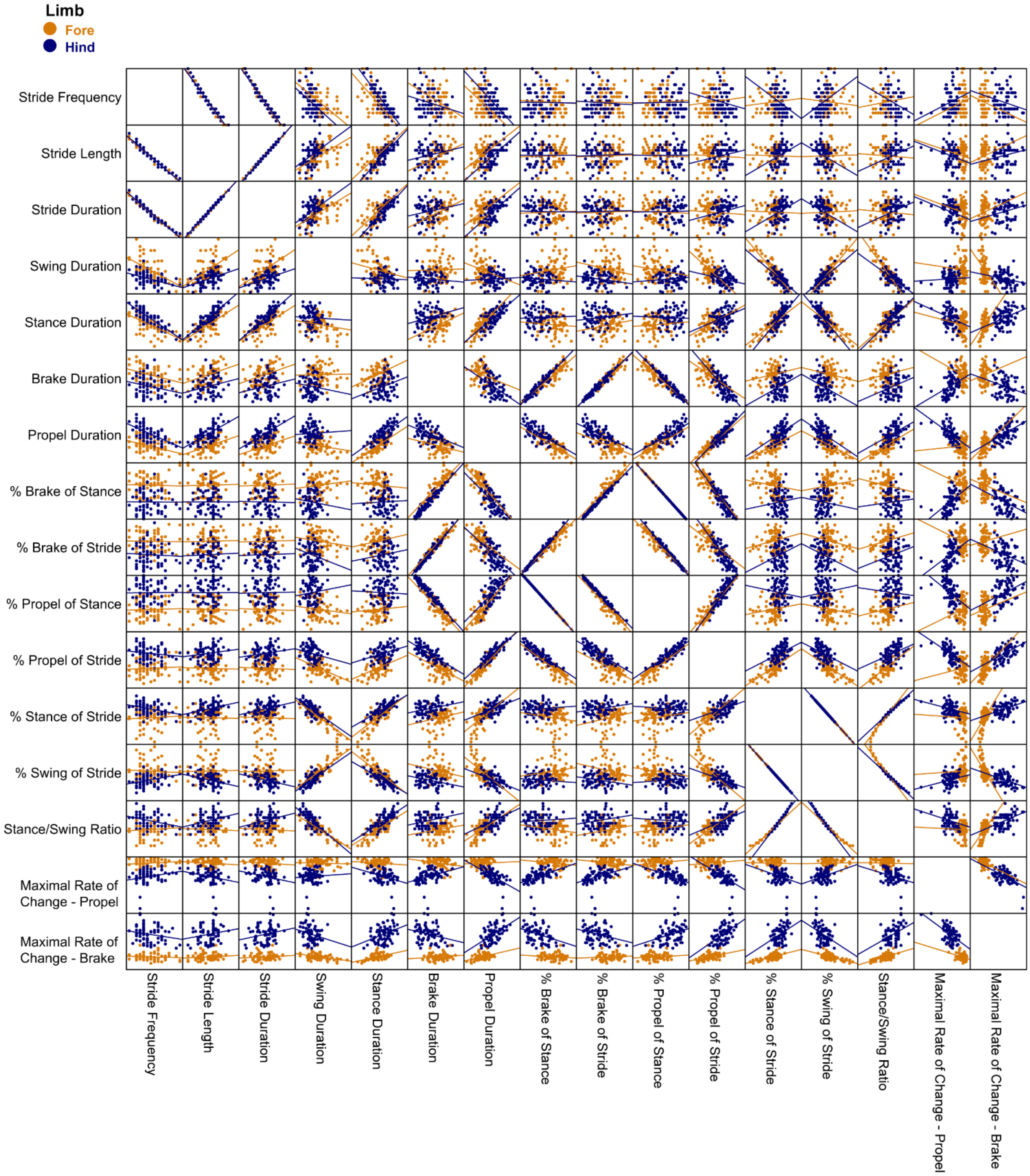


**Supplementary Figure 1. Scatter matrices of spatiotemporal metrics measured in both fore and hindlimbs of C57 mice at P21, P24, P27 and P30.** One variable from a pair that was considered close to or at perfect alignment was excluded from further analysis.

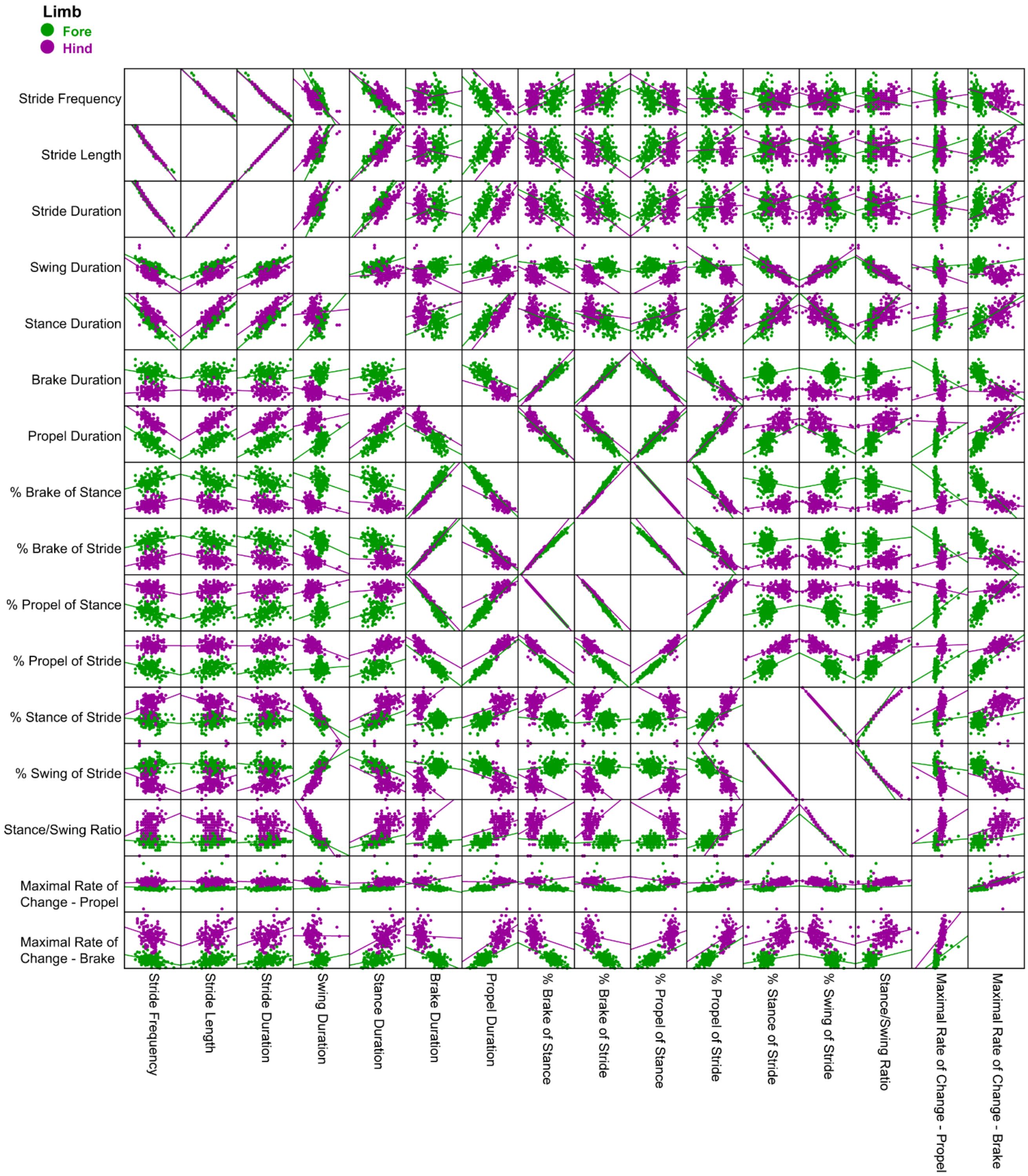


**Supplementary Figure 2. Scatter matrices of spatiotemporal metrics measured in both fore and hindlimbs of FVB mice at P21, P24, P27 and P30.** One variable from a pair that was considered close to or at perfect alignment was excluded from further analysis.

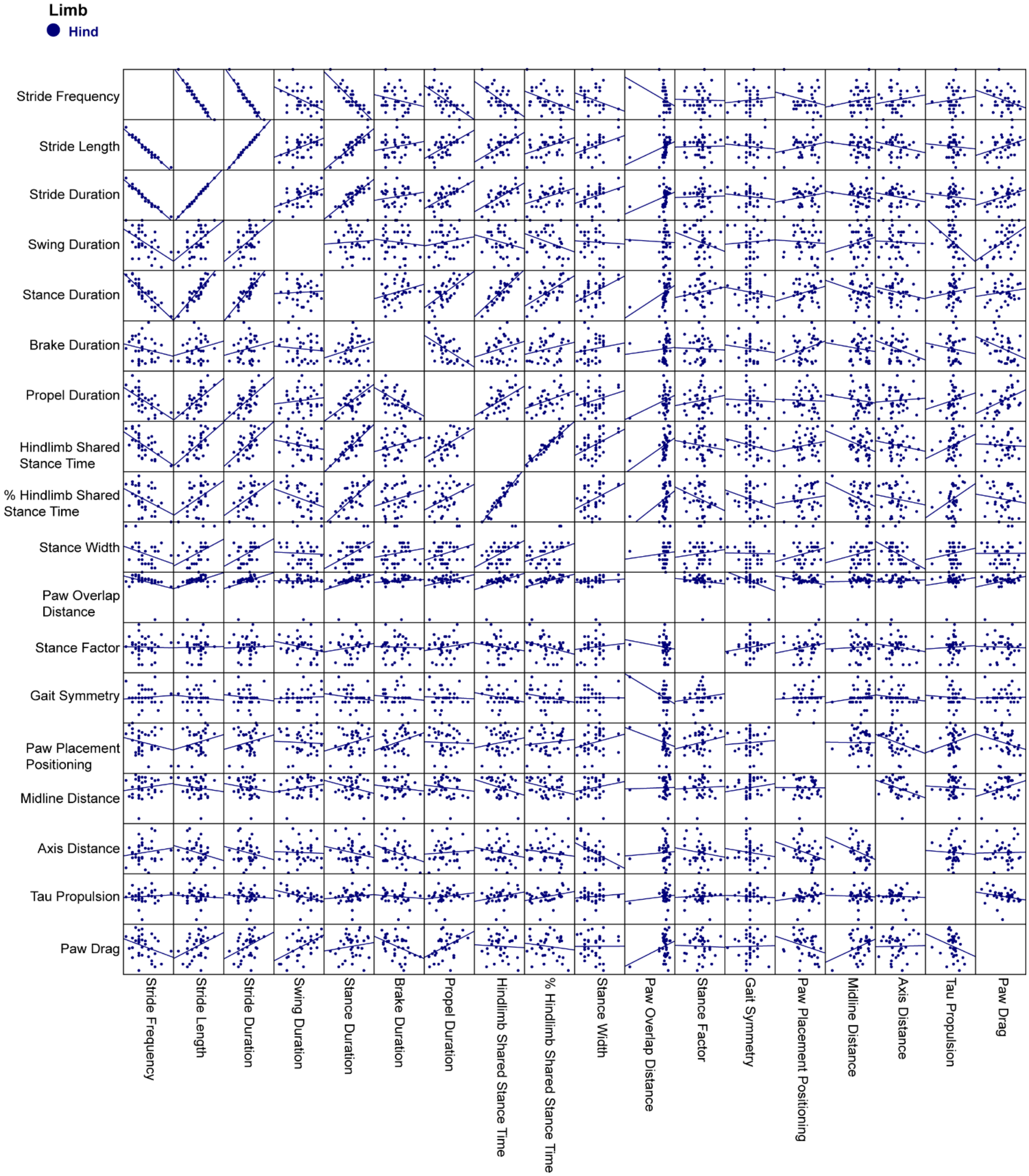


**Supplementary Figure 3. Scatter matrices of spatiotemporal metrics measured in only the hindlimbs of C57 mice at P21, P24, P27 and P30.** One variable from a pair that was considered close to or at perfect alignment was excluded from further analysis.

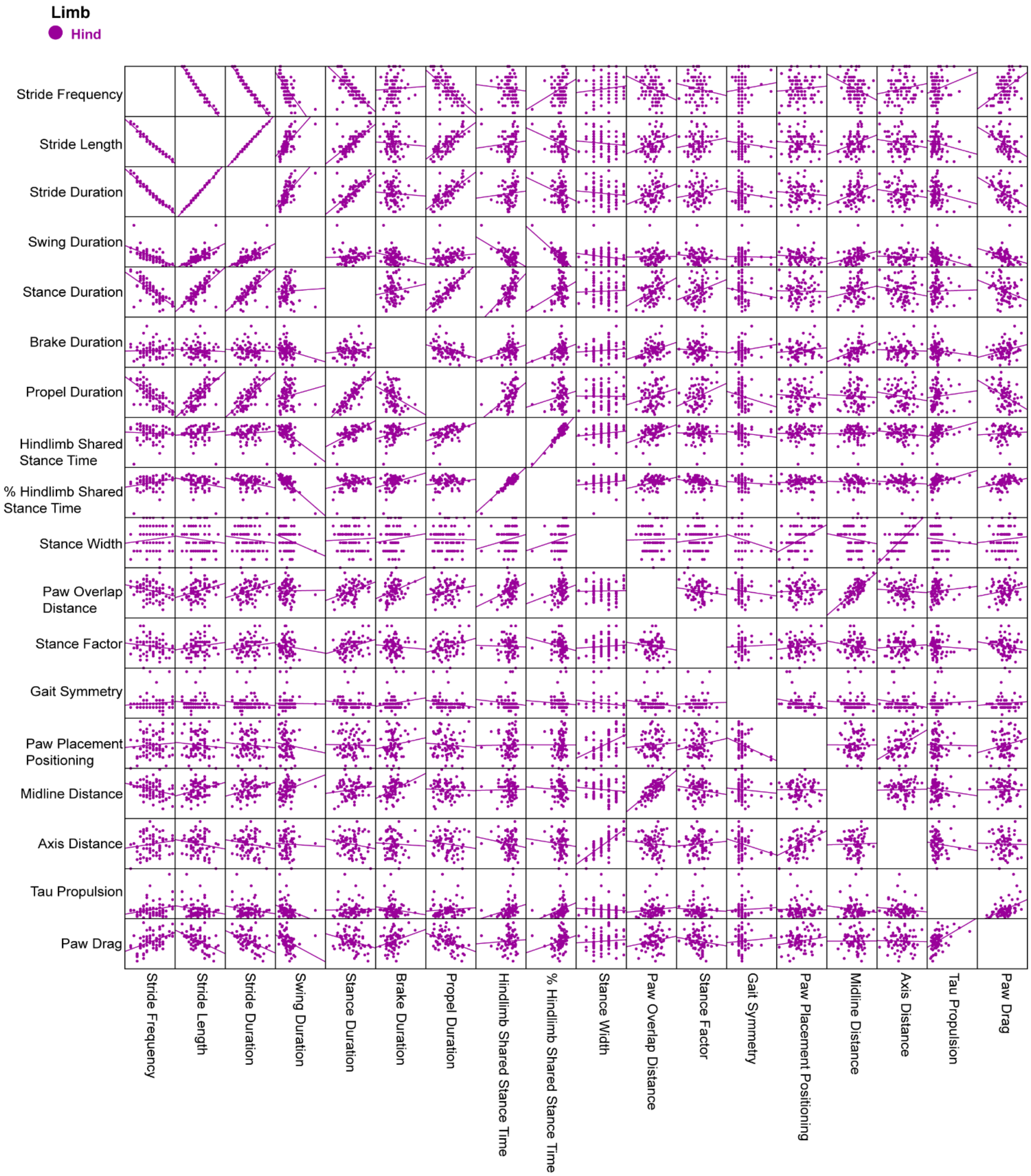


**Supplementary Figure 4. Scatter matrices of spatiotemporal metrics measured in only the hindlimbs of FVB mice at P21, P24, P27 and P30.** One variable from a pair that was considered close to or at perfect alignment was excluded from further analysis.

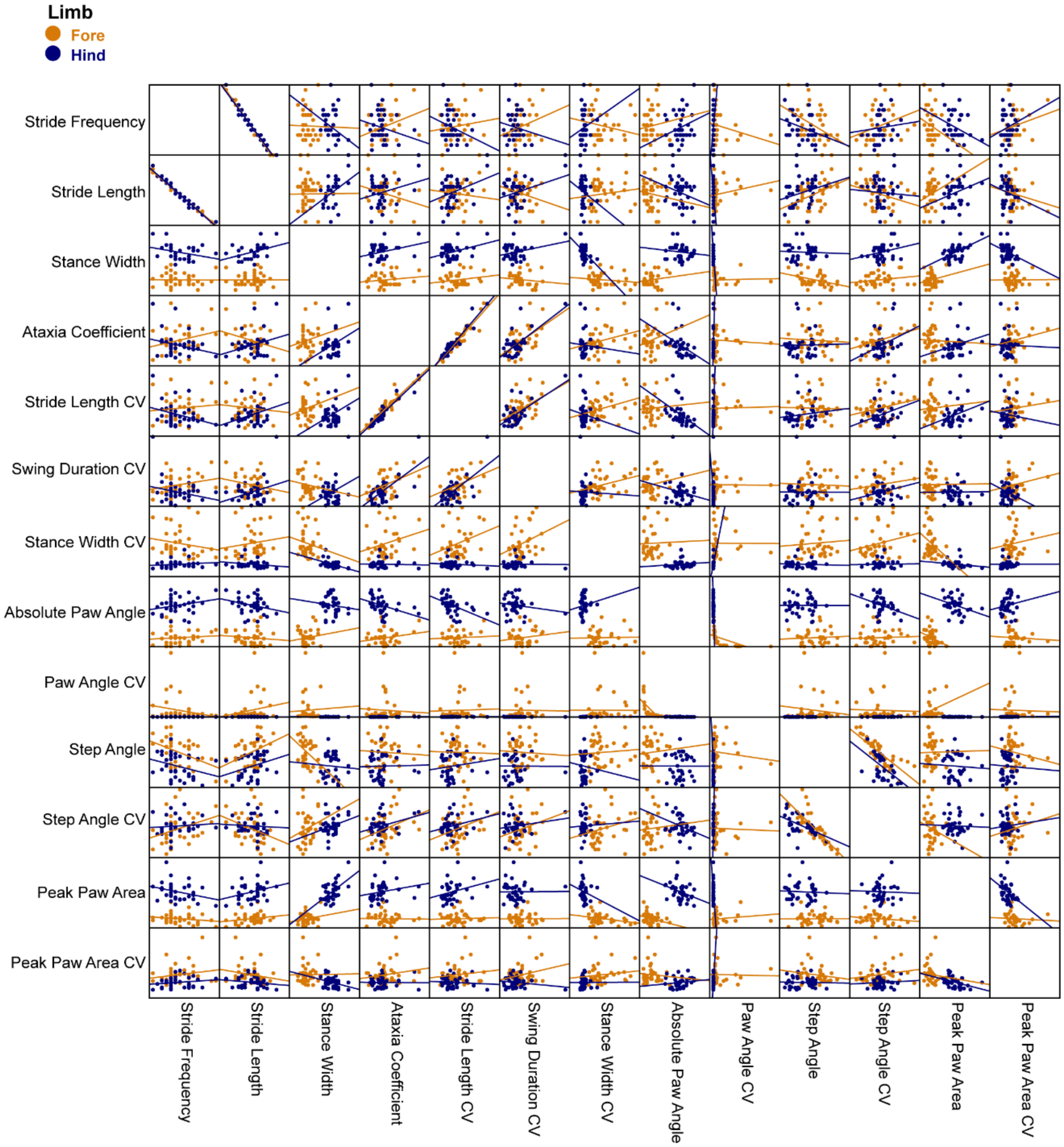


**Supplementary Figure 5. Scatter matrices of postural and variability metrics measured in both fore and hindlimbs of C57 mice at P21, P24, P27 and P30.** One variable from a pair that was considered close to or at perfect alignment was excluded from further analysis.

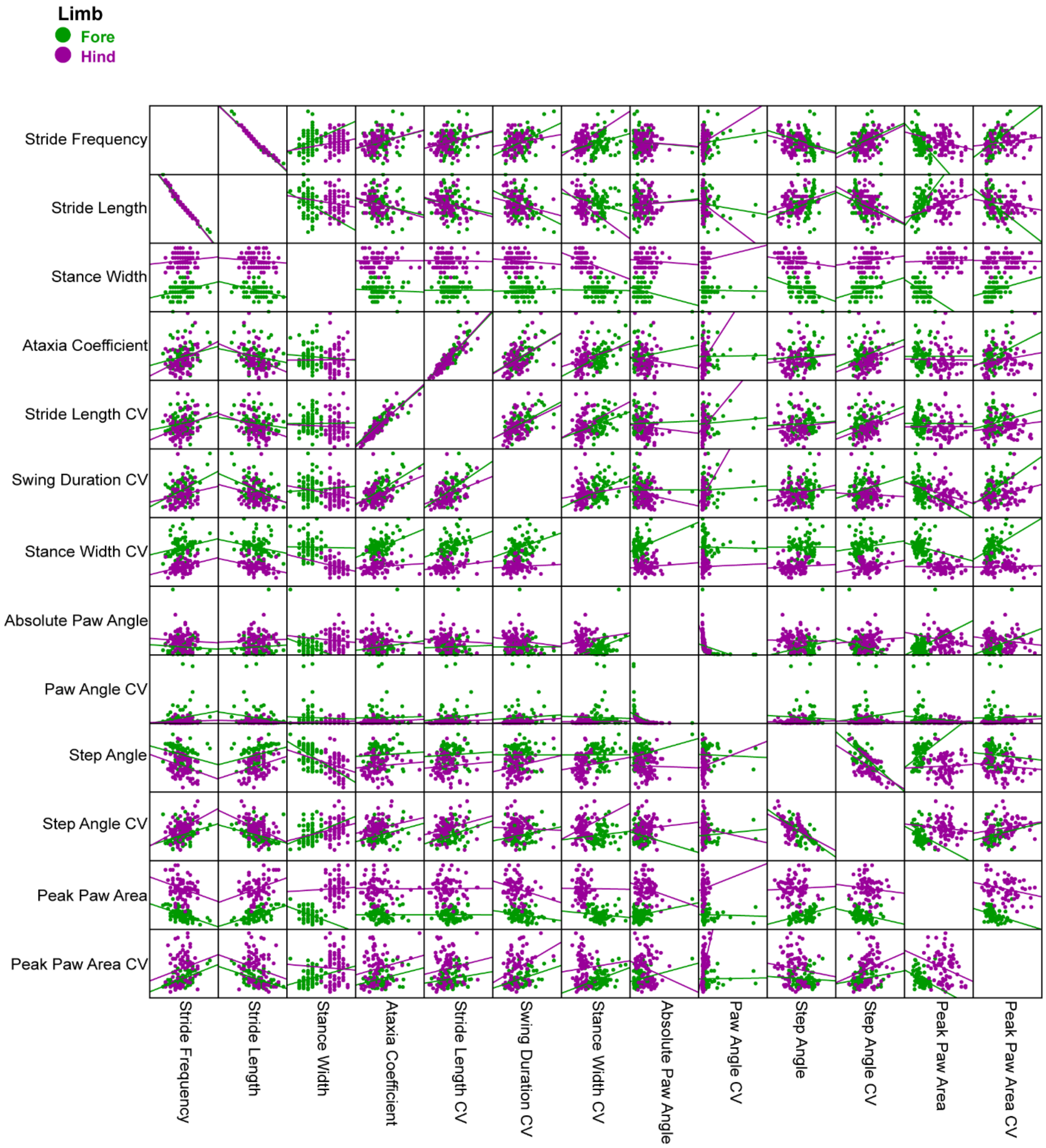


**Supplementary Figure 6. Scatter matrices of Scatter matrices of postural and variability metrics measured in both fore and hindlimbs of FVB mice at P21, P24, P27 and P30.** One variable from a pair that was considered close to or at perfect alignment was excluded from further analysis.

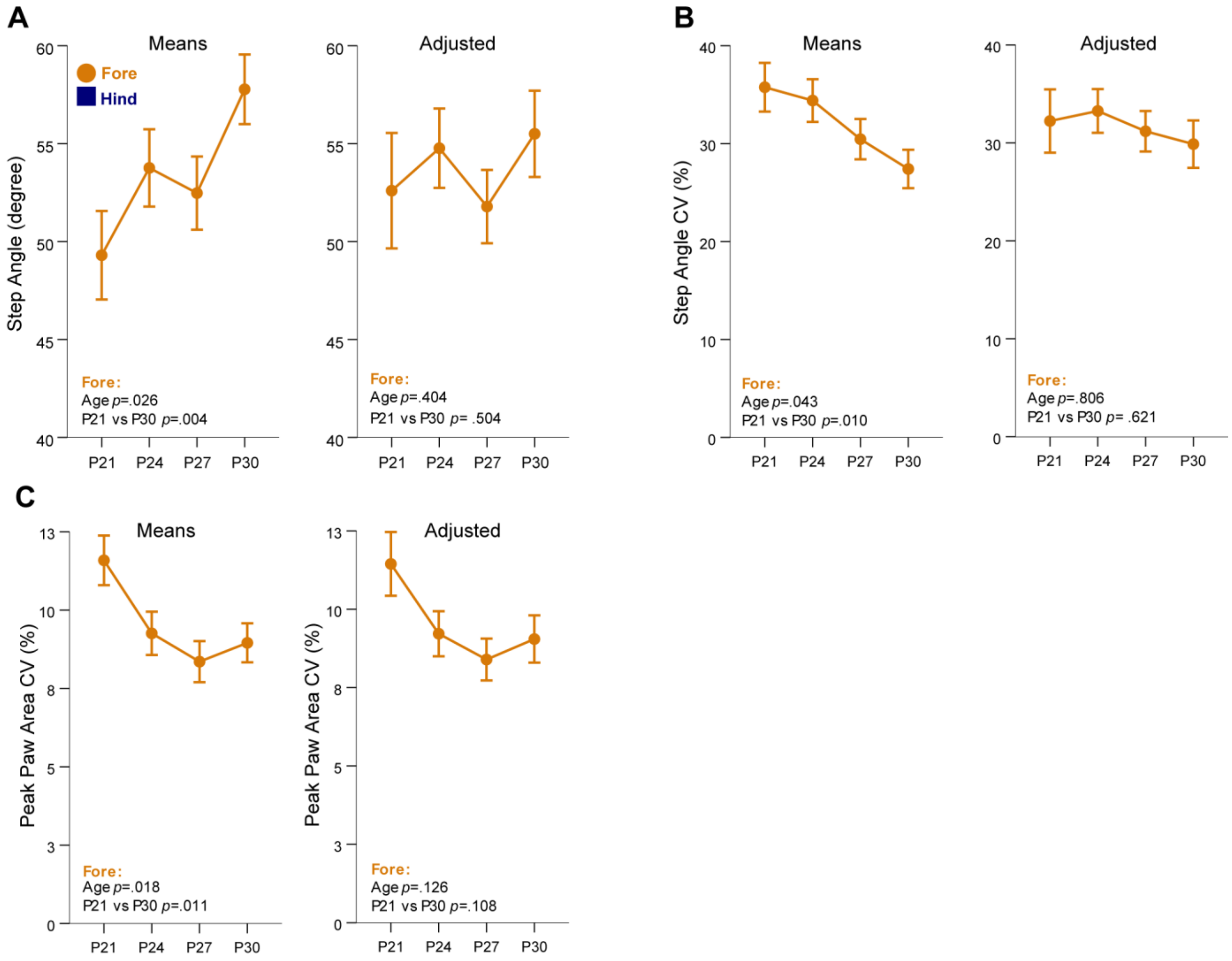


**Supplementary Figure 7. Gait metrics that only reflected change in body length in C57 mice.** Raw data means and covariate adjusted means are presented for all three variables. (**A**) Step Angle, (**B**) step angle coefficient of variance (CV), and (**C**) peak paw area CV appeared to significantly change with age in C57 mice. However, after adjusting for differences in body length from P21 to P30, these variables no longer significantly changed with age. Data are means ± SEM and covariate adjusted means ± SEM.

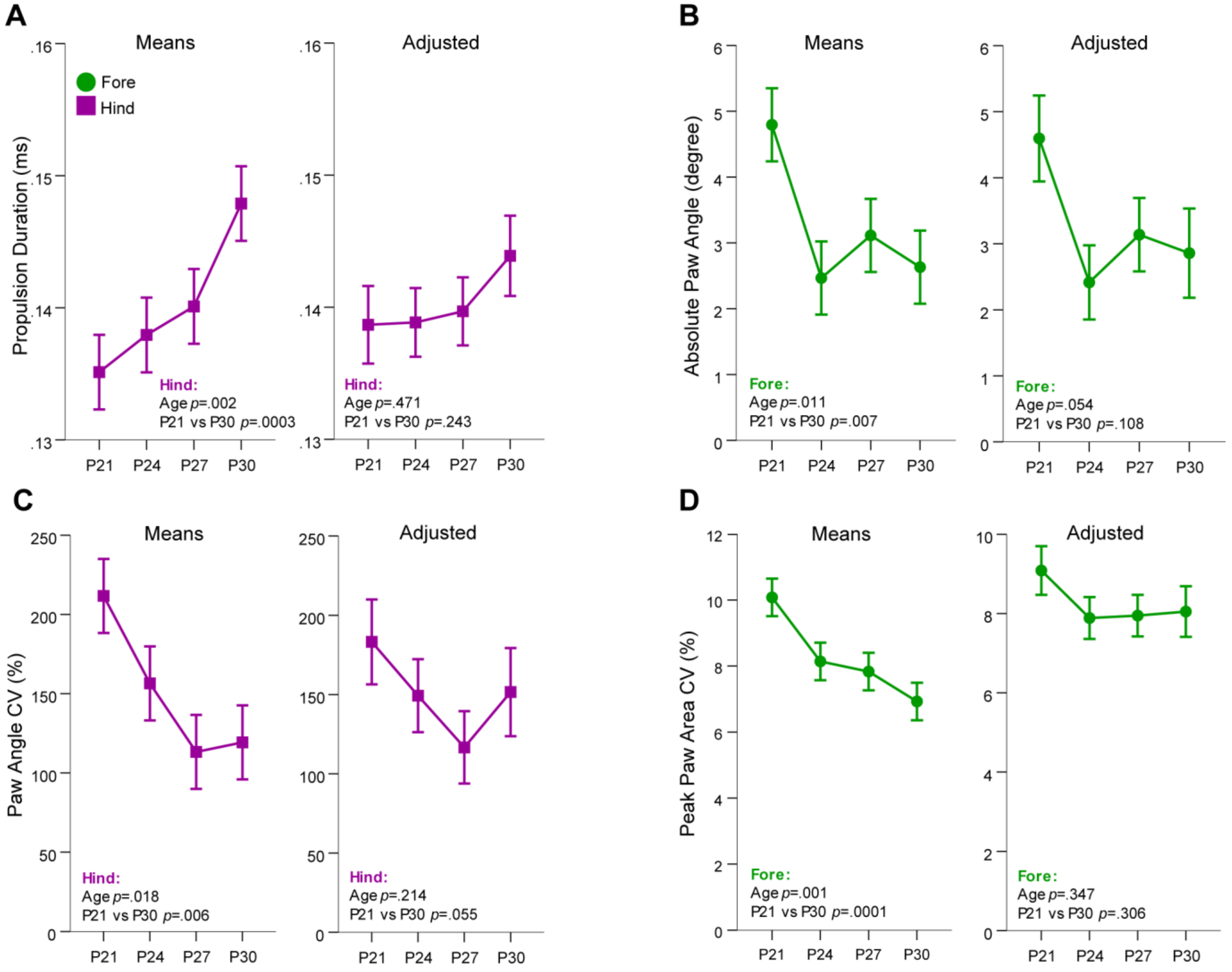


**Supplementary Figure 8. Gait metrics that only reflected change in body length in FVB mice.** Raw data means and covariate adjusted means are presented for all variables. (**A**) Propulsion duration, (**B**) absolute paw angle, (**C**) paw angle coefficient of variance (CV), and (**D**) peak paw area CV appeared to significantly change with age in FVB mice. However, after adjusting for differences in body length from P21 to P30, these variables no longer significantly changed with age. Data are means ± SEM and covariate adjusted means ± SEM.

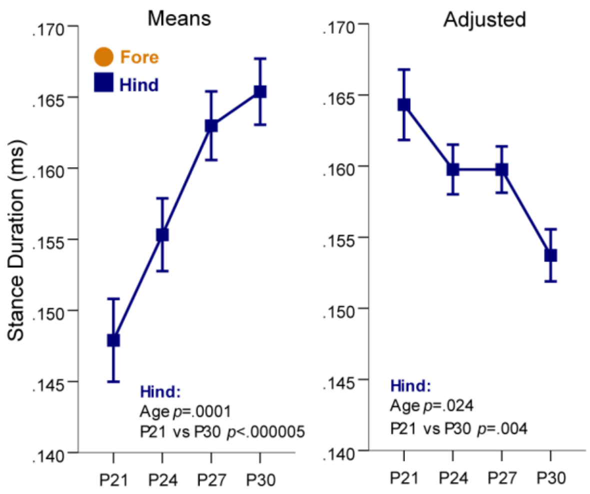


**Supplementary Figure 9. The directional trajectory of hindlimb stance duration in C57 from P21 to P30 is flipped when adjusted for body length changes.** Hindlimb stance duration raw means and covariate adjusted means are presented for C57 mice. Before adjusting for body length, stance duration appeared to significantly increase. However, after adjusting for differences in body length from P21 to P30, stance duration significantly decreased from P21 to P30. Data are means ± SEM and covariate adjusted means ± SEM.

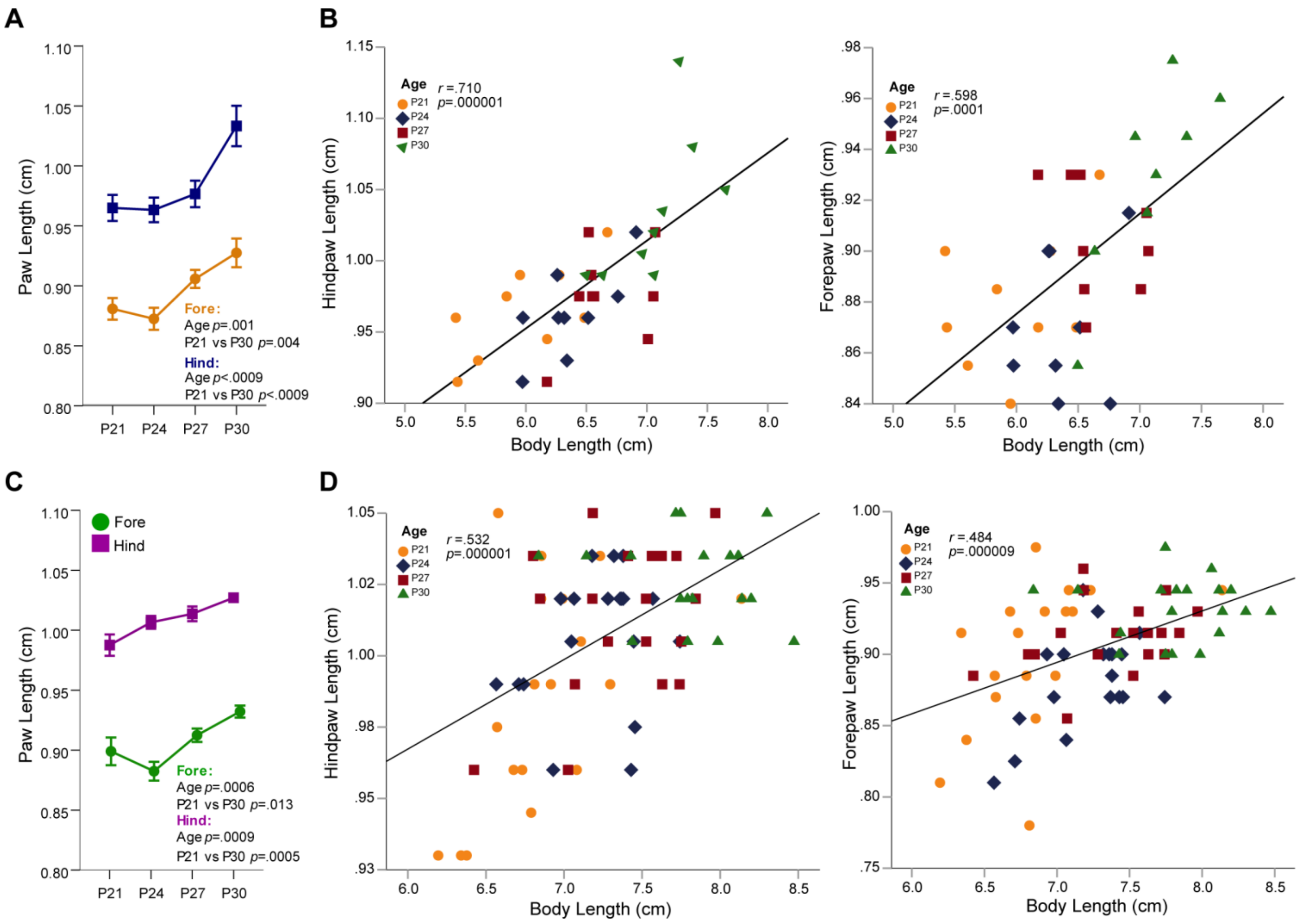


**Supplementary Figure 10. Scatter matrices of Scatter matrices of postural and variability metrics measured in both fore and hindlimbs of FVB mice at P21, P24, P27 and P30. (A)** C57 fore- and hindpaw lengths increase with age, as measured at full stance. Data are means ± SEM. (**B**) Scatterplots of the strong positive relationship between body length and fore- and hindpaw lengths from P21 to P30 in C57 mice. All data represented. **(C)** FVB fore- and hind paw lengths increase with age, as measured at full stance. Data are means ± SEM. (**D**) Scatterplots of the strong positive relationship between body length and fore- and hindpaw lengths from P21 to P30 in FVB mice. All data represented.

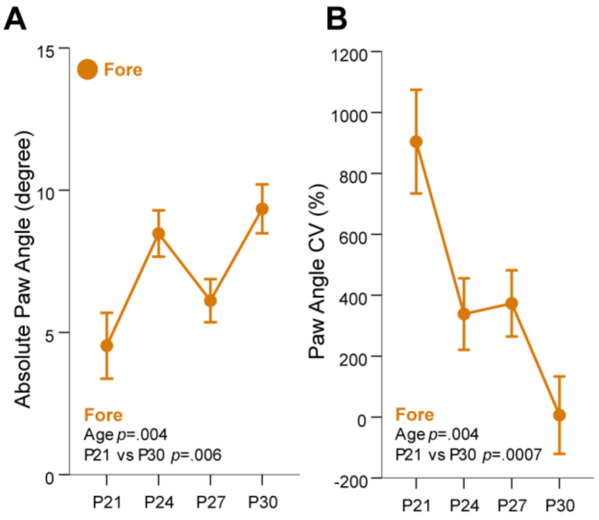


**Supplementary Figure 11. Absolute paw angle and paw able coefficient of variation for C57 forelimbs.** (**A**) The postural metric absolute paw angle significantly changed across P21 to P30 for the forelimbs in C57 mice, but meaning of the change across time is unclear. (**B**) The variability of this metric, paw angle CV, significantly decreased from P21 to P30.

# 3. Supplementary Tables

## Supplementary Table 1. Statistical output for C57 gait data.

| OUTCOME | NORMAL | LMM | parameters | *t* | sig. | LMM (adjusted for body length) | parameters | *t* | sig. |
|---|---|---|---|---|---|---|---|---|---|
| %Hindlimb Shared Stance | normal | Age F(3,44.19)=1.92, p=0.140 | P21 vs. P30 | -2.140 | 0.036 | Age F(3,38.60)=1.27, p=0.300 | P21 vs. P30 | 1.118 | 0.268 |
| | | | P24 vs. P30 | -0.406 | 0.686 | Body Length F(1,49.65)=12.30, p=0.0010 | P24 vs. P30 | 1.904 | 0.061 |
| | | | P27 vs. P30 | -0.405 | 0.687 | | P27 vs. P30 | 1.252 | 0.216 |
| % Stance Fore | P30 nonnormal | Age F(3,44.79)=8.15, p=0.0002 | P21 vs. P30 | -3.099 | 0.003 | Age F(3,43.93)=8.48, p=0.0001 | P21 vs. P30 | -2.989 | 0.004 |
| | | | P24 vs. P30 | 0.906 | 0.368 | Body Length F(1,68.81)=1.89, p=0.174 | P24 vs. P30 | -0.254 | 0.800 |
| | | | P27 vs. P30 | 0.853 | 0.398 | | P27 vs. P30 | 0.035 | 0.972 |
| % Stance Hind | normal | Age F(3,44.28)=2.43, p=0.077 | P21 vs. P30 | -2.438 | 0.017 | Age F(3,37.90)=1.07, p=0.373 | P21 vs. P30 | 1.068 | 0.290 |
| | | | P24 vs. P30 | -1.048 | 0.299 | Body Length F(1,51.43)=12.52, p=0.0009 | P24 vs. P30 | 1.470 | 0.146 |
| | | | P27 vs. P30 | 0.001 | 0.999 | | P27 vs. P30 | 1.707 | 0.093 |
| % Swing Fore | P30 nonnormal | Age F(3,44.79)=8.15, p=0.0002 | P21 vs. P30 | 3.099 | 0.003 | Age F(3,43.93)=8.48, p=0.0001 | P21 vs. P30 | 2.989 | 0.004 |
| | | | P24 vs. P30 | -0.906 | 0.368 | Body Length F(1,68.81)=1.89, p=0.174 | P24 vs. P30 | 0.254 | 0.800 |
| | | | P27 vs. P30 | -0.853 | 0.398 | | P27 vs. P30 | -0.035 | 0.972 |
| % Swing Hind | normal | Age F(3,44.28)=2.43, p=0.077 | P21 vs. P30 | 2.438 | 0.017 | Age F(3,37.90)=1.072, p=0.37 | P21 vs. P30 | -1.068 | 0.290 |
| | | | P24 vs. P30 | 1.048 | 0.299 | Body Length F(1,51.43)=12.52, p=0.0009 | P24 vs. P30 | -1.470 | 0.146 |
| | | | P27 vs. P30 | -0.001 | 0.999 | | P27 vs. P30 | -1.707 | 0.093 |
| Absolute Paw Angle Fore | normal | Age F(3,51.45)=5.02, p=0.004 | P21 vs. P30 | -2.404 | 0.019 | Age F(3,43.11)=5.21, p=0.004 | P21 vs. P30 | -2.876 | 0.006 |
| | | | P24 vs. P30 | 0.467 | 0.642 | Body Length F(1,42.18)=2.83, p=0.10 | P24 vs. P30 | -0.680 | 0.499 |
| | | | P27 vs. P30 | -2.551 | 0.014 | | P27 vs. P30 | -2.950 | 0.005 |
| Absolute Paw Angle Hind | normal | Age F(3,50.47)=2.45, p=0.07 | P21 vs. P30 | 1.957 | 0.054 | Age F(3,46)=1.12, p=0.35 | P21 vs. P30 | -0.365 | 0.716 |
| | | | P24 vs. P30 | 0.540 | 0.591 | Body Length F(1,49.4)=5.59, p=0.022 | P24 vs. P30 | -0.974 | 0.333 |
| | | | P27 vs. P30 | -0.899 | 0.373 | | P27 vs. P30 | -1.689 | 0.097 |
| Brake Duration Fore | normal | Age F(3,48.73)=2.96, p=0.04 | P21 vs. P30 | -0.003 | 0.998 | Age F(3,47.34)=4.77, p=0.005 | P21 vs. P30 | 2.293 | 0.025 |
| | | | P24 vs. P30 | -1.624 | 0.109 | Body Length F(1,65.08)=8.55, p=0.005 | P24 vs. P30 | 0.606 | 0.546 |
| | | | P27 vs. P30 | 0.963 | 0.340 | | P27 vs. P30 | 2.277 | 0.026 |
| Brake Duration Hind | normal | Age F(3,50.84)=15.62, p=2.5E-07 | P21 vs. P30 | -6.631 | 0.000 | Age F(3,42.82)=7.13, p=0.0005 | P21 vs. P30 | -3.817 | 0.0003 |
| | | | P24 vs. P30 | -1.527 | 0.131 | Body Length F(1,49.21)=0.56, p=0.460 | P24 vs. P30 | -0.795 | 0.429 |
| | | | P27 vs. P30 | -1.989 | 0.053 | | P27 vs. P30 | -1.622 | 0.110 |
| Gait Symmetry | normal | Age F(3,60.82)=0.78, p=0.510 | P21 vs. P30 | 1.093 | 0.278 | Age F(3,50.57)=0.40, p=0.76 | P21 vs. P30 | 0.244 | 0.808 |
| | | | P24 vs. P30 | -0.167 | 0.868 | Body Length F(1,45.56)=0.52, p=0.476 | P24 vs. P30 | -0.541 | 0.590 |
| | | | P27 vs. P30 | -0.421 | 0.676 | | P27 vs. P30 | -0.606 | 0.547 |
| Max. Rate Contact Change Fore | normal | Age F(3,49.46)=2.45, p=0.075 | P21 vs. P30 | -1.417 | 0.161 | Age F(3,46.24)=2.03, p=0.12 | P21 vs. P30 | 1.068 | 0.290 |
| | | | P24 vs. P30 | -0.310 | 0.757 | Body Length F(1,56.51)=7.02, p=0.010 | P24 vs. P30 | 1.355 | 0.180 |
| | | | P27 vs. P30 | 1.491 | 0.142 | | P27 vs. P30 | 2.451 | 0.017 |
| Max. Rate Contact Change Hind | normal | Age F(3,49.07)=1.82, p=0.156 | P21 vs. P30 | 0.826 | 0.412 | Age F(3,49.14)=4.94, p=0.004 | P21 vs. P30 | 3.549 | 0.001 |
| | | | P24 vs. P30 | -0.498 | 0.620 | Body Length F(1,43.91)=16.22, p=0.0002 | P24 vs. P30 | 1.716 | 0.091 |
| | | | P27 vs. P30 | 1.623 | 0.111 | | P27 vs. P30 | 2.843 | 0.006 |
| Paw Overlap Distance | P30 nonnormal | Age F(3,41.29)=1.69, p=0.183 | P21 vs. P30 | -0.233 | 0.816 | Age F(3,36.41)=4.92, p=0.006 | P21 vs. P30 | 2.850 | 0.006 |
| | | | P24 vs. P30 | 1.546 | 0.127 | Body Length F(1,42.38)=16.53, p=0.0002 | P24 vs. P30 | 3.771 | 0.0004 |
| | | | P27 vs. P30 | 1.509 | 0.139 | | P27 vs. P30 | 2.716 | 0.009 |
| Paw Angle CV Fore | P21, P24, P27, P30 nonnormal | Age F(3,45.88)=3.87, p=0.015 | P21 vs. P30 | 2.952 | 0.004 | Age F(3,41.67)=5.14, p=0.004 | P21 vs. P30 | 3.595 | 0.001 |
| | | | P24 vs. P30 | 0.519 | 0.606 | Body Length F(1,49.66)=4.66, p=0.036 | P24 vs. P30 | 1.778 | 0.080 |
| | | | P27 vs. P30 | 1.784 | 0.081 | | P27 vs. P30 | 2.476 | 0.016 |
| Paw Angle CV Hind | normal | Age F(3,60.39)=1.93, p=0.134 | P21 vs. P30 | 1.775 | 0.080 | Age F(3,53.27)=1.25, p=0.302 | P21 vs. P30 | 1.487 | 0.142 |
| | | | P24 vs. P30 | 1.931 | 0.058 | Body Length F(1,45.36)=0.11, p=0.739 | P24 vs. P30 | 1.806 | 0.076 |
| | | | P27 vs. P30 | 0.604 | 0.549 | | P27 vs. P30 | 0.671 | 0.505 |
| Paw Placement Positioning | normal | Age F(3,47.39)=0.74, p=0.54 | P21 vs. P30 | -1.418 | 0.160 | Age F(3,46.53)=0.363, p=0.780 | P21 vs. P30 | 0.865 | 0.390 |
| | | | P24 vs. P30 | -0.797 | 0.429 | Body Length F(1,63.03)=5.20, p=0.026 | P24 vs. P30 | 0.857 | 0.394 |
| | | | P27 vs. P30 | -0.924 | 0.360 | | P27 vs. P30 | 0.237 | 0.813 |
| Peak Paw Area CV Fore | normal | Age F(3,58.55)=3.63, p=0.018 | P21 vs. P30 | 2.617 | 0.011 | Age F(3,43.12)=2.01, p=0.126 | P21 vs. P30 | 1.635 | 0.108 |
| | | | P24 vs. P30 | 0.327 | 0.745 | Body Length F(1,40.57)=0.05, p=0.832 | P24 vs. P30 | 0.152 | 0.880 |
| | | | P27 vs. P30 | -0.646 | 0.522 | | P27 vs. P30 | -0.670 | 0.506 |
| Peak Paw Area CV Hind | normal | Age F(3,48.55)=9.13, p=6.7E-05 | P21 vs. P30 | 4.959 | 0.000005 | Age F(3,40.34)=4.26, p=0.011 | P21 vs. P30 | 3.362 | 0.001 |
| | | | P24 vs. P30 | 2.454 | 0.017 | Body Length F(1,46.70)=0.02, p=0.901 | P24 vs. P30 | 2.052 | 0.044 |
| | | | P27 vs. P30 | 0.698 | 0.489 | | P27 vs. P30 | 0.699 | 0.487 |
| Peak Paw Area Fore | normal | Age F(3,47.67)=1.27, p=0.30 | P21 vs. P30 | -0.710 | 0.480 | Age F(3,43.62)=2.26, p=0.095 | P21 vs. P30 | 2.045 | 0.045 |
| | | | P24 vs. P30 | -0.452 | 0.653 | Body Length F(1,50.62)=11.38, p=0.001 | P24 vs. P30 | 1.573 | 0.121 |
| | | | P27 vs. P30 | 1.186 | 0.241 | | P27 vs. P30 | 2.398 | 0.020 |
| Peak Paw Area Hind | normal | Age F(3,45.74)=1.51, p=0.22 | P21 vs. P30 | -0.066 | 0.947 | Age F(3,45.79)=4.68, p=0.006 | P21 vs. P30 | 3.457 | 0.001 |
| | | | P24 vs. P30 | -0.769 | 0.445 | Body Length F(1,41.81)=23.05, p=2.04E-05 | P24 vs. P30 | 1.901 | 0.062 |
| | | | P27 vs. P30 | 1.284 | 0.206 | | P27 vs. P30 | 2.868 | 0.006 |
| Propulsion Duration Fore | normal | Age F(3,49.42)=17.50, p=7.4E-08 | P21 vs. P30 | -6.312 | 2.2E-08 | Age F(3,47.26)=7.94, p=0.0002 | P21 vs. P30 | -2.757 | 0.008 |
| | | | P24 vs. P30 | -0.910 | 0.366 | Body Length F(1,63.77)=2.56, p=0.114 | P24 vs. P30 | 0.328 | 0.744 |
| | | | P27 vs. P30 | -1.005 | 0.319 | | P27 vs. P30 | -0.174 | 0.863 |
| Propulsion Duration Hind | normal | Age F(3,47.93)=2.26, p=0.09 | P21 vs. P30 | 0.809 | 0.421 | Age F(3,47.30)=8.14, p=0.0002 | P21 vs. P30 | 4.740 | 0.00001 |
| | | | P24 vs. P30 | -1.244 | 0.218 | Body Length F(1,45.13)=30.25, p=1.7E-06 | P24 vs. P30 | 1.970 | 0.053 |
| | | | P27 vs. P30 | 0.859 | 0.395 | | P27 vs. P30 | 2.622 | 0.011 |

| | | | | | | | | | |
|---|---|---|---|---|---|---|---|---|---|
| Stance Duration Fore | normal | Age F(3,46.76)=16.38, p=2.1E-07 | P21 vs. P30 | -6.450 | 1.2E-08 | Age F(3,41.75)=3.73, p=0.018 | P21 vs. P30 | -1.211 | 0.231 |
| | | | P24 vs. P30 | -2.263 | 0.027 | Body Length F(1,53.19)=21.31, p=2.5E-05 | P24 vs. P30 | 0.787 | 0.434 |
| | | | P27 vs. P30 | -0.177 | 0.860 | | P27 vs. P30 | 1.647 | 0.105 |
| Stance Duration Hind | normal | Age F(3,46.45)=8.60, p=0.0001 | P21 vs. P30 | -4.818 | 8.1E-06 | Age F(3,47.55)=3.44, p=0.024 | P21 vs. P30 | 2.969 | 0.004 |
| | | | P24 vs. P30 | -3.139 | 0.003 | Body Length F(1,45.45)=107.42, p=1.5E-13 | P24 vs. P30 | 2.220 | 0.030 |
| | | | P27 vs. P30 | -0.879 | 0.384 | | P27 vs. P30 | 2.554 | 0.014 |
| Stance Factor Fore | normal | Age F(3,50.18)=0.33, p=0.801 | P21 vs. P30 | -0.010 | 0.992 | Age F(3,45.10)=0.43, p=0.734 | P21 vs. P30 | -0.700 | 0.487 |
| | | | P24 vs. P30 | 0.158 | 0.875 | Body Length F(1,52.82)=0.89, p=0.350 | P24 vs. P30 | -0.413 | 0.681 |
| | | | P27 vs. P30 | -0.763 | 0.449 | | P27 vs. P30 | -1.030 | 0.307 |
| Stance Factor Hind | normal | Age F(3,55.90)=2.53, p=0.07 | P21 vs. P30 | 1.379 | 0.172 | Age F(3,48.01)=2.58, p=0.065 | P21 vs. P30 | 1.159 | 0.251 |
| | | | P24 vs. P30 | -0.773 | 0.442 | Body Length F(1,52.26)=0.120, p=0.742 | P24 vs. P30 | -0.438 | 0.663 |
| | | | P27 vs. P30 | 1.607 | 0.114 | | P27 vs. P30 | 1.631 | 0.108 |
| Stance Width CV Fore | normal | Age F(3,51.98)=0.83, p=0.48 | P21 vs. P30 | 0.801 | 0.426 | Age F(3,47.64)=0.87, p=0.462 | P21 vs. P30 | 0.961 | 0.340 |
| | | | P24 vs. P30 | 1.167 | 0.247 | Body Length F(1,55.14)=0.330, p=0.567 | P24 vs. P30 | 1.281 | 0.205 |
| | | | P27 vs. P30 | -0.231 | 0.819 | | P27 vs. P30 | -0.003 | 0.997 |
| Stance Width CV Hind | P27 nonnormal | Age F(3,59.17)=3.69, p=0.017 | P21 vs. P30 | 0.572 | 0.569 | Age F(3,52.89)=3.35, p=0.026 | P21 vs. P30 | -0.728 | 0.469 |
| | | | P24 vs. P30 | 3.192 | 0.002 | Body Length F(1,54.95)=2.19, p=0.145 | P24 vs. P30 | 1.795 | 0.077 |
| | | | P27 vs. P30 | 1.490 | 0.142 | | P27 vs. P30 | 0.931 | 0.355 |
| Stance Width Fore | normal | Age F(3,44.84)=3.06, p=0.038 | P21 vs. P30 | 1.519 | 0.133 | Age F(3,41.53)=3.57, p=0.022 | P21 vs. P30 | 1.841 | 0.070 |
| | | | P24 vs. P30 | -1.209 | 0.231 | Body Length F(1,61.57)=1.27, p=0.263 | P24 vs. P30 | -0.201 | 0.842 |
| | | | P27 vs. P30 | -0.784 | 0.437 | | P27 vs. P30 | -0.180 | 0.858 |
| Stance Width Hind | normal | Age F(3,46.90)=6.04, p=0.001 | P21 vs. P30 | -0.310 | 0.758 | Age F(3,44.49)=8.58, p=0.0001 | P21 vs. P30 | 2.598 | 0.012 |
| | | | P24 vs. P30 | -3.635 | 0.001 | Body Length F(1,61.09)=13.01, p=0.0006 | P24 vs. P30 | -0.732 | 0.467 |
| | | | P27 vs. P30 | -1.992 | 0.052 | | P27 vs. P30 | -0.328 | 0.744 |
| Step Angle CV Fore | normal | Age F(3,52.06)=2.91, p=0.043 | P21 vs. P30 | 2.641 | 0.010 | Age F(3,48.07)=0.327, p=0.806 | P21 vs. P30 | 0.497 | 0.621 |
| | | | P24 vs. P30 | 2.424 | 0.018 | Body Length F(1,55.37)=2.74, p=0.104 | P24 vs. P30 | 0.954 | 0.343 |
| | | | P27 vs. P30 | 1.168 | 0.248 | | P27 vs. P30 | 0.470 | 0.640 |
| Step Angle CV Hind | normal | Age F(3,54.33)=0.15, p=0.93 | P21 vs. P30 | 0.401 | 0.690 | Age F(3,41.97)=0.105, p=0.956 | P21 vs. P30 | 0.319 | 0.751 |
| | | | P24 vs. P30 | 0.452 | 0.653 | Body Length F(1,44.06)=0.004, p=0.949 | P24 vs. P30 | 0.407 | 0.686 |
| | | | P27 vs. P30 | -0.100 | 0.920 | | P27 vs. P30 | -0.076 | 0.939 |
| Step Angle Fore | normal | Age F(3,52.34)=3.34, p=0.026 | P21 vs. P30 | -2.952 | 0.004 | Age F(3,48.04)=0.993, p=0.404 | P21 vs. P30 | -0.672 | 0.504 |
| | | | P24 vs. P30 | -1.526 | 0.132 | Body Length F(1,55.61)=2.89, p=0.095 | P24 vs. P30 | -0.228 | 0.820 |
| | | | P27 vs. P30 | -2.197 | 0.033 | | P27 vs. P30 | -1.464 | 0.148 |
| Step Angle Hind | normal | Age F(3,56.09)=1.14, p=0.34 | P21 vs. P30 | -1.602 | 0.114 | Age F(3,47.17)=0.87, p=0.464 | P21 vs. P30 | -1.148 | 0.255 |
| | | | P24 vs. P30 | 0.110 | 0.912 | Body Length F(1,50.23)=0.011, p=0.916 | P24 vs. P30 | 0.030 | 0.976 |
| | | | P27 vs. P30 | -0.590 | 0.558 | | P27 vs. P30 | -0.593 | 0.556 |
| Stride Frequency Fore | normal | Age F(3,49.42)=7.26, p=0.0004 | P21 vs. P30 | 4.360 | 4.4E-05 | Age F(3,45.34)=1.004, p=0.400 | P21 vs. P30 | -1.476 | 0.145 |
| | | | P24 vs. P30 | 2.847 | 0.006 | Body Length F(1,50.29)=46.51, p=1.1E-08 | P24 vs. P30 | -1.075 | 0.286 |
| | | | P27 vs. P30 | 0.771 | 0.444 | | P27 vs. P30 | -1.517 | 0.135 |
| Stride Frequency Hind | normal | Age F(3,50.22)=6.32, p=0.001 | P21 vs. P30 | 4.033 | 0.0001 | Age F(3,45.78)=0.997, p=0.403 | P21 vs. P30 | -1.661 | 0.102 |
| | | | P24 vs. P30 | 2.785 | 0.007 | Body Length F(1,45.90)=47.43, p=1.4E-08 | P24 vs. P30 | -0.956 | 0.343 |
| | | | P27 vs. P30 | 0.862 | 0.393 | | P27 vs. P30 | -1.179 | 0.244 |
| Stride Length CV Fore | normal | Age F(3,57.24)=1.74, p=0.169 | P21 vs. P30 | 1.650 | 0.103 | Age F(3,48.07)=1.40, p=0.254 | P21 vs. P30 | 1.534 | 0.130 |
| | | | P24 vs. P30 | 2.013 | 0.048 | Body Length F(1,49.80)=0.33, p=0.570 | P24 vs. P30 | 1.981 | 0.052 |
| | | | P27 vs. P30 | 0.637 | 0.527 | | P27 vs. P30 | 0.789 | 0.434 |
| Stride Length CV Hind | normal | Age F(3,45.95)=1.64, p=0.194 | P21 vs. P30 | -0.774 | 0.441 | Age F(3,42.26)=3.13, p=0.035 | P21 vs. P30 | 1.830 | 0.072 |
| | | | P24 vs. P30 | 1.337 | 0.186 | Body Length F(1,54.39)=9.34, p=0.003 | P24 vs. P30 | 3.004 | 0.004 |
| | | | P27 vs. P30 | 0.816 | 0.419 | | P27 vs. P30 | 1.985 | 0.052 |
| Stride Length Fore | normal | Age F(3,49.58)=8.02, p=0.0002 | P21 vs. P30 | -4.458 | 0.00003 | Age F(3,44.89)=1.16, p=0.334 | P21 vs. P30 | 1.408 | 0.164 |
| | | | P24 vs. P30 | -2.963 | 0.004 | Body Length F(1,48.47)=48.38, p=8.2E-09 | P24 vs. P30 | 0.950 | 0.346 |
| | | | P27 vs. P30 | -0.558 | 0.580 | | P27 vs. P30 | 1.717 | 0.092 |
| Stride Length Hind | normal | Age F(3,50.57)=6.66, p=0.0007 | P21 vs. P30 | -4.090 | 0.0001 | Age F(3,46.64)=0.994, p=0.404 | P21 vs. P30 | 1.604 | 0.114 |
| | | | P24 vs. P30 | -3.040 | 0.003 | Body Length F(1,46.46)=47.17, p=1.46E-08 | P24 vs. P30 | 0.690 | 0.493 |
| | | | P27 vs. P30 | -0.948 | 0.348 | | P27 vs. P30 | 1.085 | 0.283 |
| Swing Duration CV Fore | normal | Age F(3,61.32)=1.28, p=0.29 | P21 vs. P30 | 0.527 | 0.600 | Age F(3,49.61)=1.09, p=0.361 | P21 vs. P30 | 0.027 | 0.978 |
| | | | P24 vs. P30 | 1.837 | 0.070 | Body Length F(1,46.37)=0.212, p=0.648 | P24 vs. P30 | 1.271 | 0.208 |
| | | | P27 vs. P30 | 1.316 | 0.195 | | P27 vs. P30 | 1.133 | 0.262 |
| Swing Duration CV Hind | P30 nonnormal | Age F(3,45.40)=0.17, p=0.91 | P21 vs. P30 | -0.497 | 0.621 | Age F(3,43.70)=0.48, p=0.700 | P21 vs. P30 | 1.024 | 0.310 |
| | | | P24 vs. P30 | -0.469 | 0.640 | Body Length F(1,56.28)=3.05, p=0.086 | P24 vs. P30 | 0.704 | 0.484 |
| | | | P27 vs. P30 | -0.680 | 0.500 | | P27 vs. P30 | 0.062 | 0.951 |
| Swing Duration Fore | normal | Age F(3,44.57)=2.24, p=0.10 | P21 vs. P30 | -0.377 | 0.707 | Age F(3,42.33)=5.46, p=0.003 | P21 vs. P30 | 2.967 | 0.004 |
| | | | P24 vs. P30 | -2.271 | 0.027 | Body Length F(1,66.75)=17.00, p=0.0001 | P24 vs. P30 | 0.832 | 0.408 |
| | | | P27 vs. P30 | -1.001 | 0.322 | | P27 vs. P30 | 1.018 | 0.313 |
| Swing Duration Hind | normal | Age F(3,47.86)=0.80, p=0.50 | P21 vs. P30 | -1.095 | 0.277 | Age F(3,38.42)=0.265, p=0.850 | P21 vs. P30 | 0.003 | 0.998 |
| | | | P24 vs. P30 | -1.469 | 0.146 | Body Length F(1,48.81)=0.92, p=0.342 | P24 vs. P30 | -0.581 | 0.563 |
| | | | P27 vs. P30 | -0.952 | 0.347 | | P27 vs. P30 | -0.506 | 0.615 |

# Supplementary Table 2. Statistical output for FVB gait data.

| OUTCOME | NORMAL | LMM | estimates | *t* | sig. | LMM (adjusted for body length) | estimates | *t* | sig. |
|---|---|---|---|---|---|---|---|---|---|
| %Hindlimb Shared Stance | normal | Age F(3,48.51)=7.58, p=0.0003 | P21 vs. P30 | -4.421 | 0.00003 | Age F(3,48.981)=7.219 p=.0004 | P21 vs. P30 | -4.152 | 0.00009 |
| | | | P24 vs. P30 | -2.772 | 0.007 | Body Length F(1,75.261)=1.732, p=.192 | P24 vs. P30 | -3.070 | 0.003 |
| | | | P27 vs. P30 | -0.573 | 0.569 | | P27 vs. P30 | -1.191 | 0.238 |
| % Stance Fore | normal | Age F(3,52.07)=2.03, p=0.121 | P21 vs. P30 | -1.728 | 0.088 | Age F(3,51.440)=1.352 p=.267 | P21 vs. P30 | 0.508 | 0.613 |
| | | | P24 vs. P30 | 0.335 | 0.739 | Body Length F(1,64.023)=7.224, p=.009 | P24 vs. P30 | 1.708 | 0.092 |
| | | | P27 vs. P30 | -0.473 | 0.638 | | P27 vs. P30 | 0.751 | 0.456 |
| % Stance Hind | normal | Age F(3,50.01)=9.93, p=3.04E-05 | P21 vs. P30 | -4.571 | 0.00002 | Age F(3,50.437)=10.498 p=.00002 | P21 vs. P30 | -4.520 | 0.00002 |
| | | | P24 vs. P30 | -2.698 | 0.009 | Body Length F(1,74.647)=2.737, p=.102 | P24 vs. P30 | -3.212 | 0.002 |
| | | | P27 vs. P30 | 0.357 | 0.722 | | P27 vs. P30 | -0.565 | 0.574 |
| % Swing Fore | normal | Age F(3,52.07)=2.03, p=0.121 | P21 vs. P30 | 1.728 | 0.088 | Age F(3,51.440)=1.352 p=.267 | P21 vs. P30 | -0.508 | 0.613 |
| | | | P24 vs. P30 | -0.335 | 0.739 | Body Length F(1,64.023)=7.224, p=.009 | P24 vs. P30 | -1.708 | 0.092 |
| | | | P27 vs. P30 | 0.473 | 0.638 | | P27 vs. P30 | -0.751 | 0.456 |
| % Swing Hind | normal | Age F(3,50.0)=9.93, p=3.0E-05 | P21 vs. P30 | 4.571 | 0.00002 | Age F(3,50.437)=10.498 p=.00002 | P21 vs. P30 | 4.520 | 0.00002 |
| | | | P24 vs. P30 | 2.698 | 0.009 | Body Length F(1,74.647)=2.737, p=.102 | P24 vs. P30 | 3.212 | 0.002 |
| | | | P27 vs. P30 | -0.357 | 0.722 | | P27 vs. P30 | 0.565 | 0.574 |
| Absolute Paw Angle Fore | P21 nonnormal | Age F(3,42.23)=4.18, p=0.011 | P21 vs. P30 | 2.760 | 0.007 | Age F(3,42.074)=2.751 p=.054 | P21 vs. P30 | 1.627 | 0.108 |
| | | Friedman's Test: | P24 vs. P30 | -0.214 | 0.831 | Body Length F(1,56.325)=0.343, p=.560 | P24 vs. P30 | -0.489 | 0.626 |
| | | χ2(3,N=19)=5.286, p=.152 | P27 vs. P30 | 0.672 | 0.506 | | P27 vs. P30 | 0.351 | 0.727 |
| Absolute Paw Angle Hind | normal | Age F(3,52.72)=0.67, p=0.58 | P21 vs. P30 | 1.288 | 0.202 | Age F(3,53.529)=1.557 p=.211 | P21 vs. P30 | 2.070 | 0.042 |
| | | | P24 vs. P30 | 0.941 | 0.350 | Body Length F(167.102)=2.662, p=.107 | P24 vs. P30 | 1.677 | 0.098 |
| | | | P27 vs. P30 | 1.116 | 0.270 | | P27 vs. P30 | 1.757 | 0.084 |
| Brake Duration Fore | normal | Age F(3,52.29)=0.89, p=0.45 | P21 vs. P30 | -1.266 | 0.209 | Age F(3,52.673)=0.496 p=.687 | P21 vs. P30 | -0.016 | 0.987 |
| | | Friedman's Test: | P24 vs. P30 | -0.472 | 0.638 | Body Length F(1,74.065)=1.745, p=.191 | P24 vs. P30 | 0.321 | 0.749 |
| | | χ2(3,N=19)=6.206, p=.102 | P27 vs. P30 | 0.321 | 0.749 | | P27 vs. P30 | 0.934 | 0.354 |
| Brake Duration Hind | normal | Age F(3,52.26)=4.56, p=0.007 | P21 vs. P30 | -3.386 | 0.001 | Age F(3,52.177)=2.801 p=.049 | P21 vs. P30 | -2.404 | 0.019 |
| | | | P24 vs. P30 | -0.831 | 0.408 | Body Length F(1,65.993)=0.009, p=.924 | P24 vs. P30 | -0.656 | 0.514 |
| | | | P27 vs. P30 | -1.177 | 0.245 | | P27 vs. P30 | -1.010 | 0.316 |
| Gait Symmetry | P21, P24, P27, nonnormal | Age F(3,58.11)=0.577, p=0.632 | P21 vs. P30 | 0.862 | 0.391 | Age F(3,58.383)=0.381 p=.767 | P21 vs. P30 | -0.275 | 0.784 |
| | | Friedman's Test: | P24 vs. P30 | 0.862 | 0.391 | Body Length F(1,59.600)=1.976, p=.165 | P24 vs. P30 | 0.058 | 0.954 |
| | | χ2(3,N=19)=2.692, p=.442 | P27 vs. P30 | 1.277 | 0.208 | | P27 vs. P30 | 0.657 | 0.513 |
| Max. Rate Contact Change Fore | normal | Age F(3,48.74)=8.45, p=0.0001 | P21 vs. P30 | -2.513 | 0.014 | Age F(3,48.024)=8.273 p=.0002 | P21 vs. P30 | -2.462 | 0.016 |
| | | | P24 vs. P30 | -3.877 | 0.0002 | Body Length F(1,73.296)=0.823, p=.367 | P24 vs. P30 | -3.760 | 0.0003 |
| | | | P27 vs. P30 | -4.896 | 0.00001 | | P27 vs. P30 | -4.723 | 0.00002 |
| Max. Rate Contact Change Hind | normal | Age F(3,47.67)=10.09, p=3.0E-05 | P21 vs. P30 | -5.121 | 2.25E-06 | Age F(3,48.107)=10.984 p=.00001 | P21 vs. P30 | -5.344 | 9.47E-07 |
| | | | P24 vs. P30 | -5.002 | 4.03E-06 | Body Length F(1,72.083)=4.890, p=.030 | P24 vs. P30 | -5.591 | 4.21E-07 |
| | | | P27 vs. P30 | -3.606 | 0.0007 | | P27 vs. P30 | -4.489 | 0.00004 |
| Paw Angle CV Fore | P21, P24, P30 nonnormal | Age F(3,41.66)=2.56, p=0.07 | P21 vs. P30 | -2.317 | 0.023 | Age F(3,42.031)=2.046 p=.122 | P21 vs. P30 | -1.953 | 0.055 |
| | | Friedman's Test: | P24 vs. P30 | -0.976 | 0.332 | Body Length F(1,57.606)=0.142, p=.707 | P24 vs. P30 | -1.030 | 0.306 |
| | | χ2(3,N=19)=7.926, p=.048 | P27 vs. P30 | -2.116 | 0.041 | | P27 vs. P30 | -2.068 | 0.044 |
| Paw Angle CV Hind | P24, P30 nonnormal | Age F(3,51.84)=3.67, p=0.018 | P21 vs. P30 | 2.833 | 0.006 | Age F(3,52.626)=1.543 p=.214 | P21 vs. P30 | 0.724 | 0.472 |
| | | Friedman's Test: | P24 vs. P30 | 1.179 | 0.242 | Body Length F(1,69.880)=4.084, p=.047 | P24 vs. P30 | -0.063 | 0.950 |
| | | χ2(3,N=19)=5.716, p=.126 | P27 vs. P30 | -0.217 | 0.829 | | P27 vs. P30 | -1.142 | 0.258 |
| Paw Overlap Distance | normal | Age F(3,55.98)=33.05, p=2.0E-12 | P21 vs. P30 | -9.557 | 1.16E-14 | Age F(3,54.359)=12.790 p=1.9E-06 | P21 vs. P30 | -5.079 | 2.68E-06 |
| | | | P24 vs. P30 | -5.105 | 2.69E-06 | Body Length F(1,73.109)=13.448, | P24 vs. P30 | -2.534 | 0.013 |
| | | | P27 vs. P30 | -3.383 | 0.001 | p=.0005 | P27 vs. P30 | -1.141 | 0.258 |
| Paw Placement Positioning | normal | Age F(3,52.59)=0.87, p=0.463 | P21 vs. P30 | -1.062 | 0.291 | Age F(3,52.296)=0.416 p=.742 | P21 vs. P30 | 0.018 | 0.986 |
| | | | P24 vs. P30 | -1.219 | 0.227 | Body Length F(1,59.145)=1.449, p=.233 | P24 vs. P30 | -0.440 | 0.661 |
| | | | P27 vs. P30 | 0.036 | 0.971 | | P27 vs. P30 | 0.529 | 0.599 |
| Peak Paw Area CV Fore | normal | Age F(3,52.12)=6.06, p=0.001 | P21 vs. P30 | 4.040 | 0.0001 | Age F(3,49.542)=1.128 p=.347 | P21 vs. P30 | 1.030 | 0.306 |
| | | | P24 vs. P30 | 1.635 | 0.106 | Body Length F(1,68.060)=9.369, p=.003 | P24 vs. P30 | -0.194 | 0.847 |
| | | | P27 vs. P30 | 1.436 | 0.157 | | P27 vs. P30 | -0.143 | 0.887 |
| Peak Paw Area CV Hind | normal | Age F(3,55.29)=31.80, p=4.4E-12 | P21 vs. P30 | 9.044 | 1.33E-13 | Age F(3,54.239)=15.958 p=1.5E-07 | P21 vs. P30 | 5.344 | 1.01E-06 |
| | | | P24 vs. P30 | 4.547 | 0.00002 | Body Length F(1,69.664)=4.445, p=.039 | P24 vs. P30 | 2.806 | 0.006 |
| | | | P27 vs. P30 | 0.909 | 0.367 | | P27 vs. P30 | -0.168 | 0.867 |
| Peak Paw Area Fore | normal | Age F(3,48.81)=8.80, p=0.00009 | P21 vs. P30 | -1.251 | 0.215 | Age F(3,47.398)=9.342 p=.00006 | P21 vs. P30 | -1.540 | 0.128 |
| | | | P24 vs. P30 | -3.034 | 0.003 | Body Length F(1,69.947)=0.824, p=.367 | P24 vs. P30 | -3.056 | 0.003 |
| | | | P27 vs. P30 | -4.880 | 0.00001 | | P27 vs. P30 | -4.698 | 0.00002 |
| Peak Paw Area Hind | normal | Age F(3,48.69)=5.51, p=0.002 | P21 vs. P30 | -3.558 | 0.001 | Age F(3,48.118)=6.291 p=.001 | P21 vs. P30 | -3.862 | 0.0002 |
| | | | P24 vs. P30 | -3.668 | 0.0005 | Body Length F(1,73.124)=3.208, p=.077 | P24 vs. P30 | -4.149 | 0.00009 |
| | | | P27 vs. P30 | -3.220 | 0.002 | | P27 vs. P30 | -3.846 | 0.0003 |
| Propulsion Duration Fore | normal | Age F(3,51.83)=5.31, p=0.003 | P21 vs. P30 | -1.631 | 0.107 | Age F(3,46.904)=3.710 p=.017 | P21 vs. P30 | 1.299 | 0.198 |
| | | | P24 vs. P30 | -1.146 | 0.256 | Body Length F(1,73.391)=14.104, | P24 vs. P30 | 1.042 | 0.301 |
| | | | P27 vs. P30 | -3.622 | 0.001 | p=.0003 | P27 vs. P30 | -1.315 | 0.194 |
| Propulsion Duration Hind | normal | Age F(3,54.22)=5.86, p=0.002 | P21 vs. P30 | -3.821 | 0.0003 | Age F(3,50.093)=0.853 p=.471 | P21 vs. P30 | -1.176 | 0.243 |
| | | | P24 vs. P30 | -3.356 | 0.001 | Body Length F(1,74.708)=6.166, p=.015 | P24 vs. P30 | -1.428 | 0.158 |
| | | | P27 vs. P30 | -3.398 | 0.001 | | P27 vs. P30 | -1.538 | 0.129 |
| Stance Duration Fore | normal | Age F(3,54.86)=5.25, p=0.003 | P21 vs. P30 | -2.925 | 0.005 | Age F(3,50.573)=4.218 p=.010 | P21 vs. P30 | 1.233 | 0.221 |
| | | | P24 vs. P30 | -1.807 | 0.075 | Body Length F(1,74.488)=29.772, | P24 vs. P30 | 1.330 | 0.188 |
| | | | P27 vs. P30 | -4.243 | 0.00009 | p=6.1E-07 | P27 vs. P30 | -1.173 | 0.246 |

| | | | | | | | | | |
|---|---|---|---|---|---|---|---|---|---|
| Stance Duration Hind | normal | Age F(3,53.20)=0.96, p=0.417 | P21 vs. P30 | -6.315 | 1.69E-08 | Age F(3,49.505)=2.862 p=.046 | P21 vs. P30 | -2.644 | 0.010 |
| | | | P24 vs. P30 | -4.397 | 0.00004 | Body Length F(1,74.508)=10.501, | P24 vs. P30 | -1.947 | 0.055 |
| | | | P27 vs. P30 | -4.857 | 0.00001 | p=.002 | P27 vs. P30 | -2.420 | 0.019 |
| Stance Factor Fore | normal | Age F(3,53.72)=8.14, p=0.0001 | P21 vs. P30 | 1.796 | 0.077 | Age F(3,54.845)=4.700 p=.005 | P21 vs. P30 | 0.868 | 0.389 |
| | | | P24 vs. P30 | -1.315 | 0.192 | Body Length F(1,65.043)=0.438, p=.511 | P24 vs. P30 | -1.471 | 0.146 |
| | | | P27 vs. P30 | -1.792 | 0.079 | | P27 vs. P30 | -1.908 | 0.061 |
| Stance Factor Hind | normal | Age F(3,54.13)=15.43, p=2.3E-07 | P21 vs. P30 | 1.472 | 0.145 | Age F(3,53.181)=1.486 p=.229 | P21 vs. P30 | 1.925 | 0.058 |
| | | | P24 vs. P30 | 0.304 | 0.762 | Body Length F(1,70.251)=1.516, p=.222 | P24 vs. P30 | 0.920 | 0.360 |
| | | | P27 vs. P30 | 0.148 | 0.883 | | P27 vs. P30 | 0.710 | 0.481 |
| Stance Width CV Fore | normal | Age F(3,54.47)=0.21, p=0.888 | P21 vs. P30 | -0.608 | 0.545 | Age F(3,56.373)=0.875 p=.460 | P21 vs. P30 | -1.463 | 0.148 |
| | | | P24 vs. P30 | -0.708 | 0.481 | Body Length F(1,51.300)=2.402, p=.127 | P24 vs. P30 | -1.376 | 0.173 |
| | | | P27 vs. P30 | -0.314 | 0.755 | | P27 vs. P30 | -0.829 | 0.411 |
| Stance Width CV Hind | normal | Age F(3,52.76)=1.34, p=0.271 | P21 vs. P30 | 1.632 | 0.107 | Age F(3,52.867)=0.354 p=.786 | P21 vs. P30 | 0.012 | 0.990 |
| | | | P24 vs. P30 | 0.879 | 0.382 | Body Length F(1,67.868)=3.085, p=.084 | P24 vs. P30 | -0.177 | 0.860 |
| | | | P27 vs. P30 | 1.624 | 0.111 | | P27 vs. P30 | 0.641 | 0.524 |
| Stance Width Fore | normal | Age F(3,51.90)=0.231, p=0.874 | P21 vs. P30 | 0.730 | 0.468 | Age F(3,47.735)=.186 p=.906 | P21 vs. P30 | -0.453 | 0.652 |
| | | | P24 vs. P30 | 0.401 | 0.689 | Body Length F(1,75.985)=2.070, p=.154 | P24 vs. P30 | -0.465 | 0.643 |
| | | | P27 vs. P30 | 0.000 | 1.000 | | P27 vs. P30 | -0.739 | 0.463 |
| Stance Width Hind | normal | Age F(3,54.81)=0.990, p=0.405 | P21 vs. P30 | -0.637 | 0.526 | Age F(3,55.454)=1.147 p=.338 | P21 vs. P30 | -0.940 | 0.350 |
| | | | P24 vs. P30 | -1.498 | 0.138 | Body Length F(1,75.475)=.480, p=.491 | P24 vs. P30 | -1.635 | 0.106 |
| | | | P27 vs. P30 | -0.329 | 0.744 | | P27 vs. P30 | -0.632 | 0.529 |
| Step Angle CV Fore | normal | Age F(3,51.71)=0.21, p=0.890 | P21 vs. P30 | -0.717 | 0.476 | Age F(3,52.059)=1.150 p=.338 | P21 vs. P30 | -1.826 | 0.072 |
| | | | P24 vs. P30 | -0.448 | 0.656 | Body Length F(1,63.293)=3.590, p=.063 | P24 vs. P30 | -1.380 | 0.172 |
| | | | P27 vs. P30 | -0.082 | 0.935 | | P27 vs. P30 | -0.901 | 0.371 |
| Step Angle CV Hind | normal | Age F(3,52.19)=2.60, p=0.06 | P21 vs. P30 | 1.221 | 0.226 | Age F(3,51.063)=2.550 p=.066 | P21 vs. P30 | -0.360 | 0.720 |
| | | | P24 vs. P30 | 1.442 | 0.154 | Body Length F(1,75.767)=3.385, p=.070 | P24 vs. P30 | 0.202 | 0.841 |
| | | | P27 vs. P30 | -0.954 | 0.345 | | P27 vs. P30 | -1.757 | 0.084 |
| Step Angle Fore | normal | Age F(3,51.25)=0.18, p=0.912 | P21 vs. P30 | -0.667 | 0.507 | Age F(3,52.446)=0.159 p=.923 | P21 vs. P30 | 0.511 | 0.611 |
| | | | P24 vs. P30 | -0.114 | 0.910 | Body Length F(1,56.512)=2.310, p=.134 | P24 vs. P30 | 0.666 | 0.507 |
| | | | P27 vs. P30 | -0.323 | 0.748 | | P27 vs. P30 | 0.305 | 0.761 |
| Step Angle Hind | normal | Age F(3,53.08)=0.15, p=0.929 | P21 vs. P30 | -0.263 | 0.793 | Age F(3,58.741)=0.316 p=.814 | P21 vs. P30 | 0.767 | 0.446 |
| | | | P24 vs. P30 | -0.556 | 0.580 | Body Length F(1,51.207)=2.250, p=.140 | P24 vs. P30 | 0.234 | 0.815 |
| | | | P27 vs. P30 | -0.578 | 0.566 | | P27 vs. P30 | 0.005 | 0.996 |
| Stride Frequency Fore | normal | Age F(3,54.42)=7.07, p=0.0004 | P21 vs. P30 | 2.439 | 0.017 | Age F(3,52.105)=4.336 p=.008 | P21 vs. P30 | -1.392 | 0.168 |
| | | | P24 vs. P30 | 1.707 | 0.092 | Body Length F(1,75.998)=24.818, | P24 vs. P30 | -1.174 | 0.244 |
| | | | P27 vs. P30 | 4.168 | 0.0001 | p=3.6E-06 | P27 vs. P30 | 1.324 | 0.190 |
| Stride Frequency Hind | normal | Age F(3,54.09)=7.02, p=0.0005 | P21 vs. P30 | 2.330 | 0.022 | Age F(3,50.760)=4.136 p=.010 | P21 vs. P30 | -1.379 | 0.172 |
| | | | P24 vs. P30 | 1.541 | 0.128 | Body Length F(1,75.608)=23.107, | P24 vs. P30 | -1.268 | 0.209 |
| | | | P27 vs. P30 | 4.061 | 0.0002 | p=.000008 | P27 vs. P30 | 1.196 | 0.236 |
| Stride Length CV Fore | normal | Age F(3,57.11)=1.73, p=0.170 | P21 vs. P30 | -1.677 | 0.098 | Age F(3,56.683)=1.671 p=.183 | P21 vs. P30 | -1.681 | 0.097 |
| | | | P24 vs. P30 | -1.605 | 0.113 | Body Length F(1,61.257)=0.419, p=.520 | P24 vs. P30 | -1.716 | 0.090 |
| | | | P27 vs. P30 | -0.030 | 0.976 | | P27 vs. P30 | -0.289 | 0.773 |
| Stride Length CV Hind | normal | Age F(3,54.92)=0.478, p=0.700 | P21 vs. P30 | -0.434 | 0.665 | Age F(3,54.683)=0.788 p=.506 | P21 vs. P30 | -1.097 | 0.276 |
| | | | P24 vs. P30 | 0.640 | 0.524 | Body Length F(1,61.420)=1.317, p=.256 | P24 vs. P30 | -0.033 | 0.973 |
| | | | P27 vs. P30 | -0.218 | 0.828 | | P27 vs. P30 | -0.677 | 0.501 |
| Stride Length Fore | normal | Age F(3,53.60)=7.32, p=0.0003 | P21 vs. P30 | -2.521 | 0.014 | Age F(3,50.170)=4.024 p=.012 | P21 vs. P30 | 1.176 | 0.243 |
| | | | P24 vs. P30 | -2.098 | 0.040 | Body Length F(1,75.959)=22.304, | P24 vs. P30 | 0.708 | 0.481 |
| | | | P27 vs. P30 | -4.411 | 0.00005 | p=.00001 | P27 vs. P30 | -1.570 | 0.122 |
| Stride Length Hind | normal | Age F(3,53.58)=8.19, p=0.0001 | P21 vs. P30 | -2.696 | 0.009 | Age F(3,49.715)=4.110 p=.011 | P21 vs. P30 | 0.908 | 0.367 |
| | | | P24 vs. P30 | -2.102 | 0.039 | Body Length F(1,75.693)=20.333, | P24 vs. P30 | 0.618 | 0.538 |
| | | | P27 vs. P30 | -4.582 | 0.00003 | p=.00002 | P27 vs. P30 | -1.752 | 0.085 |
| Swing Duration CV Fore | normal | Age F(3,51.78)=1.36, p=0.266 | P21 vs. P30 | -1.708 | 0.092 | Age F(3,51.110)=1.902 p=.141 | P21 vs. P30 | -2.117 | 0.038 |
| | | | P24 vs. P30 | -0.654 | 0.515 | Body Length F(1,58.880)=1.552, p=.218 | P24 vs. P30 | -1.205 | 0.232 |
| | | | P27 vs. P30 | 0.137 | 0.891 | | P27 vs. P30 | -0.395 | 0.695 |
| Swing Duration CV Hind | normal | Age F(3,50.61)=2.77, p=0.051 | P21 vs. P30 | 2.590 | 0.012 | Age F(3,50.603)=1.306 p=.283 | P21 vs. P30 | 1.496 | 0.139 |
| | | | P24 vs. P30 | 1.166 | 0.247 | Body Length F(1,58.395)=0.389, p=.535 | P24 vs. P30 | 0.683 | 0.497 |
| | | | P27 vs. P30 | 0.140 | 0.889 | | P27 vs. P30 | -0.132 | 0.895 |
| Swing Duration Fore | normal | Age F(3,52.81)=3.36, p=0.026 | P21 vs. P30 | -1.332 | 0.187 | Age F(3,51.581)=2.728 p=.053 | P21 vs. P30 | 1.247 | 0.216 |
| | | | P24 vs. P30 | -1.779 | 0.079 | Body Length F(1,69.908)=11.086, | P24 vs. P30 | 0.213 | 0.832 |
| | | | P27 vs. P30 | -3.169 | 0.003 | p=.001 | P27 vs. P30 | -1.248 | 0.217 |
| Swing Duration Hind | normal | Age F(3,50.31)=5.15, p=0.003 | P21 vs. P30 | 1.740 | 0.086 | Age F(3,48.861)=8.474 p=.0001 | P21 vs. P30 | 3.616 | 0.001 |
| | | | P24 vs. P30 | 0.786 | 0.435 | Body Length F(1,75.690)=10.861, | P24 vs. P30 | 2.532 | 0.014 |
| | | | P27 vs. P30 | -2.118 | 0.039 | p=.002 | P27 vs. P30 | -0.183 | 0.856 |

**Supplementary Table 3. Descriptive statistics for C57 and FVB gait data.**

| Outcome | Age | C57 Unadjusted | | C57 Adjusted | | FVB Unadjusted | | FVB Adjusted | |
|---|---|---|---|---|---|---|---|---|---|
| | | Mean | *SD* | Mean | *SD* | Mean | *SD* | Mean | *SD* |
| % Hindlimb Shared Stance | P21 | 39.599 | 7.930 | 43.680 | 9.643 | 43.434 | 8.596 | 42.158 | 9.890 |
| | P24 | 43.111 | 6.970 | 44.521 | 6.620 | 47.618 | 8.596 | 47.295 | 8.707 |
| | P27 | 43.222 | 6.564 | 42.559 | 6.113 | 52.308 | 8.596 | 52.460 | 8.639 |
| | P30 | 43.814 | 6.335 | 40.508 | 7.224 | 53.242 | 8.596 | 54.690 | 10.226 |
| % Stance Fore | P21 | 57.831 | 4.257 | 56.801 | 5.695 | 59.492 | 1.686 | 60.022 | 1.880 |
| | P24 | 61.785 | 3.766 | 61.461 | 3.937 | 60.466 | 1.686 | 60.600 | 1.619 |
| | P27 | 61.600 | 3.548 | 61.787 | 3.599 | 60.113 | 1.686 | 60.050 | 1.604 |
| | P30 | 60.992 | 3.452 | 61.759 | 4.433 | 60.311 | 1.686 | 59.709 | 1.952 |
| % Stance Hind | P21 | 62.893 | 3.078 | 64.582 | 3.734 | 64.842 | 3.320 | 64.237 | 3.782 |
| | P24 | 64.041 | 2.714 | 64.601 | 2.556 | 66.632 | 3.320 | 66.478 | 3.343 |
| | P27 | 64.724 | 2.556 | 64.467 | 2.356 | 68.921 | 3.320 | 68.993 | 3.318 |
| | P30 | 64.723 | 2.478 | 63.406 | 2.807 | 68.703 | 3.320 | 69.389 | 3.907 |
| % Swing Fore | P21 | 42.169 | 4.257 | 43.199 | 5.695 | 40.508 | 1.686 | 39.978 | 1.880 |
| | P24 | 38.215 | 3.766 | 38.539 | 3.937 | 39.534 | 1.686 | 39.400 | 1.619 |
| | P27 | 38.400 | 3.548 | 38.213 | 3.599 | 39.887 | 1.686 | 39.950 | 1.604 |
| | P30 | 39.008 | 3.452 | 38.241 | 4.433 | 39.689 | 1.686 | 40.291 | 1.952 |
| % Swing Hind | P21 | 37.107 | 3.078 | 35.418 | 3.734 | 35.158 | 3.320 | 35.763 | 3.782 |
| | P24 | 35.959 | 2.714 | 35.399 | 2.556 | 33.368 | 3.320 | 33.522 | 3.343 |
| | P27 | 35.276 | 2.556 | 35.533 | 2.356 | 31.079 | 3.320 | 31.007 | 3.318 |
| | P30 | 35.277 | 2.478 | 36.594 | 2.807 | 31.297 | 3.320 | 30.611 | 3.907 |
| Absolute Paw Angle Fore | P21 | 5.685 | 4.549 | 4.536 | 5.791 | 4.795 | 2.778 | 4.595 | 3.253 |
| | P24 | 8.966 | 3.978 | 8.481 | 4.078 | 2.466 | 2.778 | 2.415 | 2.804 |
| | P27 | 5.897 | 3.762 | 6.119 | 3.790 | 3.113 | 2.778 | 3.137 | 2.778 |
| | P30 | 8.468 | 3.580 | 9.347 | 4.292 | 2.632 | 2.778 | 2.858 | 3.378 |
| Absolute Paw Angle Hind | P21 | 24.467 | 4.069 | 22.994 | 5.135 | 4.526 | 1.851 | 4.895 | 2.146 |
| | P24 | 22.955 | 3.560 | 22.439 | 3.573 | 4.337 | 1.851 | 4.430 | 1.847 |
| | P27 | 21.659 | 3.364 | 21.969 | 3.315 | 4.374 | 1.851 | 4.330 | 1.830 |
| | P30 | 22.442 | 3.207 | 23.540 | 3.816 | 3.855 | 1.851 | 3.437 | 2.229 |
| Brake Duration Fore | P21 | 0.049 | 0.011 | 0.055 | 0.014 | 0.055 | 0.010 | 0.056 | 0.011 |
| | P24 | 0.046 | 0.010 | 0.047 | 0.010 | 0.057 | 0.010 | 0.057 | 0.010 |
| | P27 | 0.051 | 0.009 | 0.050 | 0.009 | 0.059 | 0.010 | 0.058 | 0.010 |
| | P30 | 0.049 | 0.009 | 0.046 | 0.011 | 0.058 | 0.010 | 0.056 | 0.012 |
| Brake Duration Hind | P21 | 0.023 | 0.013 | 0.025 | 0.017 | 0.022 | 0.006 | 0.022 | 0.007 |
| | P24 | 0.040 | 0.011 | 0.040 | 0.012 | 0.026 | 0.006 | 0.026 | 0.006 |
| | P27 | 0.039 | 0.010 | 0.038 | 0.011 | 0.026 | 0.006 | 0.026 | 0.006 |
| | P30 | 0.044 | 0.010 | 0.043 | 0.012 | 0.028 | 0.006 | 0.027 | 0.007 |
| Gait Symmetry | P21 | 1.009 | 0.029 | 1.006 | 0.036 | 1.002 | 0.022 | 0.999 | 0.025 |
| | P24 | 1.000 | 0.025 | 0.999 | 0.026 | 1.002 | 0.022 | 1.001 | 0.022 |
| | P27 | 0.998 | 0.024 | 0.999 | 0.024 | 1.005 | 0.022 | 1.005 | 0.021 |
| | P30 | 1.001 | 0.022 | 1.003 | 0.027 | 0.997 | 0.022 | 1.001 | 0.026 |

| | | | | | | | | | |
|---|---|---|---|---|---|---|---|---|---|
| Maximal Rate of Paw Contact Change Fore | P21 | 31.023 | 9.421 | 35.140 | 11.930 | 43.887 | 9.579 | 42.929 | 11.112 |
| | P24 | 33.726 | 8.260 | 34.911 | 8.176 | 41.475 | 9.579 | 41.232 | 9.870 |
| | P27 | 37.128 | 7.786 | 36.300 | 7.542 | 41.463 | 9.579 | 41.577 | 9.799 |
| | P30 | 34.384 | 7.476 | 31.385 | 8.953 | 49.956 | 9.579 | 51.043 | 11.466 |
| Maximal Rate of Paw Contact Change Hind | P21 | 90.209 | 22.890 | 103.906 | 26.867 | 88.823 | 16.074 | 84.895 | 19.036 |
| | P24 | 82.738 | 20.023 | 86.646 | 19.128 | 91.074 | 16.074 | 90.078 | 16.988 |
| | P27 | 93.411 | 18.926 | 91.488 | 17.792 | 99.080 | 16.074 | 99.548 | 16.871 |
| | P30 | 85.400 | 18.033 | 76.536 | 19.898 | 110.125 | 16.074 | 114.581 | 19.622 |
| Paw Overlap Distance Hind | P21 | 2.115 | 1.146 | 2.816 | 1.351 | 1.071 | 0.384 | 1.209 | 0.405 |
| | P24 | 2.587 | 1.004 | 2.811 | 0.937 | 1.536 | 0.384 | 1.571 | 0.361 |
| | P27 | 2.529 | 0.947 | 2.344 | 0.868 | 1.742 | 0.384 | 1.726 | 0.359 |
| | P30 | 2.183 | 0.907 | 1.691 | 1.006 | 1.963 | 0.384 | 1.807 | 0.417 |
| Paw Angle CV Fore | P21 | 673.906 | 665.245 | 904.115 | 851.238 | 136.512 | 295.803 | 122.773 | 347.138 |
| | P24 | 255.219 | 582.385 | 338.322 | 586.882 | 249.486 | 295.803 | 246.002 | 299.119 |
| | P27 | 420.303 | 549.753 | 373.145 | 542.982 | 169.963 | 295.803 | 171.600 | 296.327 |
| | P30 | 175.676 | 525.441 | 6.461 | 635.558 | 329.741 | 295.803 | 345.327 | 360.590 |
| Paw Angle CV Hind | P21 | 26.287 | 10.288 | 26.816 | 13.028 | 211.679 | 116.804 | 183.218 | 133.856 |
| | P24 | 26.247 | 9.002 | 26.417 | 9.339 | 156.490 | 116.804 | 149.272 | 115.227 |
| | P27 | 23.143 | 8.504 | 23.022 | 8.689 | 113.253 | 116.804 | 116.643 | 114.143 |
| | P30 | 21.627 | 8.112 | 21.274 | 9.650 | 119.275 | 116.804 | 151.564 | 139.072 |
| Paw Placement Positioning Hind | P21 | 0.468 | 0.144 | 0.525 | 0.186 | 0.300 | 0.082 | 0.311 | 0.094 |
| | P24 | 0.494 | 0.126 | 0.512 | 0.127 | 0.296 | 0.082 | 0.299 | 0.081 |
| | P27 | 0.495 | 0.119 | 0.485 | 0.116 | 0.325 | 0.082 | 0.324 | 0.081 |
| | P30 | 0.519 | 0.115 | 0.478 | 0.142 | 0.324 | 0.082 | 0.311 | 0.098 |
| Peak Paw Area CV Fore | P21 | 11.587 | 3.953 | 11.446 | 5.089 | 10.077 | 2.839 | 9.086 | 3.075 |
| | P24 | 9.257 | 3.457 | 9.219 | 3.598 | 8.138 | 2.839 | 7.887 | 2.646 |
| | P27 | 8.355 | 3.269 | 8.396 | 3.345 | 7.831 | 2.839 | 7.949 | 2.621 |
| | P30 | 8.954 | 3.111 | 9.049 | 3.770 | 6.926 | 2.839 | 8.050 | 3.194 |
| Peak Paw Area CV Hind | P21 | 8.639 | 2.331 | 8.691 | 3.079 | 19.677 | 4.264 | 18.607 | 4.822 |
| | P24 | 7.038 | 2.039 | 7.052 | 2.131 | 14.145 | 4.264 | 13.873 | 4.150 |
| | P27 | 6.054 | 1.928 | 6.045 | 1.974 | 9.860 | 4.264 | 9.988 | 4.111 |
| | P30 | 5.697 | 1.835 | 5.664 | 2.294 | 8.960 | 4.264 | 10.174 | 5.010 |
| Peak Paw Area Fore | P21 | 0.458 | 0.135 | 0.530 | 0.165 | 0.688 | 0.104 | 0.678 | 0.120 |
| | P24 | 0.468 | 0.118 | 0.488 | 0.114 | 0.651 | 0.104 | 0.649 | 0.108 |
| | P27 | 0.514 | 0.111 | 0.500 | 0.106 | 0.634 | 0.104 | 0.635 | 0.107 |
| | P30 | 0.482 | 0.107 | 0.431 | 0.123 | 0.720 | 0.104 | 0.731 | 0.124 |
| Peak Paw Area Hind | P21 | 1.053 | 0.230 | 1.213 | 0.261 | 1.071 | 0.185 | 1.034 | 0.218 |
| | P24 | 1.016 | 0.201 | 1.062 | 0.186 | 1.081 | 0.185 | 1.071 | 0.194 |
| | P27 | 1.118 | 0.190 | 1.098 | 0.173 | 1.128 | 0.185 | 1.133 | 0.193 |
| | P30 | 1.057 | 0.182 | 0.953 | 0.194 | 1.241 | 0.185 | 1.283 | 0.225 |

| | | | | | | | | | |
|---|---|---|---|---|---|---|---|---|---|
| Propulsion Duration Fore | P21 | 0.085 | 0.014 | 0.089 | 0.018 | 0.089 | 0.015 | 0.095 | 0.015 |
| | P24 | 0.104 | 0.012 | 0.105 | 0.012 | 0.092 | 0.015 | 0.093 | 0.013 |
| | P27 | 0.104 | 0.011 | 0.103 | 0.011 | 0.085 | 0.015 | 0.085 | 0.013 |
| | P30 | 0.106 | 0.011 | 0.103 | 0.014 | 0.096 | 0.015 | 0.089 | 0.016 |
| Propulsion Duration Hind | P21 | 0.125 | 0.020 | 0.140 | 0.022 | 0.135 | 0.014 | 0.139 | 0.015 |
| | P24 | 0.115 | 0.017 | 0.120 | 0.015 | 0.138 | 0.014 | 0.139 | 0.013 |
| | P27 | 0.124 | 0.016 | 0.121 | 0.014 | 0.140 | 0.014 | 0.140 | 0.013 |
| | P30 | 0.121 | 0.016 | 0.110 | 0.016 | 0.148 | 0.014 | 0.144 | 0.015 |
| Stance Factor Fore | P21 | 1.000 | 0.086 | 0.987 | 0.113 | 1.018 | 0.060 | 1.014 | 0.071 |
| | P24 | 1.004 | 0.075 | 1.000 | 0.078 | 0.966 | 0.060 | 0.965 | 0.061 |
| | P27 | 0.987 | 0.071 | 0.990 | 0.072 | 0.960 | 0.060 | 0.961 | 0.060 |
| | P30 | 1.001 | 0.068 | 1.010 | 0.085 | 0.988 | 0.060 | 0.993 | 0.073 |
| Stance Factor Hind | P21 | 1.033 | 0.062 | 1.037 | 0.082 | 1.021 | 0.045 | 1.027 | 0.052 |
| | P24 | 1.000 | 0.055 | 1.001 | 0.057 | 1.006 | 0.045 | 1.008 | 0.045 |
| | P27 | 1.034 | 0.052 | 1.033 | 0.053 | 1.004 | 0.045 | 1.003 | 0.045 |
| | P30 | 1.011 | 0.049 | 1.009 | 0.061 | 1.002 | 0.045 | 0.994 | 0.054 |
| Stance Duration Fore | P21 | 0.134 | 0.013 | 0.144 | 0.015 | 0.144 | 0.014 | 0.151 | 0.013 |
| | P24 | 0.149 | 0.011 | 0.152 | 0.010 | 0.148 | 0.014 | 0.150 | 0.011 |
| | P27 | 0.155 | 0.011 | 0.153 | 0.010 | 0.144 | 0.014 | 0.143 | 0.011 |
| | P30 | 0.156 | 0.010 | 0.149 | 0.011 | 0.154 | 0.014 | 0.146 | 0.013 |
| Stance Duration Hind | P21 | 0.148 | 0.015 | 0.164 | 0.012 | 0.157 | 0.013 | 0.161 | 0.013 |
| | P24 | 0.155 | 0.013 | 0.160 | 0.009 | 0.164 | 0.013 | 0.165 | 0.011 |
| | P27 | 0.163 | 0.012 | 0.160 | 0.008 | 0.166 | 0.013 | 0.165 | 0.011 |
| | P30 | 0.165 | 0.012 | 0.154 | 0.009 | 0.175 | 0.013 | 0.171 | 0.013 |
| Stance Width CV Fore | P21 | 19.171 | 9.484 | 20.129 | 12.580 | 14.014 | 3.791 | 13.371 | 4.287 |
| | P24 | 19.819 | 8.298 | 20.111 | 8.661 | 13.911 | 3.791 | 13.748 | 3.789 |
| | P27 | 16.772 | 7.841 | 16.573 | 8.008 | 14.313 | 3.791 | 14.389 | 3.761 |
| | P30 | 17.239 | 7.475 | 16.580 | 9.402 | 14.666 | 3.791 | 15.395 | 4.428 |
| Stance Width CV Hind | P21 | 6.767 | 2.686 | 6.092 | 3.460 | 7.534 | 2.186 | 7.072 | 2.498 |
| | P24 | 8.392 | 2.349 | 8.192 | 2.413 | 7.058 | 2.186 | 6.941 | 2.150 |
| | P27 | 7.274 | 2.221 | 7.413 | 2.239 | 7.382 | 2.186 | 7.437 | 2.130 |
| | P30 | 6.377 | 2.113 | 6.825 | 2.570 | 6.538 | 2.186 | 7.062 | 2.596 |
| Stance Width Fore | P21 | 2.120 | 0.263 | 2.174 | 0.353 | 1.458 | 0.170 | 1.431 | 0.187 |
| | P24 | 1.950 | 0.231 | 1.966 | 0.241 | 1.442 | 0.170 | 1.435 | 0.163 |
| | P27 | 1.981 | 0.217 | 1.972 | 0.221 | 1.426 | 0.170 | 1.430 | 0.162 |
| | P30 | 2.020 | 0.209 | 1.981 | 0.269 | 1.426 | 0.170 | 1.457 | 0.194 |
| Stance Width Hind | P21 | 3.073 | 0.263 | 3.230 | 0.325 | 2.037 | 0.154 | 2.024 | 0.178 |
| | P24 | 2.881 | 0.231 | 2.929 | 0.222 | 2.005 | 0.154 | 2.002 | 0.155 |
| | P27 | 2.994 | 0.217 | 2.964 | 0.203 | 2.053 | 0.154 | 2.054 | 0.153 |
| | P30 | 3.093 | 0.209 | 2.981 | 0.247 | 2.063 | 0.154 | 2.078 | 0.185 |
| Step Angle CV Fore | P21 | 35.756 | 12.442 | 32.232 | 16.187 | 17.879 | 6.945 | 16.293 | 7.992 |
| | P24 | 34.398 | 10.889 | 33.257 | 11.147 | 18.415 | 6.945 | 18.012 | 6.890 |
| | P27 | 30.452 | 10.283 | 31.186 | 10.308 | 19.137 | 6.945 | 19.326 | 6.826 |
| | P30 | 27.408 | 9.817 | 29.872 | 12.096 | 19.285 | 6.945 | 21.085 | 8.301 |

| | | | | | | | | | |
|---|---|---|---|---|---|---|---|---|---|
| Step Angle CV Hind | P21 | 34.271 | 11.444 | 34.406 | 14.936 | 25.094 | 7.546 | 23.511 | 8.428 |
| | P24 | 34.320 | 10.007 | 34.358 | 10.437 | 25.308 | 7.546 | 24.907 | 7.329 |
| | P27 | 32.841 | 9.466 | 32.811 | 9.690 | 21.260 | 7.546 | 21.449 | 7.265 |
| | P30 | 33.102 | 9.004 | 33.021 | 11.088 | 22.675 | 7.546 | 24.471 | 8.738 |
| Step Angle Fore | P21 | 49.302 | 11.285 | 52.597 | 14.717 | 61.505 | 9.316 | 63.163 | 10.672 |
| | P24 | 53.759 | 9.873 | 54.765 | 10.128 | 62.963 | 9.316 | 63.384 | 9.277 |
| | P27 | 52.474 | 9.329 | 51.787 | 9.363 | 62.432 | 9.316 | 62.234 | 9.196 |
| | P30 | 57.773 | 8.894 | 55.500 | 11.002 | 63.263 | 9.316 | 61.382 | 11.065 |
| Step Angle Hind | P21 | 42.038 | 11.684 | 41.826 | 15.312 | 53.026 | 10.122 | 54.715 | 11.500 |
| | P24 | 47.103 | 10.218 | 47.043 | 10.663 | 52.189 | 10.122 | 52.618 | 10.129 |
| | P27 | 45.249 | 9.664 | 45.295 | 9.894 | 52.079 | 10.122 | 51.878 | 10.050 |
| | P30 | 46.800 | 9.194 | 46.943 | 11.379 | 53.779 | 10.122 | 51.863 | 11.889 |
| Stride Frequency Fore | P21 | 4.394 | 0.355 | 4.069 | 0.362 | 4.242 | 0.364 | 4.068 | 0.348 |
| | P24 | 4.235 | 0.311 | 4.140 | 0.251 | 4.161 | 0.364 | 4.116 | 0.304 |
| | P27 | 4.058 | 0.293 | 4.128 | 0.233 | 4.289 | 0.364 | 4.310 | 0.301 |
| | P30 | 4.001 | 0.280 | 4.226 | 0.269 | 4.021 | 0.364 | 4.219 | 0.360 |
| Stride Frequency Hind | P21 | 4.363 | 0.346 | 4.050 | 0.349 | 4.234 | 0.345 | 4.080 | 0.326 |
| | P24 | 4.233 | 0.303 | 4.144 | 0.245 | 4.155 | 0.345 | 4.116 | 0.286 |
| | P27 | 4.072 | 0.286 | 4.141 | 0.228 | 4.274 | 0.345 | 4.292 | 0.284 |
| | P30 | 4.008 | 0.273 | 4.218 | 0.259 | 4.042 | 0.345 | 4.218 | 0.337 |
| Stride Length CV Fore | P21 | 18.025 | 5.681 | 18.566 | 7.396 | 13.991 | 3.606 | 13.711 | 4.195 |
| | P24 | 18.330 | 4.968 | 18.497 | 5.169 | 14.065 | 3.606 | 13.994 | 3.640 |
| | P27 | 16.462 | 4.699 | 16.346 | 4.799 | 15.671 | 3.606 | 15.705 | 3.608 |
| | P30 | 15.639 | 4.470 | 15.273 | 5.490 | 15.701 | 3.606 | 16.018 | 4.351 |
| Stride Length CV Hind | P21 | 12.543 | 4.280 | 14.773 | 5.400 | 12.593 | 3.523 | 12.107 | 4.085 |
| | P24 | 14.708 | 3.747 | 15.392 | 3.697 | 13.661 | 3.523 | 13.538 | 3.535 |
| | P27 | 14.110 | 3.538 | 13.647 | 3.408 | 12.817 | 3.523 | 12.875 | 3.504 |
| | P30 | 13.385 | 3.379 | 11.858 | 4.057 | 13.025 | 3.523 | 13.576 | 4.240 |
| Stride Length Fore | P21 | 4.658 | 0.387 | 5.012 | 0.390 | 4.839 | 0.420 | 5.029 | 0.400 |
| | P24 | 4.830 | 0.339 | 4.933 | 0.272 | 4.905 | 0.420 | 4.953 | 0.350 |
| | P27 | 5.050 | 0.320 | 4.973 | 0.252 | 4.782 | 0.420 | 4.759 | 0.347 |
| | P30 | 5.096 | 0.305 | 4.852 | 0.290 | 5.097 | 0.420 | 4.882 | 0.414 |
| Stride Length Hind | P21 | 4.706 | 0.381 | 5.050 | 0.385 | 4.853 | 0.401 | 5.024 | 0.382 |
| | P24 | 4.833 | 0.334 | 4.929 | 0.271 | 4.932 | 0.401 | 4.975 | 0.336 |
| | P27 | 5.026 | 0.315 | 4.949 | 0.252 | 4.808 | 0.401 | 4.788 | 0.333 |
| | P30 | 5.103 | 0.301 | 4.871 | 0.286 | 5.111 | 0.401 | 4.917 | 0.396 |
| Swing Duration CV Fore | P21 | 24.963 | 8.376 | 24.328 | 10.744 | 18.839 | 4.734 | 18.133 | 5.463 |
| | P24 | 27.449 | 7.326 | 27.244 | 7.614 | 20.257 | 4.734 | 20.078 | 4.726 |
| | P27 | 26.440 | 6.927 | 26.593 | 7.080 | 21.294 | 4.734 | 21.378 | 4.684 |
| | P30 | 23.839 | 6.593 | 24.245 | 7.958 | 21.123 | 4.734 | 21.924 | 5.670 |

| | | | | | | | | | |
|---|---|---|---|---|---|---|---|---|---|
| Swing Duration CV Hind | P21 | 19.867 | 6.699 | 21.937 | 8.809 | 21.423 | 5.306 | 21.023 | 6.184 |
| | P24 | 19.988 | 5.866 | 20.623 | 6.025 | 19.278 | 5.306 | 19.177 | 5.351 |
| | P27 | 19.781 | 5.536 | 19.365 | 5.550 | 17.737 | 5.306 | 17.785 | 5.302 |
| | P30 | 20.710 | 5.295 | 19.275 | 6.628 | 17.538 | 5.306 | 17.992 | 6.418 |
| Swing Duration Fore | P21 | 0.098 | 0.015 | 0.109 | 0.018 | 0.098 | 0.008 | 0.101 | 0.009 |
| | P24 | 0.093 | 0.013 | 0.096 | 0.013 | 0.097 | 0.008 | 0.098 | 0.008 |
| | P27 | 0.097 | 0.012 | 0.095 | 0.012 | 0.095 | 0.008 | 0.095 | 0.008 |
| | P30 | 0.100 | 0.012 | 0.093 | 0.014 | 0.101 | 0.008 | 0.097 | 0.009 |
| Swing Duration Hind | P21 | 0.088 | 0.010 | 0.089 | 0.013 | 0.086 | 0.013 | 0.090 | 0.014 |
| | P24 | 0.087 | 0.009 | 0.087 | 0.009 | 0.082 | 0.013 | 0.084 | 0.012 |
| | P27 | 0.088 | 0.008 | 0.088 | 0.008 | 0.075 | 0.013 | 0.074 | 0.012 |
| | P30 | 0.090 | 0.008 | 0.089 | 0.010 | 0.080 | 0.013 | 0.075 | 0.014 |

## 4. Supplementary Discussion

*Strain Comparisons:* While different strains of mice often show differential performance in in many behavioral assays (Eisener-Dorman et al., 2011; Keum et al., 2016; Liu et al., 2011; Martin et al., 2014; S. S. Moy et al., 2004; Sheryl S. Moy et al., 2008), our study was not designed to directly compare the two strains tested so our ability interpret any differences is limited. The differences observed may arise from size discrepancies, as FVB mice are consistently larger, or experimental parameters, such as different sample sizes. It is also possible that motor development in the FVB mouse occurs earlier than in the C57 mouse, as suggested by the fact that FVB mice were able to run successfully on the DigiGait at a younger age (P17) than C57 mice (P21, pilot data not shown). Future direct comparison studies are needed to validate strain differences suggested by the present study. In any case, our results provide a reference dataset of gait maturation for two commonly used mouse strains, often employed as the genetic backgrounds for models of disease.

*Body Length:* We also recognized that the rapid growth in body size across the age range examined could confound our gait data, and indeed we found that more than a third of all metrics were significantly influenced by body length. Decreased stride frequency, or cadence, and increased stride length have been suggested as markers of gait maturity in humans, however they are mainly driven by limb lengthening and thus likely do not reflect underlying motor circuit maturation - a more relevant phenotype for brain disorders like NDD. In both strains examined, we initially observed a comparable pattern of decreased stride frequency and increased stride length. However, body length significantly impacted both of these variables, and controlling for this influence revealed that neither stride frequency nor length changed with age from P21 to P30. Thus, we presented the data both before and after controlling for the impact of body size on each gait parameter to highlight the possibility of erroneous interpretations when body size is not considered, and to help define those features that could reflect true differences in CNS circuits rather than simply changes in limb length.

## 5. Supplementary References

Eisener-Dorman, A. F., Grabowski-Boase, L., & Tarantino, L. M. (2011). Cocaine locomotor activation, sensitization and place preference in six inbred strains of mice. *Behavioral and Brain Functions : BBF*, *7*, 29. https://doi.org/10.1186/1744-9081-7-29

Hampton, T. G., Stasko, M. R., Kale, A., Amende, I., & Costa, A. C. S. (2004). Gait dynamics in trisomic mice: Quantitative neurological traits of Down syndrome. *Physiology & Behavior*, *82*(2–3), 381–389. https://doi.org/10.1016/j.physbeh.2004.04.006

Keum, S., Park, J., Kim, A., Park, J., Kim, K. K., Jeong, J., & Shin, H.-S. (2016). Variability in empathic fear response among 11 inbred strains of mice. *Genes, Brain and Behavior*, *15*(2), 231–242. https://doi.org/10.1111/gbb.12278

Liu, L., Coller, J. K., Watkins, L. R., Somogyi, A. A., & Hutchinson, M. R. (2011). Naloxone-precipitated morphine withdrawal behavior and brain IL-1β expression: Comparison of different mouse strains. *Brain, Behavior, and Immunity*, *25*(6), 1223–1232. https://doi.org/10.1016/j.bbi.2011.03.016

Martin, L., Sample, H., Gregg, M., & Wood, C. (2014). Validation of operant social motivation paradigms using BTBR T+tf/J and C57BL/6J inbred mouse strains. *Brain and Behavior*, *4*(5), 754–764. https://doi.org/10.1002/brb3.273

Moy, S. S., Nadler, J. J., Perez, A., Barbaro, R. P., Johns, J. M., Magnuson, T. R., Piven, J., & Crawley, J. N. (2004). Sociability and preference for social novelty in five inbred strains: An approach to assess autistic-like behavior in mice. *Genes Brain Behav*, *3*(5), 287–302. https://doi.org/10.1111/j.1601-1848.2004.00076.x

Moy, Sheryl S., Nadler, J. J., Young, N. B., Nonneman, R. J., Segall, S. K., Andrade, G. M., Crawley, J. N., & Magnuson, T. R. (2008). Social Approach and Repetitive Behavior in Eleven Inbred Mouse Strains. *Behavioural Brain Research*, *191*(1), 118–129. https://doi.org/10.1016/j.bbr.2008.03.015
